# Flow synthesis of oxadiazoles coupled with sequential in-line extraction and chromatography

**DOI:** 10.3762/bjoc.18.27

**Published:** 2022-02-25

**Authors:** Kian Donnelly, Marcus Baumann

**Affiliations:** 1 School of Chemistry, University College Dublin, Science Centre South, Belfield, Dublin 4, Ireland

**Keywords:** chromatography, flow synthesis, in-line purification, oxadiazole, reaction telescoping

## Abstract

An efficient continuous flow process is reported for the synthesis of various 1,3,4-oxadiazoles via an iodine-mediated oxidative cyclisation approach. This entails the use of a heated packed-bed reactor filled with solid K_2_CO_3_ as a base. Using DMSO as solvent, this flow method generates the target heterocycles within short residence times of 10 minutes and in yields up to 93%. Scale-up of this flow process was achieved (34 mmol/h) and featured an integrated quenching and extraction step. Lastly, the use of an automated in-line chromatography system was exploited to realise a powerful flow platform for the generation of the heterocyclic targets.

## Introduction

The application of enabling technologies in chemistry has received a surge in interest in recent years [1–4]. At the forefront of this revolution has been the advent of flow chemistry and its increasing utility in synthetic chemistry [5–8]. This is largely driven by the ability to improve reaction efficiency, safety and provide access to chemistry that was not previously possible [9–11]. Carrying out a reaction in continuous flow mode can improve its efficiency in several ways, including decreasing reaction times, increasing yields, or eliminating tedious unit operations by incorporating them in-line in a telescoped manner. These improvements are typically not limited to the chemistry itself but can also result in the generation of less waste, thus reducing the harmful environmental impact of various processes [12–15]. This has led to a significant development in continuous flow platforms, particularly in industry [16–20].

**Scheme 1:**
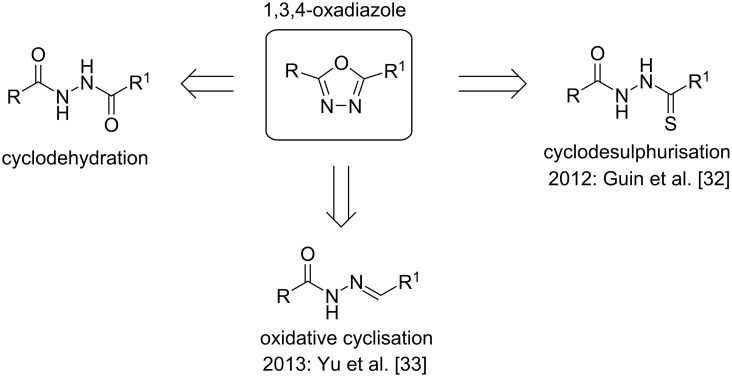
Methods for accessing 1,3,4-oxadiazoles.

1,3,4-Oxadiazoles are biologically relevant 5-membered heterocyclic compounds with various favourable pharmacokinetic properties and have been investigated as potential candidates for antiviral, antifungal and anticancer agents [21–23]. Previous reports of the synthesis of 1,3,4-oxadiazoles in continuous flow focused on the reaction between tetrazoles and carboxylic acids (Huisgen synthesis) [24–25]. Continuous flow technology has also been exploited for the further functionalisation of 1,3,4-oxadiazoles [26]. Various other methods to access this heterocyclic moiety have been reported in the literature, with many focusing on either cyclodehydration or cyclodesulphurisation (Scheme 1) [27]. However, in recent years, there have been a number of oxidative cyclisation protocols reported to access the same 1,3,4-oxadiazole unit [28–33]. In 2012, Guin and co-workers described the iodine-mediated cyclodesulphurisation of thiosemicarbazides, yielding the corresponding 1,3,4-oxadiazoles [32]. Subsequently, Yu and co-workers reported the iodine-mediated oxidative cyclisation of acyl hydrazones to form oxadiazoles [33]. While this method provided the products in high yields, it required the use of super-stoichiometric quantities of iodine, which is potentially toxic and corrosive. This potential toxicity in combination with the requirement for the subsequent removal of excess iodine and potentially biologically active reaction products, led us to explore the implementation of a continuous flow platform to reduce these hazards, while maintaining the high efficiency of the reaction.

## Results and Discussion

We began with the investigation of various oxidative cyclisations of acyl hydrazones to compare the iodine-mediated cyclisation with other oxidative conditions. A range of oxidants was investigated (Table 1), with only *N*-chlorosuccinimide (NCS) [34] and iodine resulting in the formation of product (entries 1 and 2, Table 1). As iodine yielded superior results, we opted to transfer this reaction from batch mode to continuous flow mode.

**Table 1:** Screening of oxidants for cyclisation of acyl hydrazone.

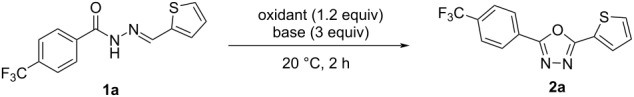				

Entry	Oxidant	Base	Solvent	NMR yield

1	iodine	K_2_CO_3_	DMSO	57%
2	NCS	DBU	DCM	31%
3	sodium periodate	K_2_CO_3_	DMSO	n.d.
4	oxone	K_2_CO_3_	DMSO	n.d.
5	sodium percarbonate	K_2_CO_3_	DMSO	n.d.

The use of an insoluble reagent (e.g. K_2_CO_3_) is generally problematic with continuous flow reactors, due to the high probability of blockages occurring within the reactor tubing. To overcome this, we opted to incorporate a packed bed reactor into the continuous flow setup. The initial setup consisted of a heated glass column (i.d. 7 mm, length 7 cm), packed with K_2_CO_3_, through which a solution of acyl hydrazone and iodine were passed. It was anticipated that the larger excess of K_2_CO_3_ present in the packed bed reactor (when compared to batch mode), in addition to the more efficient heat transfer, would lead to significantly shorter reaction times. Using a flow rate of 0.3 mL/min and a temperature of 100 °C as a starting point (entry 1, Table 2), we began to vary the reaction conditions in view of achieving high yields in short residence times. Through variation of flow rate, we found 0.2 mL/min (approximately 10 minute residence time) to be optimal (entries 1–3, Table 2). Shorter residence times were found to be slightly detrimental to the yield and longer residence times provided no benefit (entries 2 and 3, Table 2). The reaction was found to be sensitive to the quantity of iodine present, with an increase in yield correlating with a larger excess of iodine (entries 4 and 5, Table 2). Variation of reaction temperature identified 100 °C to be optimal, affording the desired product in 90% yield (entry 5, Table 2). A decrease in temperature resulted in a slight decrease in yield, and an increase in decomposition was observed with an increase in temperature despite the short residence times (entries 6 and 7, Table 2). The lack of solubility of the hydrazone substrates limited options with regards to variation in reaction solvent, with adequate solubility only being observed in DMF and DMSO. Despite the slightly lower yield observed using DMF (entry 8, Table 2), it provided the option of a co-solvent system in situations where solubility in DMSO is insufficient.

**Table 2:** Optimisation of iodine-mediated cyclisation in continuous flow mode.

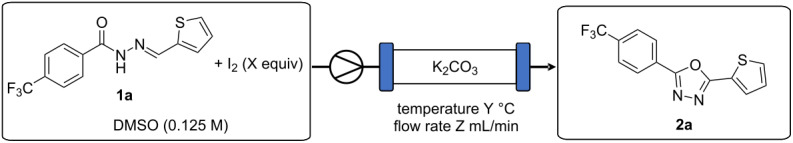				

Entry	Flow rate (Z) (mL/min)	Temperature (Y) (°C)	I_2_ Equivalents (X)	Yield^a^

1	0.3	100	1.1	76%
2	0.2	100	1.1	79%
3	0.1	100	1.1	78%
4	0.2	100	1.2	76%
5	0.2	100	1.5	90%
6	0.2	80	1.5	87%
7	0.2	120	1.5	81%
8^b^	0.2	100	1.5	81%

^a^Yields were determined by ^1^H NMR using 1,3,5-trimethoxybenzene as internal standard. ^b^DMF was used in place of DMSO.

**Scheme 2:**
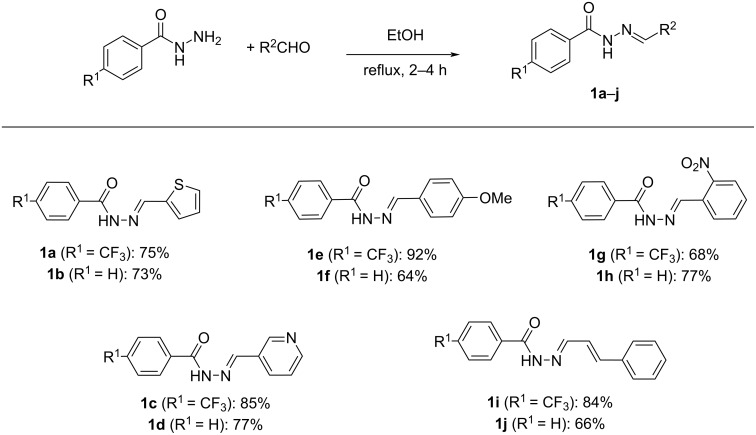
Synthesis of acyl hydrazones **1a**–**j**.

**Scheme 3:**
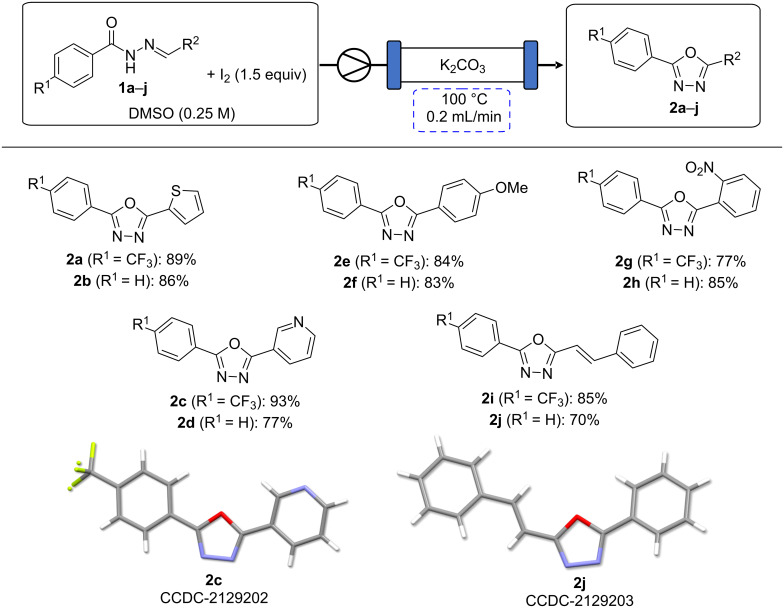
Iodine-mediated cyclisation of hydrazones **1a**–**j** yielding oxadiazoles **2a**–**j***.* Reaction conditions: **1a**–**j** (1 mmol), iodine (1.5 mmol), DMSO (4 mL, 0.25 M), 100 °C, 0.2 mL/min. Reported yields are isolated yields following purification [35].

A variety of acyl hydrazones were subsequently synthesised from the corresponding aldehydes (Scheme 2) and subjected to the optimised flow conditions (Scheme 3). This resulted in full conversion of the substrate in all cases. Both thiophene and pyridine-containing substrates were well tolerated, with slightly higher yields observed in the case of the more electron-deficient CF_3_-substituted system (**2a** and **2c** vs **2b** and **2d**). Both electron-rich and electron-deficient acyl hydrazones afforded high yields, with a slight decrease observed with the presence of the electron-withdrawing CF_3_ group in the case of the electron poor nitro-substituted substrate **2g**. The reaction was also chemoselective in the presence of other oxidisable moieties such as in the cases of **2i** and **2j**. Additionally, the stereoconfiguration of the styryl moiety was maintained as confirmed via the X-ray structure of **2j** (Scheme 3).

**Scheme 4:**
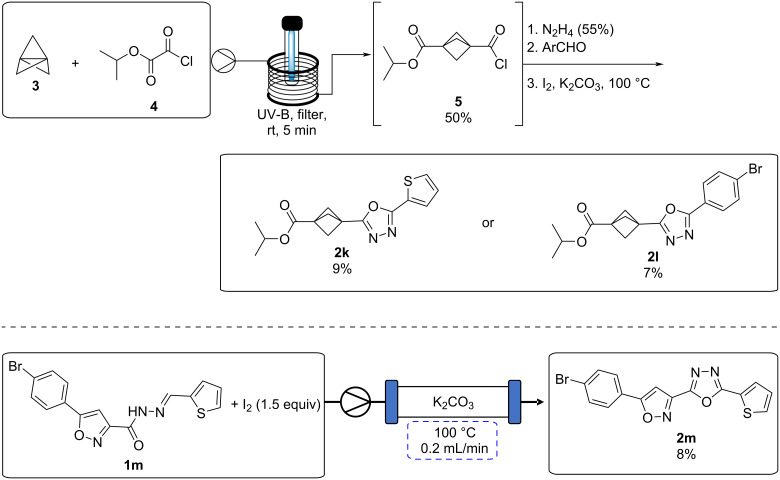
Synthesis of complex oxadiazoles.

As a potential application of our previously reported synthesis of useful bicyclo[1.1.1]pentane (BCP) building blocks [36], we investigated their use in the oxadiazole-forming reaction. The BCP acid chloride **5** was synthesised from [1.1.1]propellane (**3**) via the photochemical reaction with isopropyl 2-chloro-2-oxoacetate (Scheme 4). The corresponding BCP acyl hydrazone was then obtained following treatment of acid **5** with hydrazine hydrate, followed by hydrazone formation with the corresponding aldehyde. When subjected to the reaction conditions, oxadiazoles **2k** and **2l** were obtained in low yield over this multi-step sequence. While unsuitable for large scale reactions, this methodology may prove useful for accessing small quantities of medicinally interesting BCP-1,3,4-oxadiazole compounds for biological testing. Additionally, the reaction of 1,3-substituted isoxazole **1m** under these conditions was investigated. Despite high degrees of decomposition, it was possible to isolate the desired poly-heterocyclic compound **2m** in low yield (Scheme 4).

Having investigated the substrate scope in continuous flow mode, we then moved to probe the scalability of the reaction. A primary advantage of carrying out a reaction in continuous flow is the ease of which it can subsequently be scaled-up, without the need for further extensive optimisation. Oxadiazole product **2j** was chosen as the target molecule for the scale-up reaction due to the potential for further diversification via the embedded alkene. Due to the large quantity of material, an increase in column size was required to house the larger quantity of K_2_CO_3_. As a result of the increased volume of the reactor column (i.d. 15 mm, length 12 cm) a proportional increase in flow rate was necessary to maintain the residence time consistent with our small-scale experiments.

**Scheme 5:**
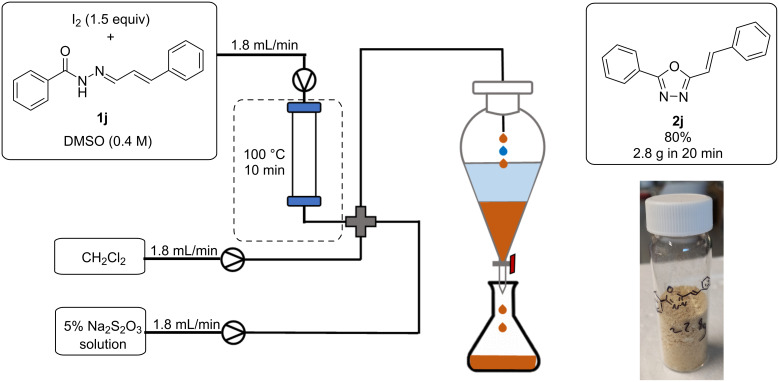
Continuous flow scale-up reaction with in-line quench and extraction.

Following reaction, the excess iodine must be quenched via a wash using sodium thiosulphate solution. To avoid the hazards associated with handling of iodine on multi-gram scale, in addition to eliminating unit operations, an in-line quench and separation was developed. The in-line quench consisted of employing a 4-way mixer, through which sodium thiosulphate solution and an additional organic solvent could be introduced. As the reaction is carried out in DMSO, which is water soluble, an additional organic solvent was required to isolate the desired product from the aqueous phase. There are various examples of in-line separations published in the literature [37], however, many of them involve the use of expensive and complex membrane filters. To reduce cost and increase simplicity we opted to use a ‘home-made’ setup to achieve continuous separation which consisted of a laboratory separating funnel, into which we collect the biphasic reaction output following aqueous workup (Scheme 5). Dichloromethane (DCM) was selected as the organic solvent of choice due to its increased density compared to DMSO and water. Separation in a continuous manner could then be simply achieved by adjusting the outlet tap of the separating funnel such that a constant volume is maintained. To improve throughput, substrate concentration was increased to 0.4 M, and using this setup 2.8 g (11.2 mmol, 80%) of oxadiazole product **2j** could be synthesised in just 20 minutes. This corresponds to a productivity of 8.4 g/h (34 mmol/h). The increase in yield when compared to the small-scale reaction is potentially explained by the reaction reaching steady state during this longer run, thus providing a more accurate indication of the yield. This productivity could potentially be further increased, simply by increasing the volume of the glass column reactor.

With an in-line extraction system in hand, we envisioned a system which would eliminate a further time-consuming unit operation, product purification. Inspired by recent reports of the incorporation of the Advion puriFlash 5.250 system in a continuous flow system [38], we aimed to apply this system to our flow setup. The puriFlash system is an automated liquid chromatography purification system which is capable of purifying mixtures in a continuous fashion by using alternating sample loops and chromatography columns. Initial experiments proved challenging as clean separation could not be achieved with high concentrations (0.4 M) or with large quantities of highly polar DMSO. After some optimisation, satisfactory separation was achieved by decreasing the reaction concentration to 0.2 M, and the final concentration to approximately 0.1 M, through adjustment of the flow rate of DCM in the separation step. Further improvements were realised by increasing the flow rate of the thiosulphate solution to remove sufficient DMSO in view of satisfactory separation being achieved. Due to the high polarity of the reaction mixture, moderately non-polar chromatographic conditions were required (3% EtOAc, 97% cyclohexane at start), with a gradient method being employed. While these conditions successfully provided the desired products in pure form, it must be noted that a short equilibration period between runs was required, thus slightly reducing the throughput of the system.

**Scheme 6:**
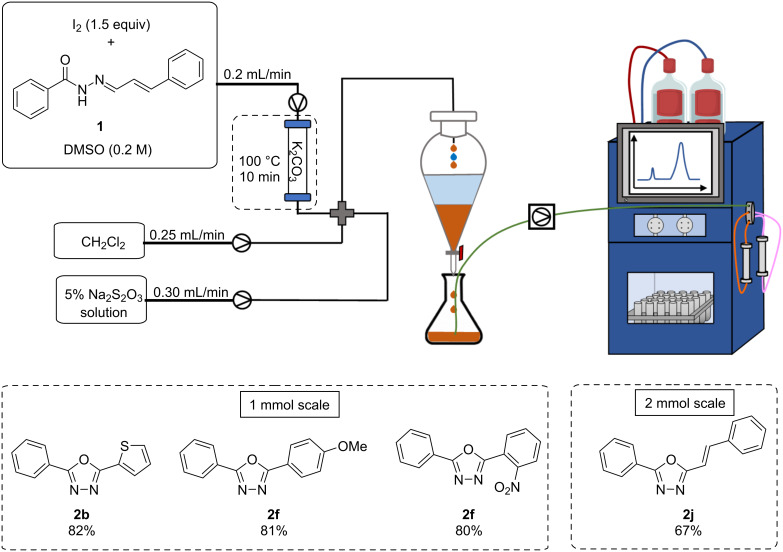
Continuous flow setup equipped with in-line extraction and purification.

With satisfactory chromatography conditions in hand, we moved to test the system using various substrates (Scheme 6). Compounds **2b**, **2f**, and **2h** were all subjected to the reaction conditions and isolated in comparable yields to previous experiments, following in-line quench and in-line purification. In addition to increasing efficiency by removing manual unit operations, the incorporation of the in-line purification system allowed for isolation of pure material in approximately 100 minutes (from substrate vial to pure product) on a 1 mmol scale. To determine the effect of scale on the system, **2j** was processed on a 2 mmol scale (0.5 g) with no loss in efficiency or yield being observed.

While the residence time of the reaction remains constant, incorporating an in-line quench and subsequent purification results in significant reduction in processing times. Typically, performing the quench step manually will take approximately 20–30 minutes when subsequent evaporation of solvent is accounted for. Additionally, manual purification by flash column chromatography can take between 60–120 minutes depending on reaction scale. When added to the reaction time, this results in an estimated total processing time of at least 110 minutes (from substrate vial to pure product) on a 1 mmol scale.

## Conclusion

In summary, we have developed a continuous flow platform for the synthesis of 1,3,4-oxadiazoles in high yields and short residence times of 10 minutes. The incorporation of an in-line extraction reduced the risk of contact with toxic and corrosive iodine in addition to eliminating a tedious unit operation. The reaction was demonstrated to be readily scalable with a productivity of 34 mmol/h being achieved for oxadiazole **2j**. Additionally, the implementation of in-line chromatographic purification provided the desired products in high yields, integrating an additional unit operation and thus increasing efficiency. Through removing two off-line unit operations and carrying out chromatographic purification in-line, further benefits were realised through a reduction in solvent consumption and operator time when compared to the analogous batch process.

## Supporting Information

File 1 Experimental section and analytical data.
