# Supplementary material for: Flow synthesis of oxadiazoles coupled with sequential in-line extraction and chromatography
Source: Beilstein J Org Chem. 2022 Feb 25;18:232–9. doi: 10.3762/bjoc.18.27 (PMC8895036; doi:10.3762/bjoc.18.27)
Supplement: File 1 — Experimental section and analytical data. [file Beilstein_J_Org_Chem-18-232-s001.pdf]

BEILSTEIN JOURNAL OF ORGANIC CHEMISTRY

Supporting Information

for

Flow synthesis of oxadiazoles coupled with sequential in-line extraction and chromatography

Kian Donnelly and Marcus Baumann

Beilstein J. Org. Chem. **2022,** *18,* 232–239. doi:10.3762/bjoc.18.27

Experimental section and analytical data

License and Terms: This is a supporting information file under the terms of the Creative Commons Attribution License (https://creativecommons.org/licenses/by/4.0). Please note that the reuse, redistribution and reproduction in particular requires that the author(s) and source are credited and that individual graphics may be subject to special legal provisions.
The license is subject to the *Beilstein Journal of Organic Chemistry* terms and conditions: (https://www.beilstein-journals.org/bjoc/terms)

Table of contents

1. Materials and methods. S2
2. General Procedures. S3
   2.1 Synthesis of acyl hydrazines. S3
   2.2 Synthesis of acyl hydrazones. S3
   2.3 Synthesis of 1,3,4-oxadiazoles. S3
   2.4 Multigram synthesis of **2j**. S3
3. Continuous flow setup. S4
4. Spectroscopic data:.. S6
5. Copies of NMR spectra:.. S16
6. References:.. S46

1. Materials and methods.

Unless otherwise stated, all solvents were purchased from Fisher Scientific and used without further purification. Substrates and reagents were purchased from Fluorochem or Sigma-Aldrich and used as received.

^{1}H NMR spectra were recorded on 400 MHz, 500 MHz, and 600 MHz instruments and are reported relative to residual solvent: $CDCl_3$ (δ 7.26 ppm), DMSO-d_6 (δ 2.50 ppm). ^{13}C NMR spectra were recorded on the same instruments (100 and 125 MHz) and are reported relative to the corresponding solvent: $CHCl_3$ (δ 77.16 ppm), DMSO-d_6 (39.52 ppm).

Data for ^{1}H NMR are reported as follows: chemical shift (δ/ ppm) (integration, multiplicity, coupling constant (Hz)). Multiplicities are reported as follows: s = singlet, d = doublet, t = triplet, q = quartet, sext = sextet, sept = septet, m = multiplet. Data for ^{13}C NMR are reported in terms of chemical shift (δ/ ppm) and multiplicity (C, CH, CH_2 or CH_3). COSY, HSQC and HMBC experiments were used in the structural assignment.

IR spectra were obtained by use of a Bruker Platinum spectrometer (neat, ATR sampling) with the intensities of the characteristic signals being reported as weak (w, <20% of tallest signal), medium (m, 21–70% of tallest signal) or strong (s, >71% of tallest signal).

High-resolution mass spectrometry was performed using the indicated techniques on a micromass LCT orthogonal time-of-flight mass spectrometer with leucine-enkephalin (Tyr-Gly-Phe-Leu) as an internal lock mass.

Melting points were recorded on a Stuart SMP10 melting point apparatus and are uncorrected. Photochemical experiments were performed on a Vapourtec E-series system with the UV150 photoreactor that is equipped with a medium-pressure Hg lamp (150 W) and used in combination with a low pass filter (see emission spectra below). Continuous flow experiments were performed on a Vapourtec E-series system equipped with heated glass column.

Liquid chromatography was carried out using an Advion puriFlash 5.250 chromatography system equipped with two sets of two PF-15SIHC-F0004 silica columns used as received from Advion. HPLC grade cyclohexane and ethyl acetate were used as received from Sigma-Aldrich without further purification.

2. General procedures.

2.1 Synthesis of acyl hydrazine.

Hydrazine hydrate (55% in water, 8.5 mL, 146 mmol, 5.0 equiv) was added to a stirring solution of methyl benzoate (4.0 g, 29.4 mmol) in ethanol (20 mL, 1.5 M). The mixture was heated to reflux and stirred for 4 hours. After cooling to room temperature, the mixture was diluted with EtOAc (20 mL) and washed with water (20 mL). The aqueous phase was washed with EtOAc (3 × 20 mL). The combined organic phases were washed with brine solution (20 mL) and dried over anhydrous Na_2SO_4. The solvent was evaporated *in vacuo* affording the desired product as a white solid. The material was used for subsequent reactions without further purification.

2.2 Synthesis of acyl hydrazones 1a–j.

Aldehyde (3 mmol) was slowly added to a solution of acyl hydrazine (3 mmol) in EtOH (5 mL, 0.6 M). The mixture was heated to reflux and stirred until completion as indicated by TLC analysis. Upon cooling to 0 °C a precipitate formed, which was isolated *via* vacuum filtration. The solid was washed using cold EtOH and dried under suction. The resulting material was used for subsequent reactions without further purification.

2.3 Synthesis of 1,3,4-oxadiazole.

Acyl hydrazone **1a**–**j** (1 mmol) and iodine (1.5 mmol, 1.5 equiv) were dissolved in DMSO (4 mL, 0.25 M). The mixture was passed through a glass column containing K_2CO_3 heated to 100 °C at a flow rate of 0.2 mL min^{-1} (approximately 10-minute residence time). The output material was collected in a flask containing a stirring mixture of EtOAc and 5% $Na_2S_2O_3$ solution. The phases were separated, and the aqueous phase was washed with EtOAc (3 × 5 mL). The combined organic phases were washed with brine solution (5 mL) and dried over anhydrous Na_2SO_4. The solvent was evaporated *in vacuo* to afford the desired crude product. The crude material was purified by column chromatography.

2.4 Multigram synthesis of 2j.

A solution of **1j** (3.5 g, 14 mmol) and I_2 (5.3 g, 21 mmol, 1.5 equiv) in DMSO (35 mL, 0.4 M) was passed through a glass column containing K_2CO_3 at 100 °C at a flow rate of 1.8 mL min^{-1} (approximately 10-minute residence time). The output material was mixed with separate streams of CH_2Cl_2 (2 mL min^{-1}) and 5% $Na_2S_2O_3$ (2 mL min^{-1}) solution in a 4-way mixer. The biphasic mixture was collected in a separating funnel and the organic phase was collected in a conical flask. The organic solution was washed with brine solution (3 × 15 mL) and the solvent was evaporated *in vacuo* to afford the desired crude product. The crude material was purified by column chromatography.

3. Continuous flow setup.

As depicted below; the continuous flow setup consisted of a Vapourtec E-series reactor equipped with heated glass column, a 4-way mixer, a separating funnel, and an Advion Puriflash 5.250 LC. The glass column (small column: i.d. 7 mm, length 7 cm; larger column: i.d. 15 mm, length 12 cm) was packed with 1 cm of sand and then the remaining volume was filled with K_2CO_3.

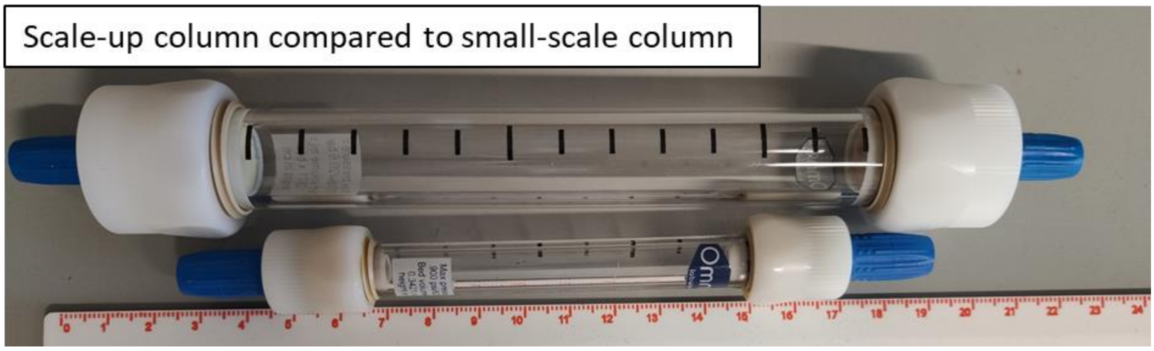

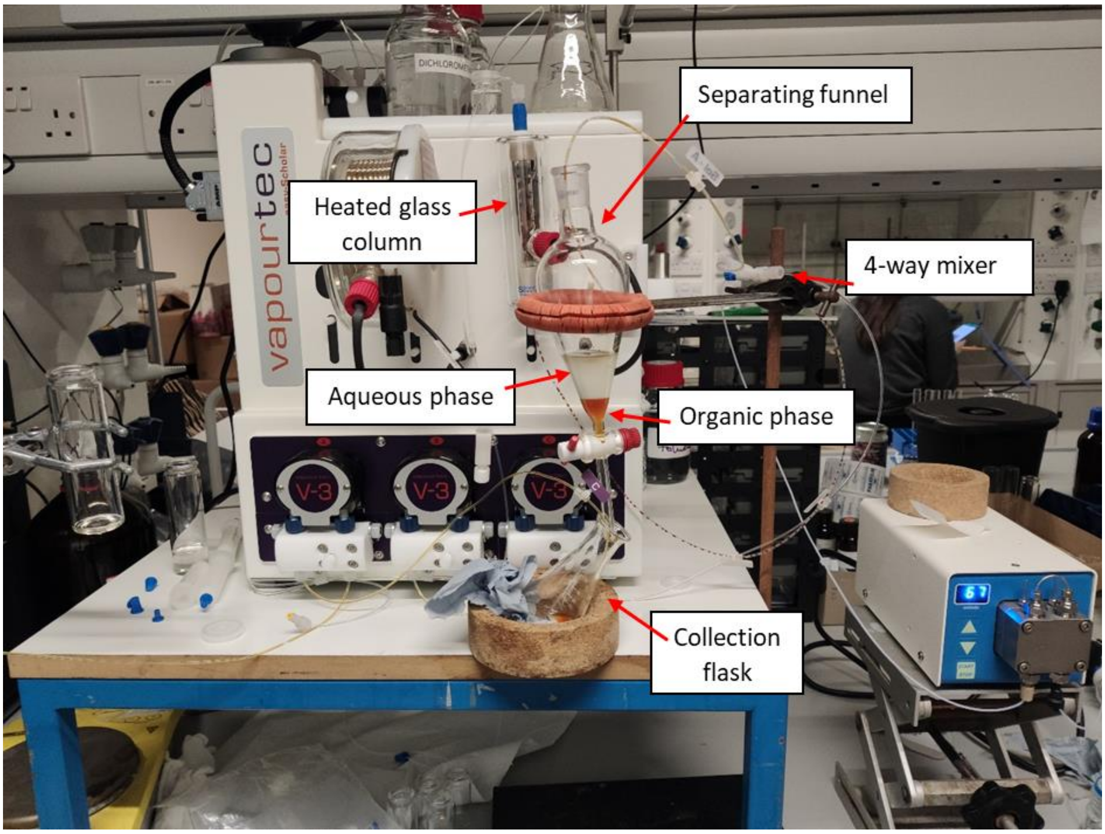

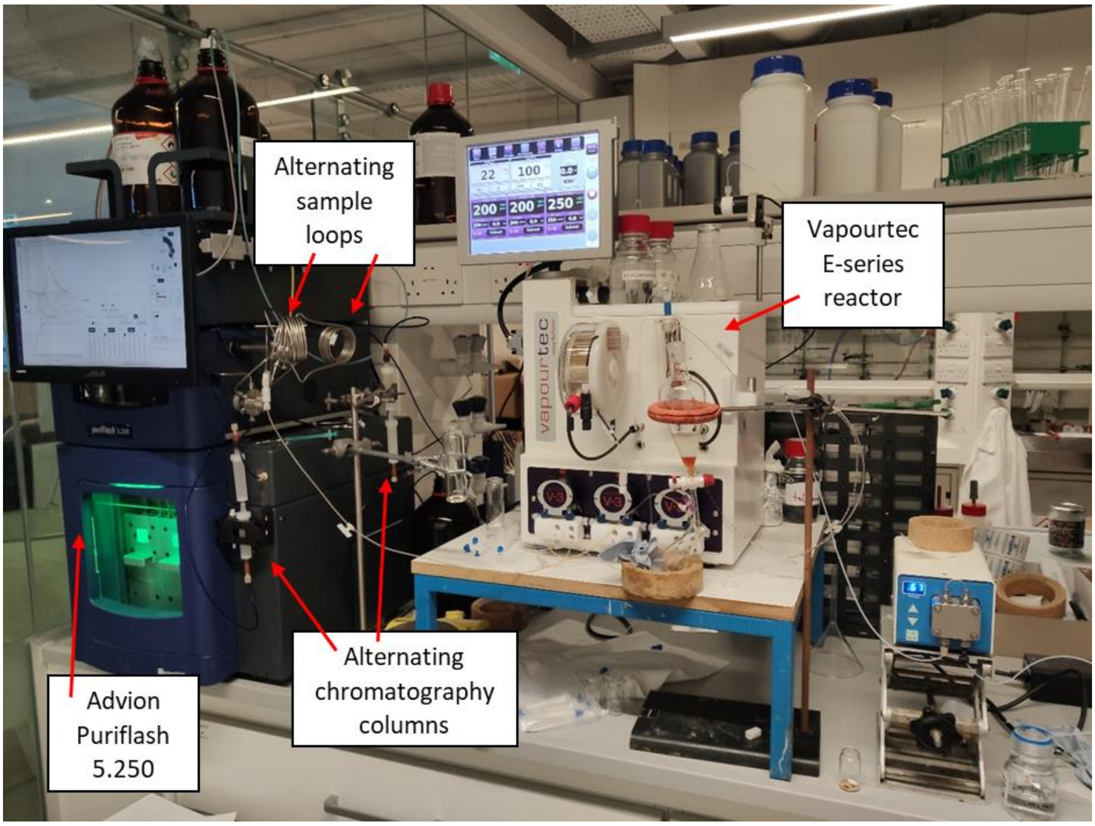
Alternating sample loops
Vapourtec E-series reactor
Advion Puriflash 5.250
Alternating chromatography columns

4. Spectroscopic data:

***N'*-Thiophen-2-ylmethylene)-4-(trifluoromethyl) benzohydrazide (1a):**

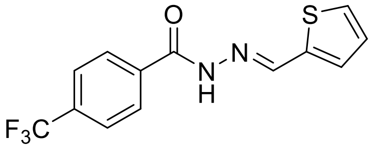

Chemical Formula: $C_{13}H_9F_3N_2OS$
Exact Mass: 298.0388

Yield: 75% (872 mg, 2.92 mmol).

Appearance: Off-white solid.

HR-MS (QTOF) m/z: $[M+H]^+$ Calcd for $C_{13}H_{10}F_3N_2OS^+$: 299.0460, found: 299.0459.

^{1}H NMR (DMSO-*d*$_6$, 500 MHz) δ 11.99 (s, 1H), 8.67 (s, 1H), 8.09 (d, *J* = 8.1 Hz, 2H), 7.91 (d, *J* = 8.1 Hz, 2H), 7.70 (d, *J* = 5.0 Hz, 1H), 7.50 (d, *J* = 3.4 Hz, 1H), 7.16 (m, 1H). **^{13}C NMR (DMSO-*d*$_6$, 125 MHz)** δ 161.8 (C), 143.7 (CH), 138.9 (C), 137.7 (C), 131.5 (q, *J* = 33 Hz, C), 131.4 (CH), 129. 3 (CH), 128.5 (2 CH), 127.9 (CH), 125.5 (q, *J* = 4 Hz, 2 CH), 123.9 (q, *J* = 271 Hz, C). **^{19}F NMR (DMSO-*d*$_6$, 376 Hz)** δ -61.39 (s). **IR (neat)**: v/cm^{-1}: 3180 (br w), 3044 (w), 1645 (s), 1588 (m), 1561 (m), 1429 (w), 1323 (m), 1166 (m), 1126 (s), 1067 (m), 856 (m), 714 (s), 685 (s), 470 (m).

***N'*-(Thiophen-2-ylmethylene)benzohydrazide (1b):**

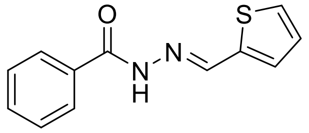

Chemical Formula: $C_{12}H_{10}N_2OS$
Exact Mass: 230.0514

Yield: 73% (501 mg, 2.18 mmol).

Appearance: White solid.

HR-MS (QTOF) m/z: $[M+H]^+$ Calcd for $C_{12}H_{11}N_2OS^+$: 231.0587, found: 231.0589.

^{1}H NMR (DMSO-*d*$_6$, 500 MHz)δ 11.81 (s, 1H), 8.68 (s, 1H), 7.90 (d, *J* = 7.9 Hz, 2H), 7.68 (d, *J* = 5 Hz, 1H), 7.61-7.57 (m, 1H), 7.54-7.51 (m, 2H), 7.47 (d, *J* = 3.7 Hz, 1H), 7.14 (t, *J* = 4.4 Hz, 1H). **^{13}C NMR (DMSO-*d*$_6$, 125 MHz)** δ 163.0 (C), 142.9 (CH), 139.1 (C), 133.4 (C), 131.8 (CH), 130.9 (CH), 129.0 (CH), 128.5 (2 CH), 127.9 (CH), 127.6 (CH). **IR (neat)**: v/cm^{-1}: 3250 (br w), 3086 (w), 1637 (s), 1594 (m), 1551 (s), 1489 (m), 1324 (m), 1280 (s), 1076 (m), 900 (m), 732 (s), 687 (s), 498 (m). Data is consistent with that previously reported [1].

***N'*-(Pyridine-3-ylmethylene)-4-(trifluoromethyl)benzohydrazide (1c):**

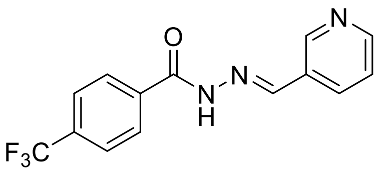

Chemical Formula: $C_{14}H_{10}F_3N_3O$
Exact Mass: 293.0776

Yield: 85% (750 mg, 2.56 mmol).

Appearance: White solid.

HR-MS (QTOF) m/z: $[M+H]^+$ Calcd for $C_{14}H_{11}F_3N_3O^+$:294.0849, found: 294.0848.

^{1}H NMR (DMSO-*d*$_6$, 500 MHz)δ 12.21 (s, 1H), 8.88 (d, *J* = 2.2 Hz, 1H), 8.62 (dd, *J* = 4.8 Hz, 1.7 Hz, 1H), 8.52 (s, 1H), 8.16 (dt, *J* = 8.0 Hz, 1.9 Hz, 1H), 8.13 (d, *J* = 8.1 Hz, 2H), 7.92 (d, *J* = 8.3 Hz, 2H), 7.50 (dd, *J* = 8.0 Hz, 4.8 Hz, 1H). **^{13}C NMR (DMSO-*d*$_6$, 100 MHz)** δ 162.2 (C), 150.9 (CH), 148.9 (CH), 145.9 (CH), 137.1 (C), 133.6 (CH),

131.7 (q, J = 32 Hz, C), 130.1 (C), 128.7 (2 CH), 125.5 (q, J = 4 Hz, 2 CH), 124.1 (CH), 123.9 (q, J = 271 Hz, C). **^{19}F NMR (DMSO-d_6, 376 Hz)** δ -61.39. **IR (neat)**: ν/cm^{-1}: 3192 (br w), 3017 (br w), 1673 (m), 1558 (m), 1416 (m), 1324 (m), 1276 (s), 1164 (m), 1107 (s), 1056 (m), 854 (s), 810 (m), 668 (m), 491 (w).

***N'*-(Pyridine-3-ylmethylene)benzohydrazide (1d):**

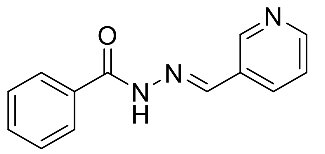

Chemical Formula: $C_{13}H_{11}N_3O$
Exact Mass: 225.0902

Yield: 77% (517 mg, 2.30 mmol).

Appearance: Off-white solid.

HR-MS (QTOF) m/z: $[M+H]^+$ Calcd for $C_{13}H_{12}N_3O^+$: 226.0975, found: 226.0975.

^{1}H NMR (DMSO-d_6, 500 MHz) δ 12.03 (s, 1H), 8.87 (s, 1H), 8.62 (d, J = 4.8 Hz, 1H), 8.52 (s, 1H), 8.15 (d, J = 8.0 Hz, 1H), 7.93 (d, J = 7.7 Hz, 2H), 7.62 – 7.59 (m, 1H), 7.55 – 7.52 (m, 2H), 7.51 – 7.48 (m, 1H). **^{13}C NMR (DMSO-d_6, 125 MHz)** δ 163.3 (C), 150.7 (CH), 148.8 (CH), 145.1 (CH), 133.5 (CH), 133.2 (C), 131.9 (CH), 130.3 (C), 128.5 (2 CH), 127.7 (2 CH), 124.1 (CH). **IR (neat)**: ν/cm^{-1}: 3405 (br w), 3218 (br w), 3056 (br w), 1632 (m), 1611 (m), 1592 (s), 1422 (m), 1294 (s), 1142 (m), 1067 (m), 1024 (m), 954 (w), 693 (s), 595 (m), 505 (m). Data is consistent with that previously reported [2].

***N'*-(4-Methoxybenzylidene)-4-(trifluoromethyl) benzohydrazide (1e):**

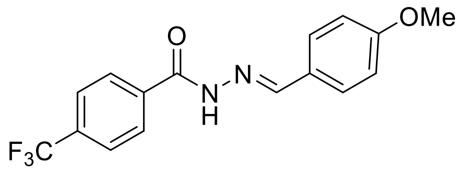

Chemical Formula: $C_{16}H_{13}F_3N_2O_2$
Exact Mass: 322.0929

Yield: 92% (893 mg, 2.77 mmol).

Appearance: White solid.

HR-MS (QTOF) m/z: $[M+H]^+$ Calcd for $C_{16}H_{14}F_3N_2O_2^+$: 323.1002, found: 323.1004.

^{1}H NMR (DMSO-d_6, 500 MHz) δ 11.91 (s, 1H), 8.41 (s, 1H), 8.11 (d, J = 8.2 Hz, 2H), 7.90 (d, J = 8.2 Hz, 2H), 7.70 (d, J = 8.8 Hz, 2H), 7.03 (d, J = 8.8 Hz, 2H), 3.81 (s, 3H). **^{13}C NMR (DMSO-d_6, 125 MHz)** δ 161.8 (C), 161.0 (C), 148.6 (CH), 137.4 (C), 131.5 (q, J = 33Hz, C), 128.9 (2 CH), 128.5 (2 CH), 126.7 (C), 125.5 (q, J = 4 Hz, 2 CH), 123.9 (q, J = 271 Hz, C), 114.4 (2 CH), 55.3 (CH_3). **^{19}F NMR (DMSO-d_6, 470 Hz)** δ -61.40. **IR (neat)**: ν/cm^{-1}: 3168 (br w), 3019 (w), 1643 (m), 1597 (m), 1313 (m), 1258 (m), 1165 (s), 1111 (s), 1017 (m), 915 (m), 823 (s), 685 (s), 534 (m). Data is consistent with that previously reported [3].

***N'*-(4-Methoxybenzylidene)benzohydrazide (1f):**

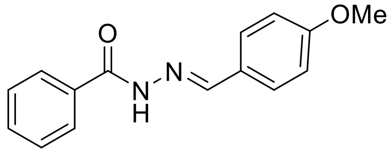

Chemical Formula: $C_{15}H_{14}N_2O_2$
Exact Mass: 254.1055

Yield: 64% (481 mg, 1.89 mmol).

Appearance: White solid.

HR-MS (QTOF) m/z: $[M+H]^+$ Calcd for $C_{15}H_{15}N_2O_2^+$:255.1128, found: 255.1132.

^{1}**H NMR (DMSO-*d*$_6$, 400 MHz)**δ 11.71 (s, 1H), 8.40 (s, 1H), 7.90 (d, *J* = 7.1 Hz, 2H), 7.68 (d, *J* = 8.6Hz, 2H), 7.61 – 7.56 (m, 1H), 7.54 – 7.50 (m, 2H), 7.03 (d, *J* = 8.6 Hz, 2H), 3.81 (s, 3H). ^{13}**C NMR (DMSO-*d*$_6$, 100 MHz)** δ 163.0 (C), 160.9 (C), 147.7 (CH), 133.6 (C), 131.6 (CH) 128.7 (2 CH), 128.5 (2 CH), 127.6 (2 CH), 126.9 (C), 114.4 (2 CH), 55.3 (CH_3). **IR (neat)**: ν/cm^{-1}: 3199 (br w), 3023 (w), 1639 (s), 1603 (m), 1548 (m), 1509 (m), 1283 (m), 1253 (s), 1166 (m), 1023 (s), 831 (s), 695 (s), 664 (s), 606 (m), 534 (m).Data is consistent with that previously reported [4].

***N'*-(2-Nitrobenzylidene)-4-(trifluoromethyl)benzohydrazide (1g):**

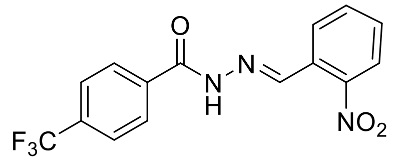

Chemical Formula: $C_{15}H_{10}F_3N_3O_3$
Exact Mass: 337.0674

Yield: 68% **(**692 mg, 2.05 mmol).

Appearance: Yellow solid.

HR-MS (QTOF) m/z: $[M+H]^+$ Calcd for $C_{15}H_{11}F_3N_3O_3^+$:338.0747, found: 338.0747.

^{1}**H NMR (DMSO-*d*$_6$, 400 MHz)**δ 12.39 (s, 1H), 8.89 (s, 1H), 8.14 (d, *J* = 8.2 Hz, 3H), 8.09 (d, *J* = 8.2 Hz, 1H), 7.92 (d, *J* = 8.0 Hz, 2H), 7.82 (t, *J* = 7.6, 1H), 7.69 (t, *J* = 7.8 Hz, 1H).^{13}**C NMR (DMSO-*d*$_6$, 100 MHz)** δ 162.2 (C), 148.3 (C), 143.9 (CH), 136.8 (C), 133.8 (CH), 131.8 (q, *J* = 32 Hz, C), 130.9 (CH), 128.7 (2 CH), 128.6 (C), 128.1 (CH), 125.5 (q, *J* = 4 Hz, 2 CH), 124.7 (CH), 123.9 (q, *J* = 271 Hz, C). ^{19}**F NMR (DMSO-*d*$_6$, 376 Hz)** δ -61.40. **IR (neat)**: ν/cm^{-1}: 3193 (br w), 3056 (br w), 1653 (m), 1551 (m), 1522 (m), 1323 (s), 1161 (m), 1111 (s), 1063 (s), 859 (m), 743 (m), 685 (m). Data is consistent with that previously reported [5].

***N'*-(2-Nitrobenzylidene)benzohydrazide (1h):**

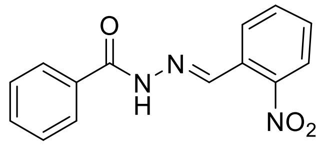

Chemical Formula: $C_{14}H_{11}N_3O_3$
Exact Mass: 269.0800

Yield: 77% (625 mg, 2.32 mmol).

Appearance: Yellow solid.

HR-MS (QTOF) m/z: $[M+H]^+$ Calcd for $C_{14}H_{12}N_3O_3^+$: 270.0873, found: 270.0874.

^{1}**H NMR (DMSO-*d*$_6$, 400 MHz)**δ 12.21 (s, 1H), 8.88 (s, 1H), 8.15 (d, *J* = 7.9 Hz, 1H), 8.08 (d, *J* = 8.1 Hz, 1H), 7.95 (d, *J* = 7.6 Hz, 2H), 7.82 (t, *J* = 7.6 Hz, 1H), 7.68 (t, *J* = 7.8 Hz, 1H), 7.63 – 7.59 (m, 1H), 7.56 – 7.52 (m, 2H). ^{13}**C NMR (DMSO-*d*$_6$, 100 MHz)** δ 163.8 (C), 148.7 (C), 143.3 (CH), 134.2 (CH), 133.2 (C), 132.5 (CH), 131.1 (CH), 129.2 (C), 129.0 (2 CH), 128.4 (CH), 128.2 (2 CH), 125.1 (CH).**IR (neat)**: ν/cm^{-1}: 3155 (br w), 3002 (br w), 1643 (s), 1557 (m), 1522 (s), 1342 (s), 1290 (m), 1150 (m), 1068 (m), 917 (m), 858 (m), 737 (m), 672 (s), 522 (m). Data is consistent with that previously reported [3].

***N'*-((1*E*,2*E*)-3-Phenylallylidene)-4-(trifluoromethyl) benzohydrazide (1i):**

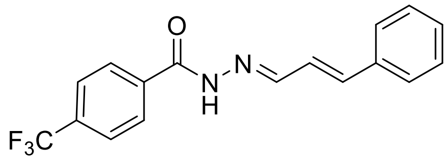

Chemical Formula: $C_{17}H_{13}F_3N_2O$
Exact Mass: 318.0980

Yield: 84% (806 mg, 2.53 mmol)

Appearance: Off white solid.

HR-MS (QTOF) m/z: $[M+H]^+$ Calcd for $C_{17}H_{14}F_3N_2O^+$: 319.1053, found: 319.1058.

^{1}H NMR (DMSO-d_6, 500 MHz)δ 11.93 (s, 1H), 8.25 (dd, *J* = 5.2 Hz, 3.4 Hz, 1H), 8.10 (d, *J* = 8.1 Hz, 2H), 7.90 (d, *J* = 8.2 Hz, 2H), 7.63 (d, *J* = 7.5 Hz, 2H), 7.41 – 7.38 (m, 2H), 7.35 – 7.32 (m, 1H), 7.09 (s, 1H), 7.08 (d, *J* = 2.0 Hz, 1H). **^{13}C NMR (DMSO-d_6, 125 MHz)** δ 161.9 (C), 150.6 (CH), 139.7 (CH), 137.3 (C), 135.9 (C), 131.5 (q, *J* = 32 Hz, C), 129.0 (CH), 128.9 (CH), 128.6 (2 CH), 127.2 (2 CH), 125.50 (2 CH), 125.48 (q, *J* = 4 Hz, 2 CH), 123.9 (q, *J* = 271 Hz, C). **^{19}F NMR (DMSO-d_6, 376 Hz)** δ -61.40. **IR (neat)**: ν/cm^{-1}:3254 (w), 1656 (m), 1623 (m), 1546 (m), 1321 (m), 1126 (s), 1046 (m), 992 (m), 858 (m), 747 (m), 691 (m), 653 (m), 507 (m).

_N'_-((1_E_,2_E_)-3-Phenylallylidene)benzohydrazide (1j):

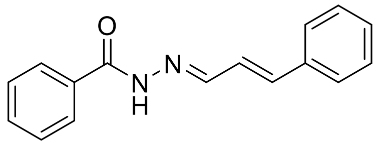

Chemical Formula: $C_{16}H_{14}N_2O$
Exact Mass: 250.1106

Yield: 66% (498 mg, 1.99 mmol).

Appearance: White solid.

HR-MS (QTOF) m/z: $[M+H]^+$ Calcd for $C_{16}H_{15}N_2O^+$: 251.1179, found: 251.1183.

^{1}H NMR (DMSO-d_6, 500 MHz)δ 11.75 (s, 1H), 8.25 (d, *J* = 7.1 Hz, 1H), 7.90 (d, *J* = 7.3 Hz, 2H), 7.63 (d, *J* = 7.4 Hz, 2H), 7.59 (t, *J* = 7.4 Hz, 1H), 7.52 (t, *J* = 7.6 Hz, 2H), 7.39 (t, *J* = 7.5 Hz, 2H), 7.33 (t, *J* = 7.3 Hz, 1H), 7.11 – 7.03 (m, 2H).**^{13}C NMR (DMSO-d_6, 125 MHz)** δ 163.0 (C), 149.8 (CH), 139.0 (CH), 135.9 (C), 133.4 (C), 131.7 (CH), 128.8 (3 CH), 128.5 (2 CH), 127.6 (2 CH), 127.1 (2 CH), 125.7 (CH). **IR (neat)**: ν/cm^{-1}: 3255 (br w), 1643 (m), 1622 (m), 1536 (s), 1488 (m), 1368 (m), 1277 (s), 1132 (m), 1048 (m), 983 (s), 949 (m), 900 (m), 746 (m), 688 (m), 639 (m), 507 (m).

5-(4-Bromophenyl)-_N'_-(thiophen-2-ylmethylene)isoxazole-3-carbohydrazide (1m):

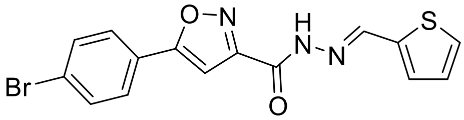

Chemical Formula: $C_{15}H_{10}BrN_3O_2S$
Exact Mass: 374.9677

Yield: 94% (353 mg, 0.94 mmol).

Appearance: White solid.

HR-MS (QTOF) m/z: $[M+H]^+$ Calcd for $C_{15}H_{11}BrN_3O_2S^+$: 375.9750, found: 375.9571.

^{1}H NMR (DMSO-d_6, 500 MHz)δ 8.70 (s, 1H), 7.93 – 7.92 (m, 2H), 7.80 – 7.82 (m, 2H), 7.72 (d, *J* = 5 Hz, 1H), 7.56 (s, 1H), 7.50 (d, *J* = 3.8 Hz, 1H), 7.16 (dd, *J* = 3.6, 1.4 Hz). **^{13}C NMR ($CDCl_3$, 100 MHz)** δ 169.5 (C), 158.9 (C), 154.7(C), 144.7 (CH), 138.6 (C), 132.4 (2 CH), 131.7 (CH), 129.6 (CH), 128.0 (CH), 127.8 (2 CH), 125.3 (C), 124.5 (C), 100.9 (CH). **IR (neat)**: ν/cm^{-1}: 3366 (m), 3210 (w), 1668 (m), 1592 (m), 1557 (s), 1444 (m), 1315 (m), 1239 (s), 1044 (m), 915 (m), 872 (s), 753 (s), 710 (s), 542 (m), 424 (m).

2-(Thiophen-2-yl)-5-(4-(trifluoromethyl)phenyl)-1,3,4-oxadiazole (2a):

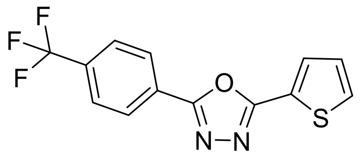

Chemical Formula: $C_{13}H_7F_3N_2OS$
Exact Mass: 296.0231

Yield: 89% (266 mg, 89%)

Appearance: Pale yellow crystalline solid.

Melting range: 135-138°C (CH_2Cl_2).

R$_f$: 0.38 (3:1 cyclohexane: ethyl acetate).

HR-MS (QTOF) m/z: $[M+H]^+$ Calcd for $C_{13}H_8F_3NOS^+$297.0304; Found 297.0301.

^{1}H NMR (CDCl$_3$, 400 MHz)δ 8.22 (d, *J* = 8.1 Hz, 2H) 7.85-7.83 (m, 1H), 7.77 (d, *J* = 8.1 Hz, 2H), 7.60-7.58 (m, 1H), 7.21 – 7.18 (m, 1H). **^{13}C NMR (CDCl$_3$, 100 MHz)** δ 162.9 (C), 161.5 (C), 133.4 (q, *J* = 33 Hz, C), 130.8 (CH), 130.3 (CH), 128.4 (CH), 127.3 (2 CH), 126.6 (C), 125.8 (q, *J* = 3Hz, 2 CH), 124.5 (C), 123.3 (q, *J* = 271 Hz, C). **^{19}F NMR (CDCl$_3$, 376 Hz)** δ -63.11 (CF_3). **IR (neat)**: v/cm^{-1}: 3121 (w), 1584 (m), 1558 (m), 1489 (m), 1323 (m), 1154 (m), 1108 (s), 1062 (s), 1016 (m), 846 (s), 714 (s), 693 (m), 474 (m). Data is consistent with that previously reported [6].

2-Phenyl-5-(thiophen-2-yl)-1,3,4-oxadiazole (2b):

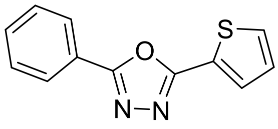

Chemical Formula: $C_{12}H_8N_2OS$
Exact Mass: 228.0357

Yield: 86% (197 mg, 0.86 mmol)

Appearance: Pale yellowcrystalline solid.

Melting range: 118-120°C (CH_2Cl_2).

R$_f$: 0.35 (3:1 cyclohexane: ethyl acetate).

HR-MS (QTOF) m/z: $[M+H]^+$ Calcd for $C_{12}H_9N_2OS^+$:229.0430, found: 229.0432.

^{1}H NMR (CDCl$_3$, 400 MHz)δ 8.10 – 8.07 (m, 2H), 7.80 (dd, *J* = 3.7 Hz, 1.2 Hz, 1H), 7.54 (dd, *J* = 5.0 Hz, 1.2 Hz, 1H), 7.52 – 7.47 (m, 3H), 7.16 (dd, *J* = 5.0 Hz, 3.7 Hz, 1H). **^{13}C NMR (CDCl$_3$, 100 MHz)** δ 164.0 (C), 160.9 (C), 131.8 (2 CH), 130.2 (CH), 129.8 (CH), 129.1 (CH), 128.2 (CH), 126.9 (2 CH), 125.2 (C), 123.7 (C). **IR (neat)**: v/cm^{-1}: 3105 (w), 1584 (m), 1548 (m), 1484 (m), 1446 (m), 1271 (w), 1060 (m), 1024 (m), 843 (m), 715 (s), 684 (s), 506 (m). Data is consistent with that previously reported [7].

2-(Pyridin-3-yl)-5-(4-(trifluoromethyl)phenyl)-1,3,4-oxadiazole (2c):

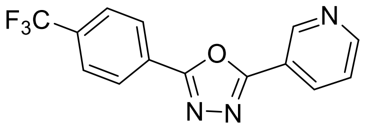

Chemical Formula: $C_{14}H_8F_3N_3O$
Exact Mass: 291.0619

Yield: 93% (272 mg, 0.93 mmol).

Appearance: White crystalline solid.

Melting range: 165-167°C (CH_2Cl_2).

R$_f$: 0.20 (2% MeOH in CH_2Cl_2).

HR-MS (QTOF) m/z: $[M+H]^+$ Calcd for $C_{14}H_9F_3N_3O^+$:292.0692, found: 292.0694.

^{1}H NMR (CDCl$_3$, 400 MHz) δ 9.33 (s, 1H), 8.79 (d, *J* = 4.9 Hz, 1H), 8.43 – 8.39 (m, 1H), 8.24 (d, *J* = 8.2 Hz, 2H), 7.79 (d, *J* = 8.0 Hz, 2H), 7.48 (dd, *J* = 8.0 Hz, 4.8 Hz, 1H). **^{13}C NMR (CDCl$_3$, 100 MHz)** δ 164.0 (C), 163.2 (C), 152.8 (CH), 148.0 (CH), 134.3 (CH), 133.7 (q, *J* = 33 Hz, C), 127.5 (2 CH), 126.8 (d, *J* = 2 Hz, C), 126.3 (q, *J* = 4 Hz, 2 CH), 124.0 (CH), 123.6 (q, *J* = 271 Hz, C), 120.2 (C). **^{19}F NMR (CDCl$_3$, 376 Hz)** δ -63.17. **IR (neat)**: v/cm^{-1}: 3065 (w), 1600 (m), 1558 (m), 1483 (m), 1410 (m), 1324 (m), 1152 (m), 1108 (s), 1064 (m), 965 (m), 845 (s), 696 (s), 593 (m), 472 (m). Data is consistent with that previously reported [8]. **Crystal data (CCDC-2129202):** $C_{14}H_8F_3N_3O$, f.w. 291.23, T = 103 K, triclinic, space group $P2_1/n$ (14), **a** 27.8276(3) **b** 6.02750(10) **c** 7.72790(10)Å, α = 90, β = 109.1900(10), γ = 90°, V = 1224.18Å^3, Z = 4, Dx = 1.580 g cm^{-3}, R-factor (%) 3.1.

2-Phenyl-5-(pyridine-3-yl)-1,3,4-oxadiazole (2d):

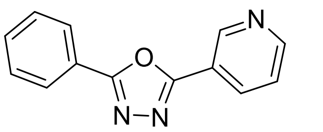

Chemical Formula: $C_{13}H_9N_3O$
Exact Mass: 223.0746

Yield: 77% (171 mg, 0.77 mmol).

Appearance: Off-white crystalline solid.

Melting range: 116-118°C (CH_2Cl_2).

R$_f$: 0.19 (2% MeOH in CH_2Cl_2).

HR-MS (QTOF) m/z: [M+H]$^+$ Calcd for $C_{13}H_{10}N_3O^+$: 224.0818, found: 224.0820.

^{1}H NMR (CDCl$_3$, 500 MHz) δ 9.32 (dd, *J* = 2.3 Hz, 1.0 Hz, 1H), 8.75 (dd, *J* = 4.9 Hz, 1.7 Hz, 1H), 8.39 (ddd, *J* = 8.0 Hz, 1.7 Hz, 0.6 Hz, 1H), 8.12 – 8.09 (m, 2H), 7.56 – 7.48 (m, 3H), 7.46 (ddd, *J* = 8.0 Hz, 4.9 Hz, 1.0 Hz, 1H). **^{13}C NMR (CDCl$_3$, 125 MHz)** δ 165.1 (C), 162.5 (C), 152.5 (CH), 147.9 (CH), 134.2 (CH), 132.1 (CH), 129.2 (2 CH), 127.1 (2 CH), 123.9 (CH), 123.5 (C), 120.5 (C). **IR (neat)**: v/cm^{-1}: 3059 (w), 1599 (m), 1552 (m), 1479 (m), 1449 (m), 1411 (m), 1269 (w), 1067 (m), 1019 (m), 992 (m), 813 (m), 719 (s), 684 (s), 503 (w). Data is consistent with that previously reported [9].

2-(4-Methoxyphenyl)-5-(4-(trifluoromethyl)phenyl)-1,3,4-oxadiazole (2e):

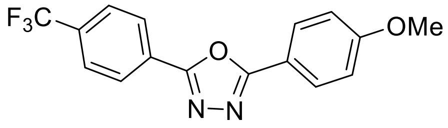

Chemical Formula: $C_{16}H_{11}F_3N_2O_2$
Exact Mass: 320.0773

Yield: 84% (268 mg, 0.84 mmol).

Appearance: White solid.

Melting range: 161-163°C (CH_2Cl_2).

R$_f$: 0.29 (3:1 cyclohexane: ethyl acetate).

HR-MS (QTOF) m/z: [M+H]$^+$ Calcd for $C_{16}H_{12}F_3N_2O_2^+$: 321.0845, found: 321.0844.

^{1}H NMR (CDCl$_3$, 500 MHz) δ 8.24 (d, *J* = 8.1 Hz, 2H), 8.09 – 8.06 (m, 2H), 7.78 (d, *J* = 8.0 Hz, 2H), 7.05 – 7.02 (m, 2H), 3.89 (s, 3H). **^{13}C NMR (CDCl$_3$, 125 MHz)** δ 165.2 (C), 163.1 (C), 162.8 (C), 133.2 (q, *J* = 33 Hz, C), 129.0 (2 CH), 127.4 (d, *J* = 4 Hz, 2 CH), 127.2 (2 CH), 126.2 (q, *J* = 4 Hz, 2CH), 116.1 (C), 114.7 (2 CH), 55.6 (CH_3). **^{19}F NMR (CDCl$_3$, 376 Hz)** δ -63.08. **IR (neat)**: v/cm^{-1}: 3016 (w), 1610 (m), 1494 (m), 1322 (m), 1249 (m), 1164 (m), 1103 (s), 1062 (m), 1027 (m), 967 (m), 844 (s), 705 (m), 618 (m), 521 (m). Data is consistent with that previously reported [10].

2-(4-Methoxyphenyl)-5-phenyl-1,3,4-oxadiazole (2f):

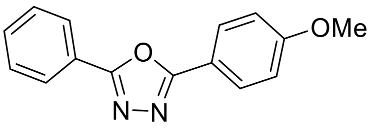

Chemical Formula: $C_{15}H_{12}N_2O_2$
Exact Mass: 252.0899

Yield: 83% (160 mg, 0.63 mmol).

Appearance: Whitesolid.

Melting range: 150-152°C (CH_2Cl_2).

R_f: 0.28 (3:1 cyclohexane: ethyl acetate).

HR-MS (QTOF) m/z: $[M+H]^+$ Calcd for $C_{15}H_{13}N_2O_2^+$: 253.0972, found: 253.0972.

^{1}H NMR (CDCl$_3$, 500 MHz)δ 8.13 – 8.11 (m, 2H), 8.08 – 8.05 (m, 2H), 7.54 – 7.50 (m, 3H), 7.04 – 7.01 (m, 2H), 3.88 (s, 3H). **^{13}C NMR (CDCl$_3$, 125 MHz)** δ 164.7 (C), 164.3 (C), 162.5 (C), 131.6 (CH), 129.2 (2 CH), 128.8 (2 CH), 126.9 (2 CH), 124.2 (C), 116.6 (C), 114.6 (2 CH), 55.6 (CH_3). **IR (neat)**: v/cm^{-1}: 3011 (w), 1614 (m), 1552 (m), 1500 (s), 1313 (m), 1261 (s), 1015 (s), 960 (m), 831 (s), 737 (s), 683 (s), 521 (m). Data is consistent with that previously reported [11].

2-(2-Nitrophenyl)-5-(4-(trifluoromethyl)phenyl)-1,3,4-oxadiazole (2g):

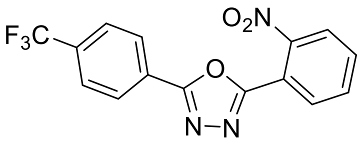

Chemical Formula: $C_{15}H_8F_3N_3O_3$
Exact Mass: 335.0518

Yield: 77% (135 mg, 0.40 mmol).

Appearance: White solid.

Melting range: 168-170°C (CH_2Cl_2).

R_f: 0.22 (3:1 cyclohexane: ethyl acetate).

HR-MS (QTOF) m/z: $[M+H]^+$ Calcd for $C_{15}H_9F_3N_3O^+$:336.0591, found: 336.0596.

^{1}H NMR (CDCl$_3$, 400 MHz)δ 8.19 (d, J = 8.2 Hz, 2H), 8.10 – 8.06 (m, 2H), 7.84 – 7.76 (m, 4H). **^{13}C NMR (CDCl$_3$, 100 MHz)** δ 164.5 (C), 162.0 (C), 148.4 (C), 133.9 (q, J = 33 Hz, C), 133.4 (CH), 133.0 (CH), 131.9 (CH), 127.6 (2 CH), 126.7 (q, J = 1 Hz, C), 126.4 (q, J = 4Hz, 2 CH), 125.0 (CH), 123.6 (q, J = 271 Hz, C), 118.5 (C). **^{19}F NMR (CDCl$_3$, 376 Hz)** δ -63.19. **IR (neat)**: v/cm^{-1}: 3106 (w), 1555 (m), 1525 (s), 1415 (m), 1347 (m), 1320 (s), 1162 (m), 1109 (s), 1064 (s), 1012 (m), 849 (m), 729 (m), 693 (m), 596 (m), 478 (m).

2-(2-Nitrophenyl)-5-phenyl-1,3,4-oxadiazole (2h):

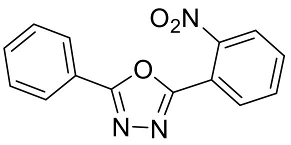

Chemical Formula: $C_{14}H_9N_3O_3$
Exact Mass: 267.0644

Yield: 85% (226 mg, 0.85 mmol)

Appearance: White solid.

Melting range: 121-123°C (CH_2Cl_2).

R_f: 0.23 (3:1 cyclohexane: ethyl acetate).

HR-MS (QTOF) m/z: $[M+H]^+$ Calcd for $C_{14}H_{10}N_3O_3^+$: 268.0717, found: 268.0719.

^{1}H NMR (CDCl$_3$, 400 MHz)δ 8.09 – 8.02 (m, 4H), 7.81 – 7.73 (m, 2 H), 7.58 – 7.49 (m, 3H).**^{13}C NMR (CDCl$_3$, 100 MHz)** δ 165.9 (C), 161.4 (C), 148.4 (C), 133.2 (CH), 132.6 (CH), 132.3 (CH), 131.8 (CH), 129.3 (2 CH), 127.2 (2 CH), 124.8 (CH), 123.4 (C), 118.7 (C). **IR (neat)**: v/cm^{-1}: 3093 (w), 1520 (s), 1446 (m), 1350 (s), 1253 (m), 1062 (m), 962 (m), 854 (m), 789 (m), 720 (s), 684 (s), 490 (w). Data is consistent with that previously reported [12].

(*E*)-2-Styryl-5-(4-(trifluoromethyl)phenyl)-1,3,4-oxadiazole (2i):

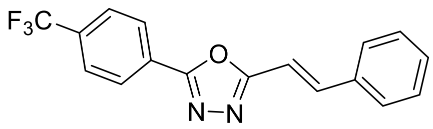

Chemical Formula: $C_{17}H_{11}F_3N_2O$
Exact Mass: 316.0823

Yield: 85% (267 mg, 0.85 mmol)

Appearance: White solid.

Melting range: 155-157°C (CH_2Cl_2).

R$_f$: 0.31 (3:1 cyclohexane: ethyl acetate).

HR-MS (QTOF) m/z: $[M+H]^+$ Calcd for $C_{17}H_{11}F_3N_2O^+$:317.0896, found: 317.0897.

^{1}H NMR ($CDCl_3$, 500 MHz)δ 8.25 (d, *J* = 8.2 Hz, 2H), 7.80 (d, *J* = 8.1 Hz, 2H), 7.68 (d, *J* = 16.1 Hz, 1H), 7.61 – 7.58 (m, 2H), 7.46 – 7.41 (m, 3H), 7.11 (d, *J* = 16.4 Hz, 1H). **^{13}C NMR ($CDCl_3$, 125 MHz)** δ 139.8 (CH), 137.3 (C), 134.7 (C), 133.4 (q, *J* = 33 Hz, C), 130.4 (CH), 129.2 (2 CH), 127.7 (2 CH), 127.4 (2 CH), 126.3 (q, *J* = 4 Hz, 2 CH), 123.7 (q, *J* = 271 Hz, C), 109.8 (CH). **^{19}F NMR ($CDCl_3$, 470 Hz)** δ -63.09. **IR (neat)**: v/cm^{-1}: 3066 (w), 1644 (m), 1519 (m), 1326 (s), 1167 (m), 1109 (s), 1065 (s), 975 (m), 848 (s), 757 (s), 691 (s), 597 (m), 510 (m), 462 (m). Data is consistent with that previously reported [8].

(*E*)-2-Phenyl-5-styryl-1,3,4-oxadiazole (2j):

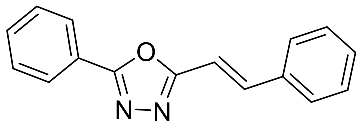

Chemical Formula: $C_{16}H_{12}N_2O$
Exact Mass: 248.0950

Yield: 70% (174 mg, 0.70 mmol).

Appearance: Yellow crystalline solid.

Melting range: 128-129°C (CH_2Cl_2).

R$_f$: 0.36 (3:1 cyclohexane: ethyl acetate).

HR-MS (QTOF) m/z: $[M+H]^+$ Calcd for $C_{16}H_{12}N_2O^+$:249.1022, found: 249.1023.

^{1}H NMR ($CDCl_3$, 400 MHz)δ 8.13 – 8.10 (m, 2H), 7.62 (d, *J* = 16.4 Hz, 1H), 7.58 – 7.55 (m, 2H), 7.55 – 7.49 (m, 3H), 7.44 – 7.36 (m, 3H), 7.09 (d, *J* = 16.4 Hz, 1H). **^{13}C NMR ($CDCl_3$, 100 MHz)** δ 164.4 (C), 164.2 (C), 139.1 (CH), 134.9 (C), 131.9 (CH), 130.1 (CH), 129.22 (2 CH), 129.18 (2 CH), 127.7 (2 CH), 127.1 (2 CH), 124.0 (C), 110.2 (CH). **IR (neat)**: v/cm^{-1}: 3062 (w), 1644 (m), 1545 (m), 1523 (m), 1488 (m), 1446 (m), 1270 (w), 1014 (m), 970 (m), 758 (s), 686 (s), 584 (m), 502 (m). Data is consistent with that previously reported [11]. **Crystal data (CCDC-2129203):** $C_{16}H_{12}N_2O$, f.w. 248.10, T = 100.3 K, triclinic, space group *Pbcn* (60), **a** 11.2646(2) **b** 11.4951(2) **c** 19.4343(3), Å, α = 90, β = 90, γ = 90°, V = 2516.5 Å^3, Z = 8, Dx = 1.311 g cm^{-3}, R-factor (%) 4.56.

Isopropyl 3-(5-(thiophen-2-yl)-1,3,4-ozadiazol-2-yl)bicyclo[1.1.1]pentane-1-carboxylate (2k):

Experimental procedure: To a solution of [1.1.1]propellane (**3**, 1 mmol, 0.2 M in diethyl ether/pentane) was added isopropyl 2-chloro-2-oxoacetate (**4**, 151 mg, 1 mmol) and acetone (2 drops). The mixture was pumped through a Vapourtec E-Series UV-150 photochemical reactor (medium pressure mercury lamp, 10 mL reactor volume) at a flow rate of 2 mL min^{-1} (5 min residence time). The output was cooled to 0 °C and slowly added to a solution of hydrazine hydrate (15 mmol) in CH_2Cl_2 (5 mL, 3M). The mixture was stirred for 15 minutes before warming to room temperature for a further 1 hour. The reaction mixture was then washed with water (10 mL) and the aqueous phase was further extracted with CH_2Cl_2 (5 mL × 3). The solvent was evaporated *in vacuo*

and the resulting residue was dissolved in EtOH (1 mL), to which 2-thiophene carboxaldehyde (0.6 mmol) was added. The mixture was heated at reflux for 2 hours. The solvent was removed *in vacuo* and the crude residue was treated according to general procedure **2.3**.

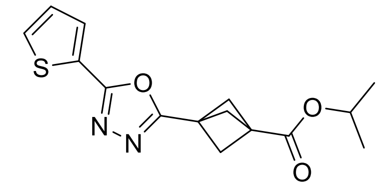

Chemical Formula: $C_{15}H_{16}N_2O_3S$
Exact Mass: 304.0882

Yield: 9% (27 mg, 89 µmol).

Appearance: Yellow amorphous solid.

R_f: 0.21 (3:1 cyclohexane: ethyl acetate).

HR-MS (QTOF) m/z: $[M+H]^+$ Calcd for $C_{15}H_{17}N_2O_3S^+$:305.0954, found: 305.0954.

^{1}H NMR (CDCl$_3$, 500 MHz)δ 7.74 (dd, *J* = 3.7, 1.3 Hz, 1H), 7.54 (dd, *J* = 5.0, 1.2 Hz, 1H), 7.15 (dd, *J* = 5.1, 3.8 Hz, 1H), 5.02 (sept, *J* = 6.3 Hz, 1H), 2.55 (s, 6H), 1.26 (d, *J* = 6.3 Hz). **^{13}C NMR (CDCl$_3$, 125 MHz)** δ 168.5 (C=O), 163.1 (C), 161.2 (C), 130.3 (CH), 130.0 (CH), 128.2 (CH), 125.2 (C), 68.6 (CH), 53.9 (3 CH_2), 39.8 (C), 32.2 (C), 21.9 (2 CH_3). **IR (neat)**: ν/cm^{-1}: 3063 (w), 2978 (w), 1722 (s), 1567 (m), 1466 (w), 1374 (m), 1305 (m), 1205 (s), 1105 (s), 1012 (s), 847 (m), 729 (s), 541 (m), 458 (m).

Isopropyl 3-(5-(4-bromophenyl)-1,3,4-oxadiazol-2-yl)bicyclo[1.1.1]pentane-1-carboxylate (2l):

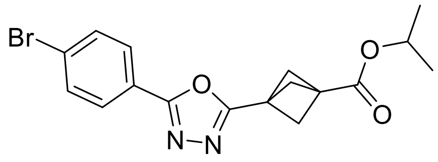

Chemical Formula: $C_{17}H_{17}BrN_2O_3$
Exact Mass: 376.0423

Yield: 7% (19 mg, 51 µmol).

Appearance: White amorphous solid.

HR-MS (QTOF) m/z: $[M+H]^+$ Calcd for $C_{17}H_{18}BrN_2O_3^+$: 377.0495, found: 377.0495.

^{1}H NMR (CDCl$_3$, 400 MHz)δ 7.92 – 7.89 (m, 2H), 7.66 – 7.63 (m, 2H), 5.03 (sept, *J* = 6.2 Hz, 1H), 2.56 (s, 6H), 1.27 (d, *J* = 6.4 Hz, 6H). **^{13}C NMR (CDCl$_3$, 100 MHz)** δ 168.5 (C=O), 164.3 (C), 163.9 (C), 132.6 (2 CH), 128.5 (2 CH), 126.6 (C), 122.9 (C), 68.7 (CH), 54.0 (3 CH_2), 39.9 (C), 32.3 (C), 21.9 (2 CH_3). **IR (neat)**: ν/cm^{-1}: 2979 (w), 1722 (s), 1643 (m), 1478 (m), 1374 (m), 1319 (m), 1282 (s), 1103 (s), 1025 (m), 837 (m), 735 (m), 501 (m), 478 (m).

2-(5-(4-Bromophenyl)isoxazol-3-yl)-5-(thiophen-2-yl)-1,3,4-oxadiazole (2m):

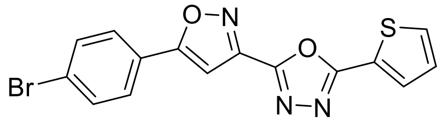

Chemical Formula: $C_{15}H_8BrN_3O_2S$
Exact Mass: 372.9521

Yield: 8% (10 mg, 27µmol).

Appearance: White solid.

HR-MS (QTOF) m/z: $[M+H]^+$ Calcd for $C_{15}H_9BrN_3O_2S^+$: 373.9593, found:373.9595.

^{1}H NMR (CDCl$_3$, 400 MHz)δ 7.94 (dd, *J* = 3.8, 1.2 Hz, 1H), 7.75 – 7.73 (m, 2H), 7.69 – 7.66 (m, 2H), 7.64 (dd, *J* = 5.0, 1.2 Hz, 1H), 7.23 (dd, *J* = 5.0, 3.8 Hz, 1H), 7.21 (s, 1H). **^{13}C NMR (CDCl$_3$, 100 MHz)** δ 171.0 (C), 162.2 (C), 156.5 (C), 151.0 (C), 132.7 (2 CH), 131.5 (C), 131.3 (CH), 128.6 (CH), 127.7 (2 CH),

125.8 (C), 125.3 (C), 124.3 (C), 99.2 (CH). **IR (neat)**: v/cm^{-1}: 3105 (w), 2920 (w), 1614 (m), 1482 (m), 1399 (m), 1226 (m), 1066 (m), 1008 (m), 948 (m), 819 (s), 711 (s), 674 (m), 499 (m).

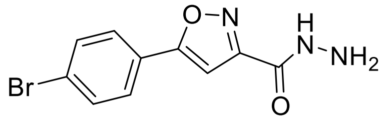

Chemical Formula: $C_{10}H_8BrN_3O_2$
Exact Mass: 280.9800

5-(4-Bromophenyl)isoxazole-3-carbohydrazide (SI1):

Yield: 86% (485 mg, 1.72 mmol).

Appearance: White solid.

HR-MS (QTOF) m/z: $[M+H]^+$ Calcd for $C_{10}H_9BrN_3O_2^+$: 281.9873, found:281.9876.

^{1}H NMR (DMSO-d_6, 400 MHz)δ 10.09 (s, NH), 7.89 – 7.85 (m, 2H), 7.79 – 7.75 (m, 2H), 7.40 (s, 1H), 4.64 (s, NH_2). **^{13}C NMR ($CDCl_3$, 125 MHz)** 169.0 (C), 158.9 (C), 157.7 (C), 132.4 (2 CH), 127.7 (2 CH), 125.5 (C), 124.3 (C), 100.3 (CH). δ **IR (neat)**: v/cm^{-1}:3317 (br w), 1681 (m), 1598 (m), 1521 (m), 1436 (m), 1264 (m), 1103 (m), 944 (m), 815 (s), 707 (m), 666 (m), 601 (s), 489 (m), 430 (m).

5. Copies of NMR spectra:

N′-Thiophen-2-ylmethylene)-4-(trifluoromethyl)benzohydrazide, 1a, 500 MHz, DMSO-d_6:

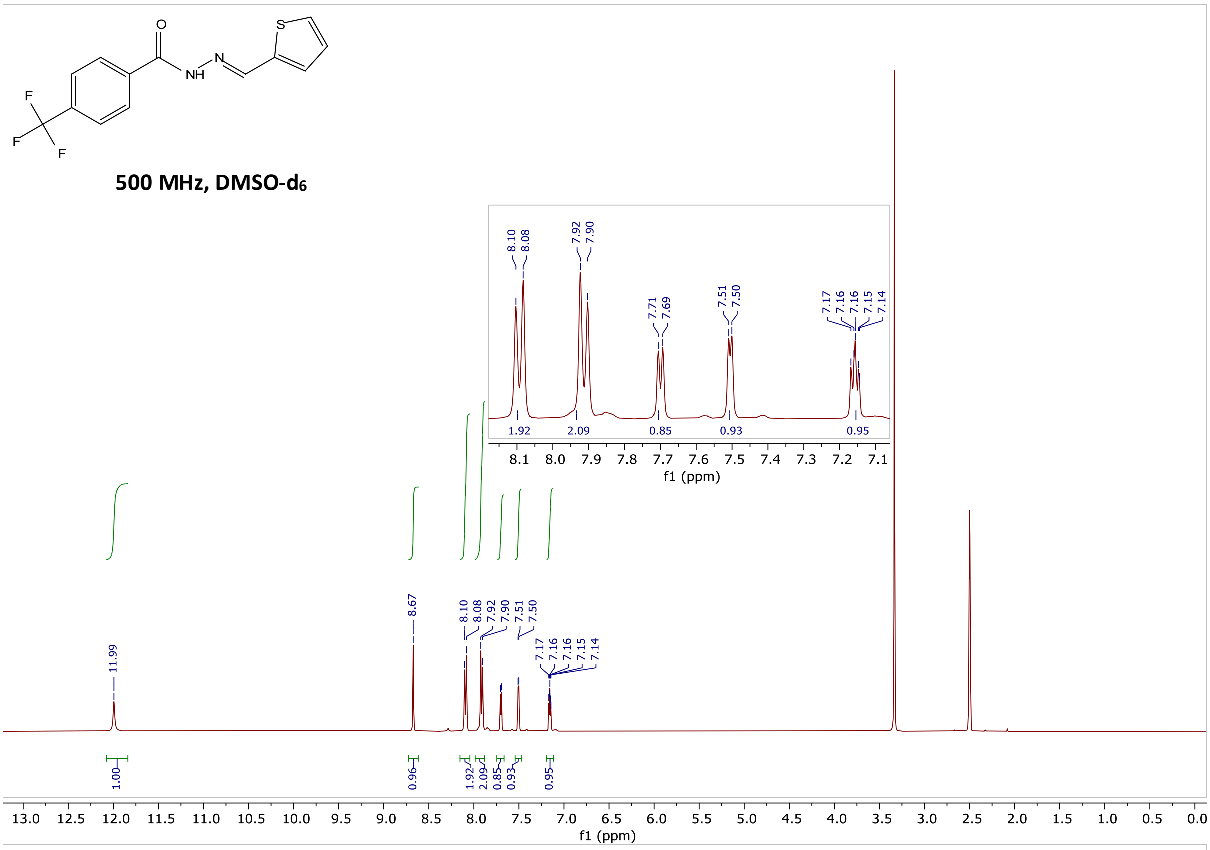

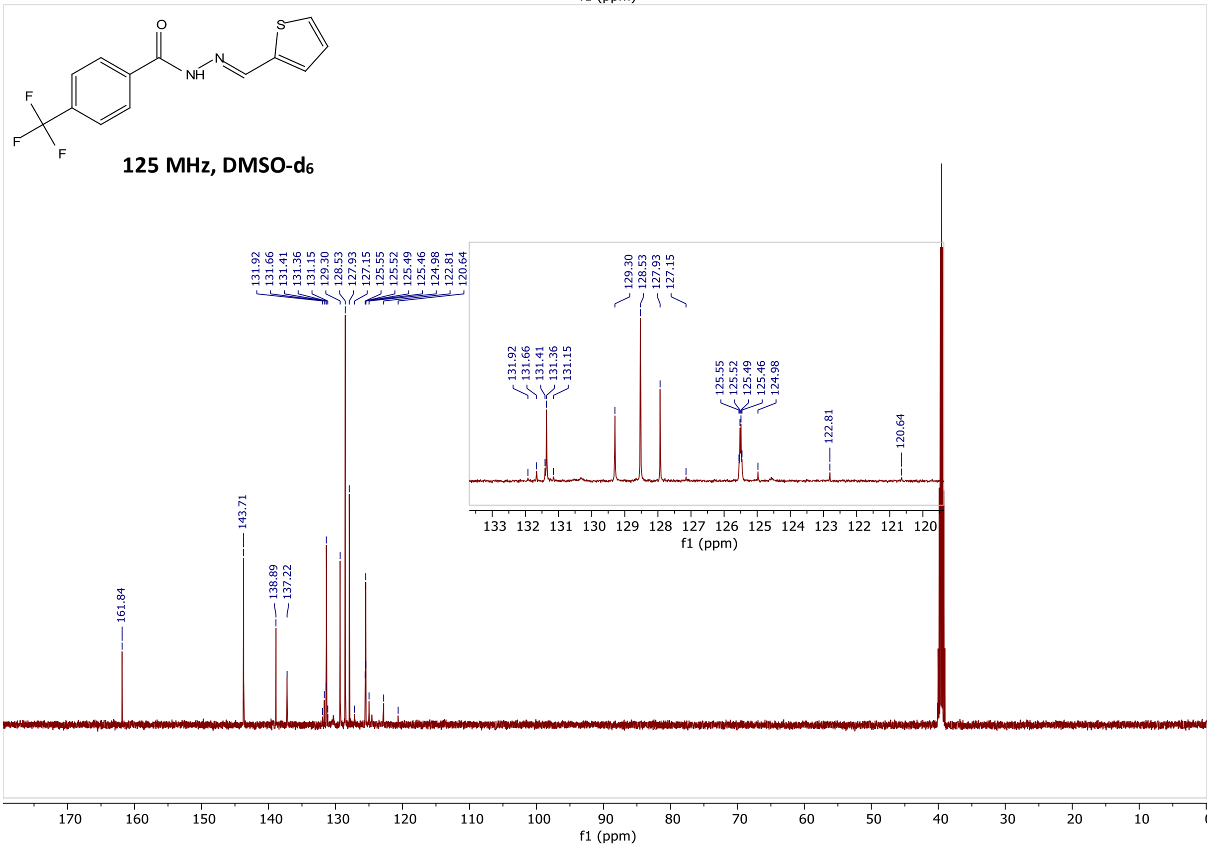

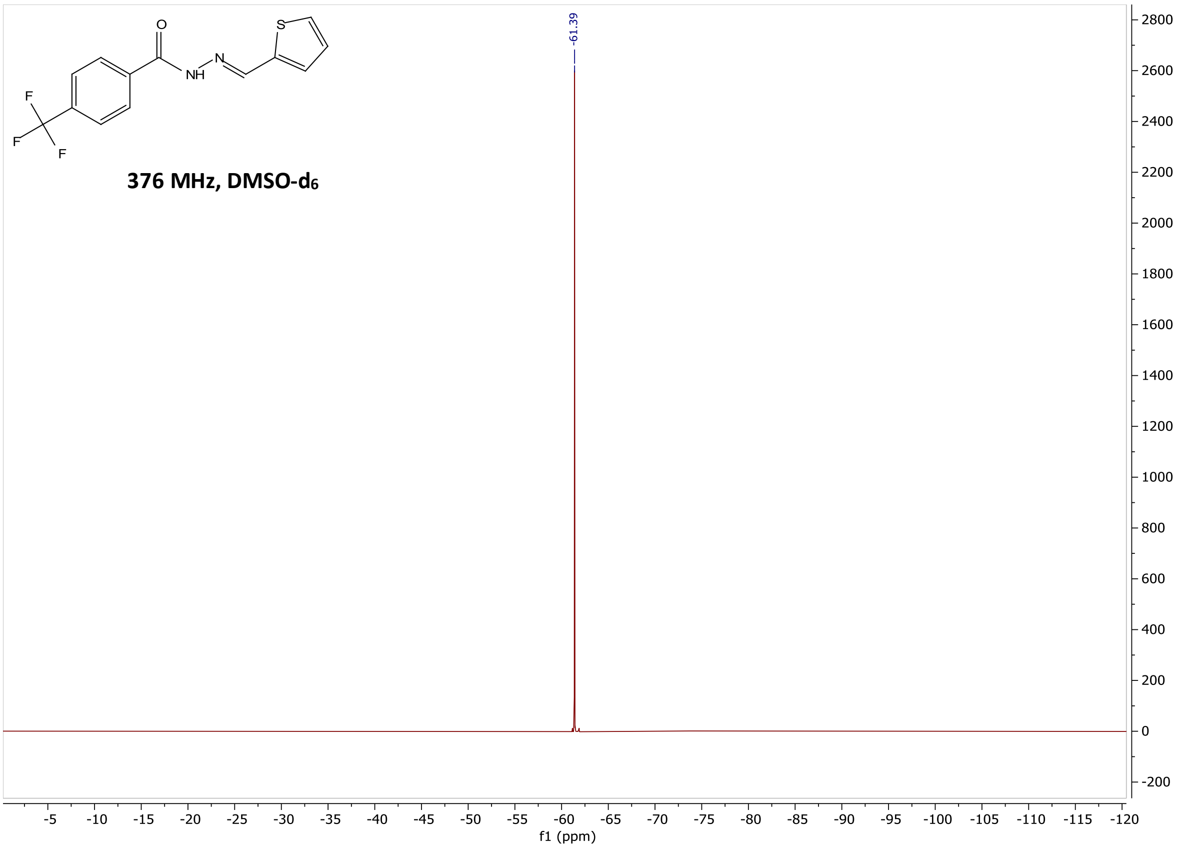

N'-(Thiophen-2-ylmethylene)benzohydrazide, 1b, 500 MHz, DMSO-d_6:

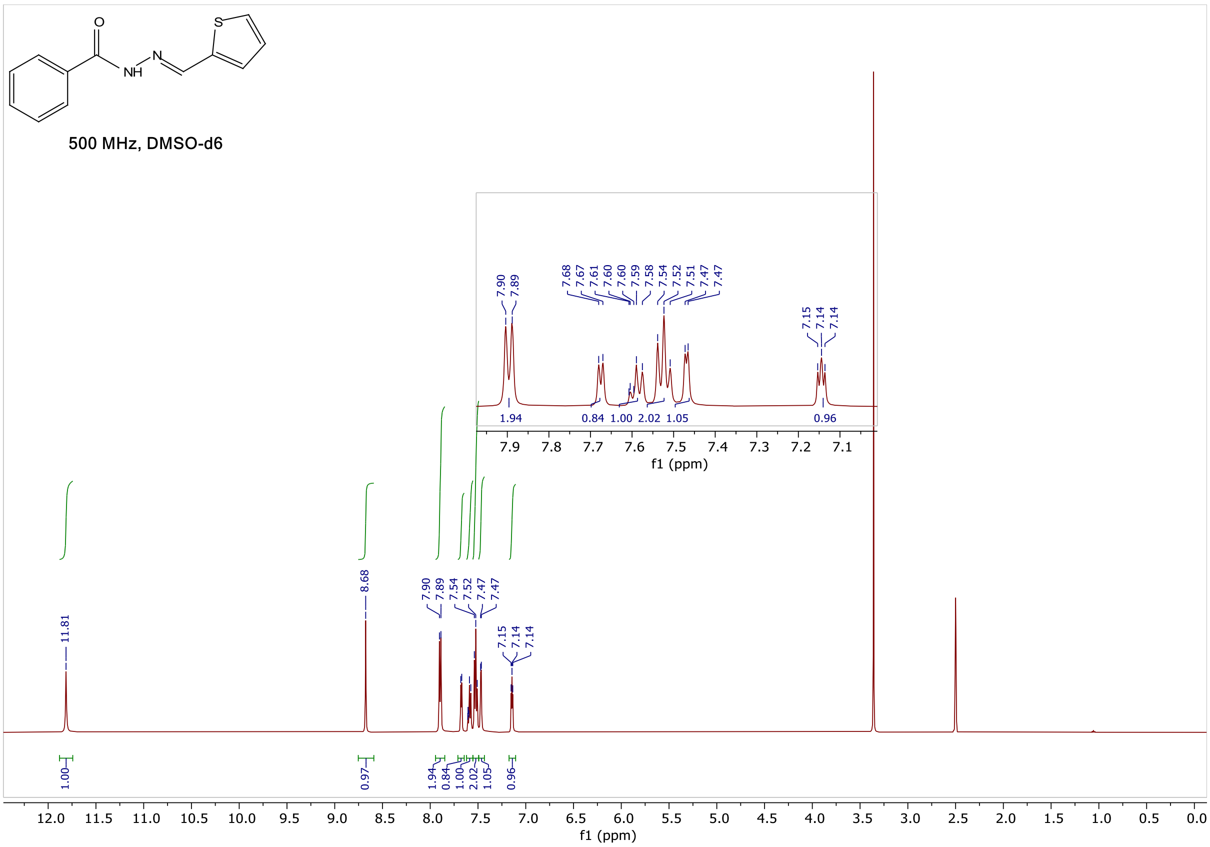

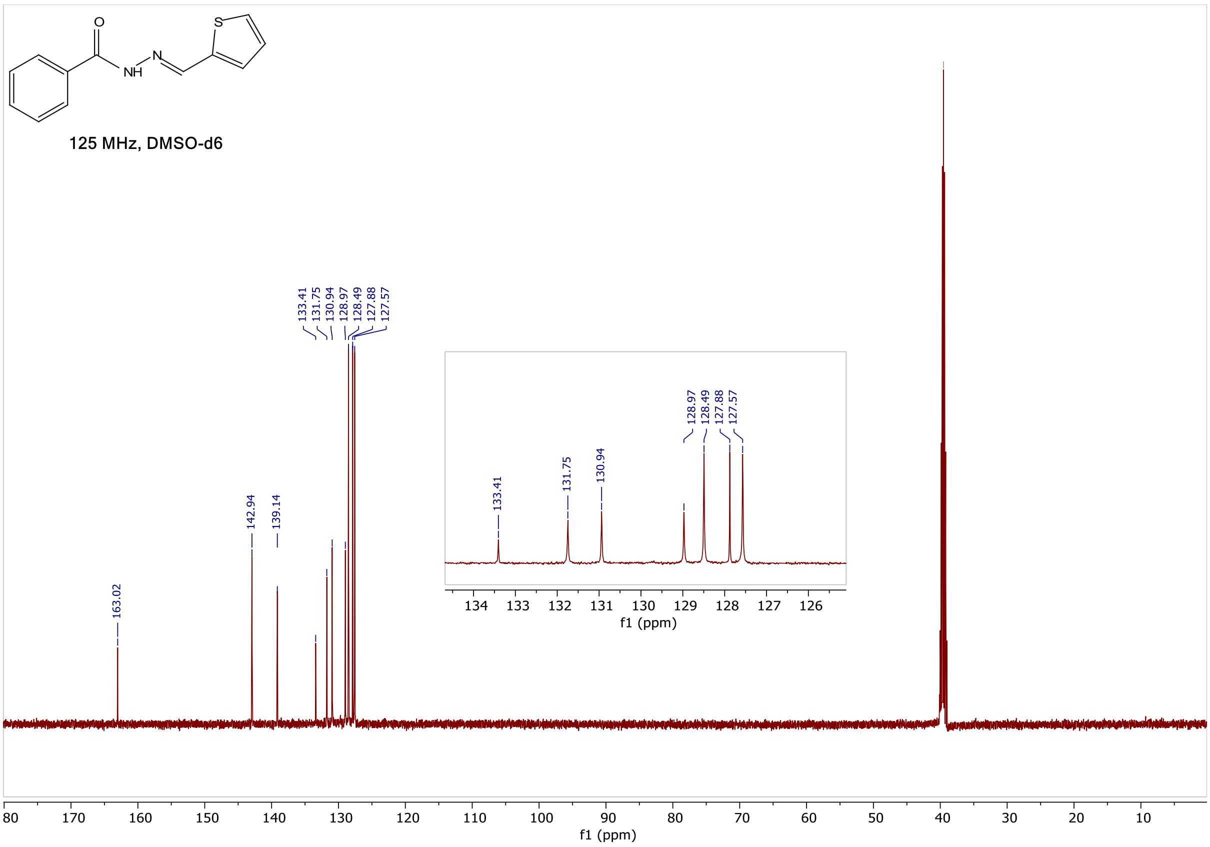

N'-(Pyridine-3-ylmethylene)-4-(trifluoromethyl)benzohydrazide, 1c, 500 MHz, DMSO-d_6:

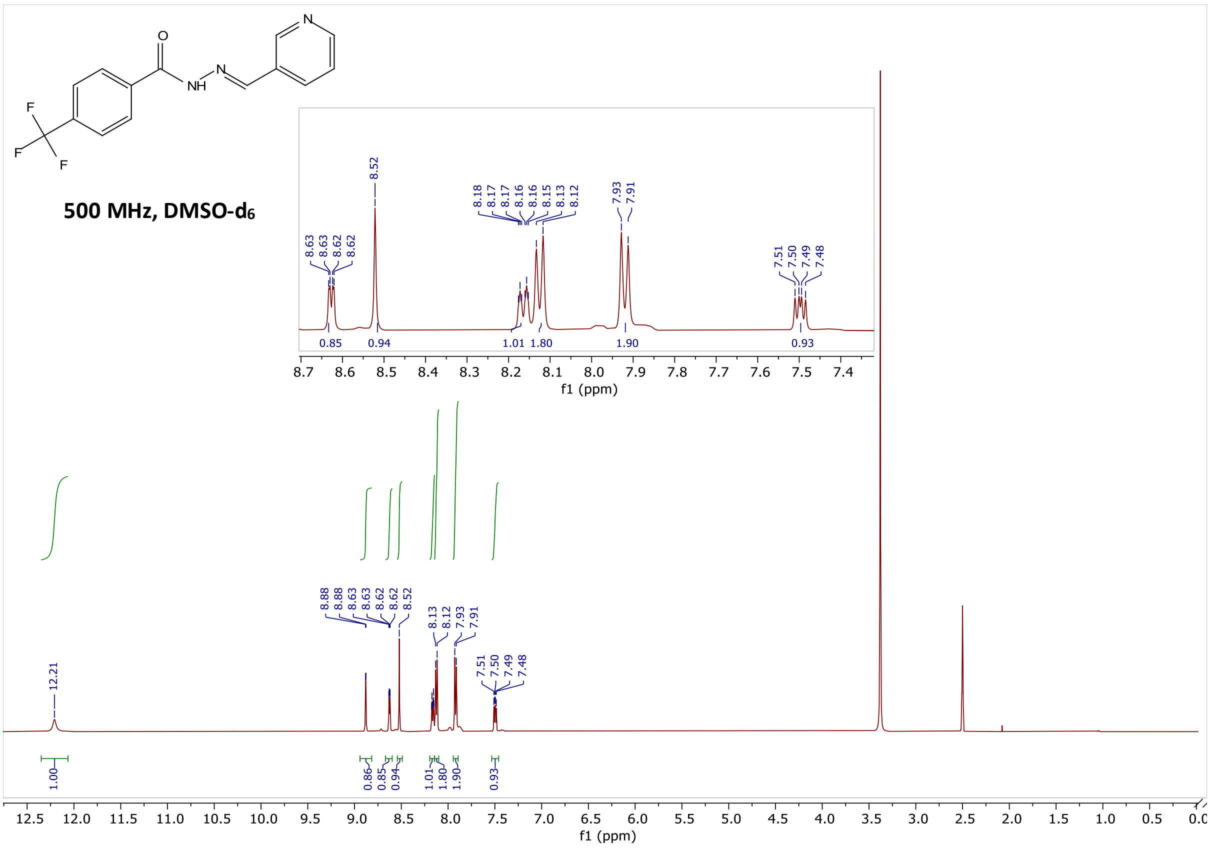

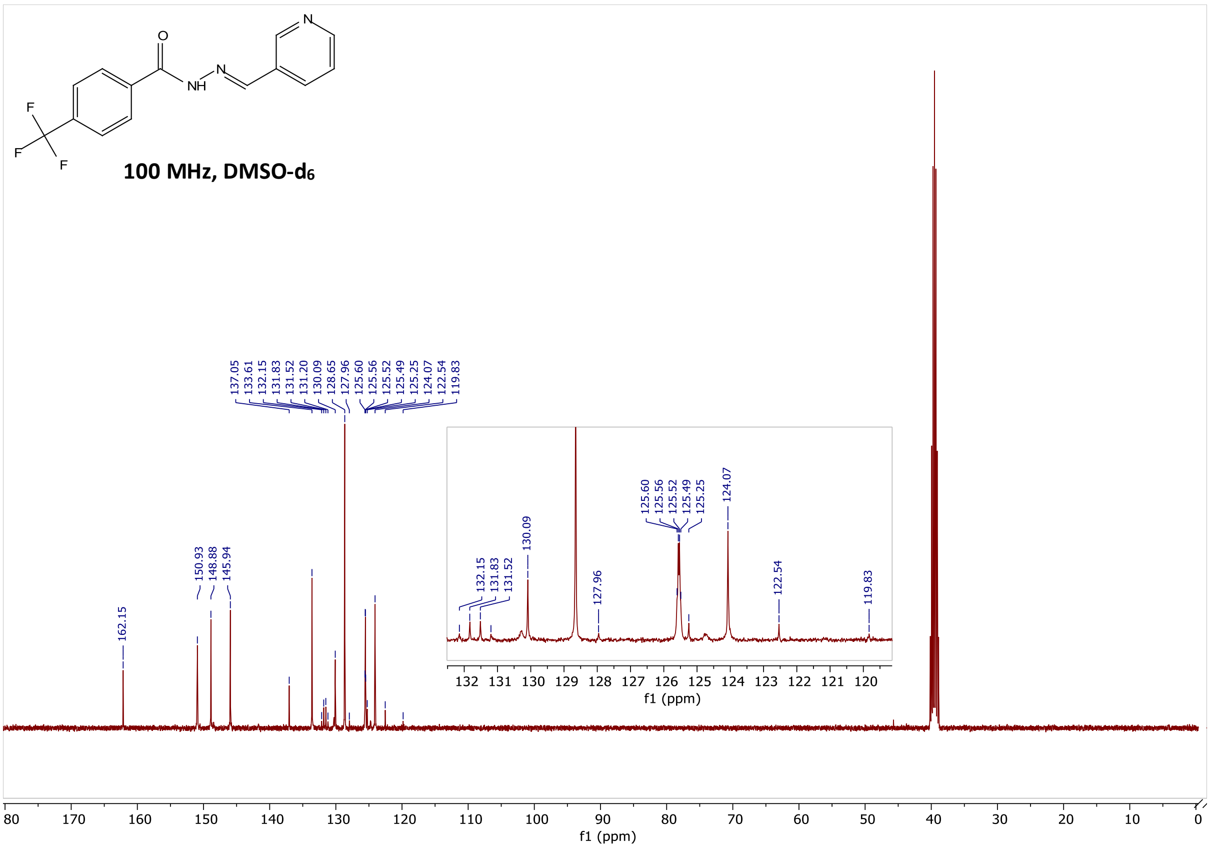

100 MHz, DMSO-d6
162.15
150.93
148.88
145.94
137.05
133.61
132.15
131.83
131.52
131.20
130.09
128.65
127.96
125.60
125.56
125.52
125.49
125.25
124.07
122.54
119.83
f1 (ppm)

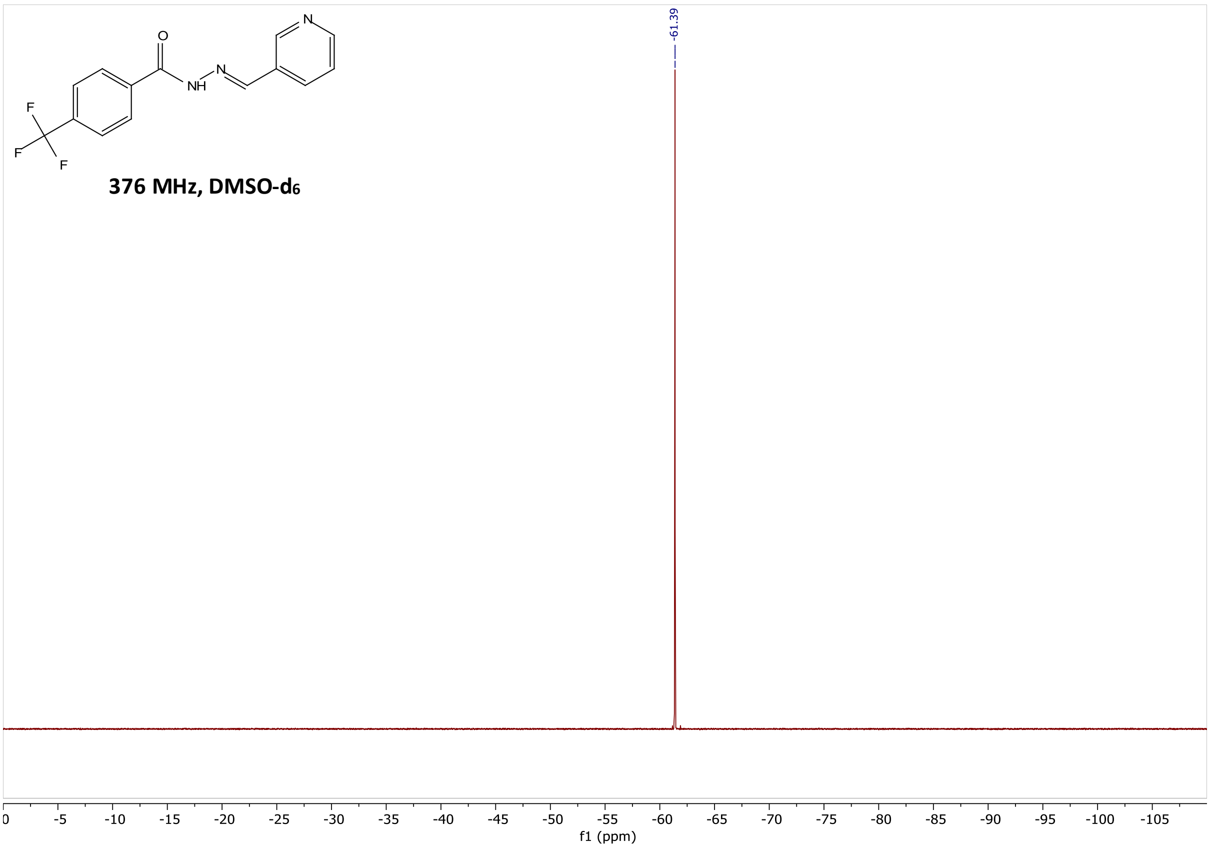

376 MHz, DMSO-d6
-61.39
f1 (ppm)

***N'*-(Pyridine-3-ylmethylene)benzohydrazide, 1d, 500 MHz, DMSO-*d*$_6$:**

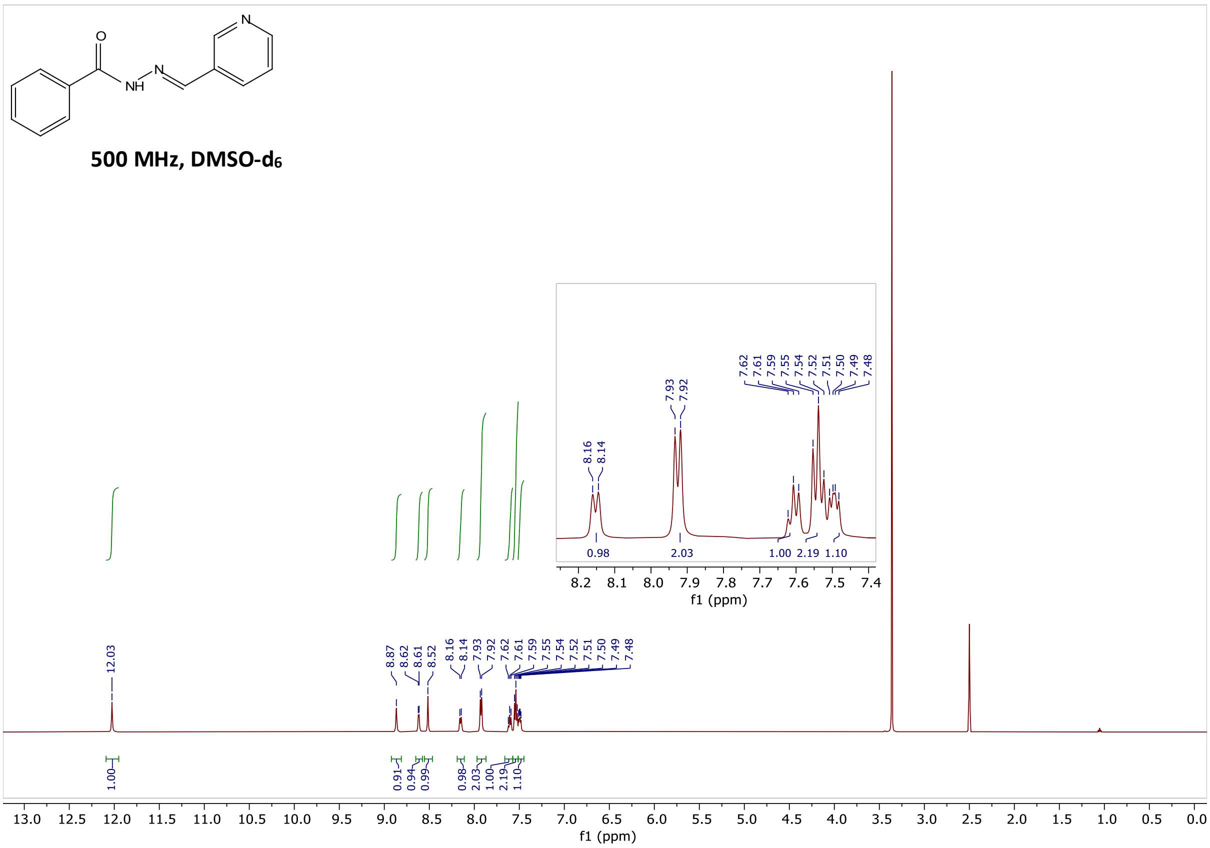

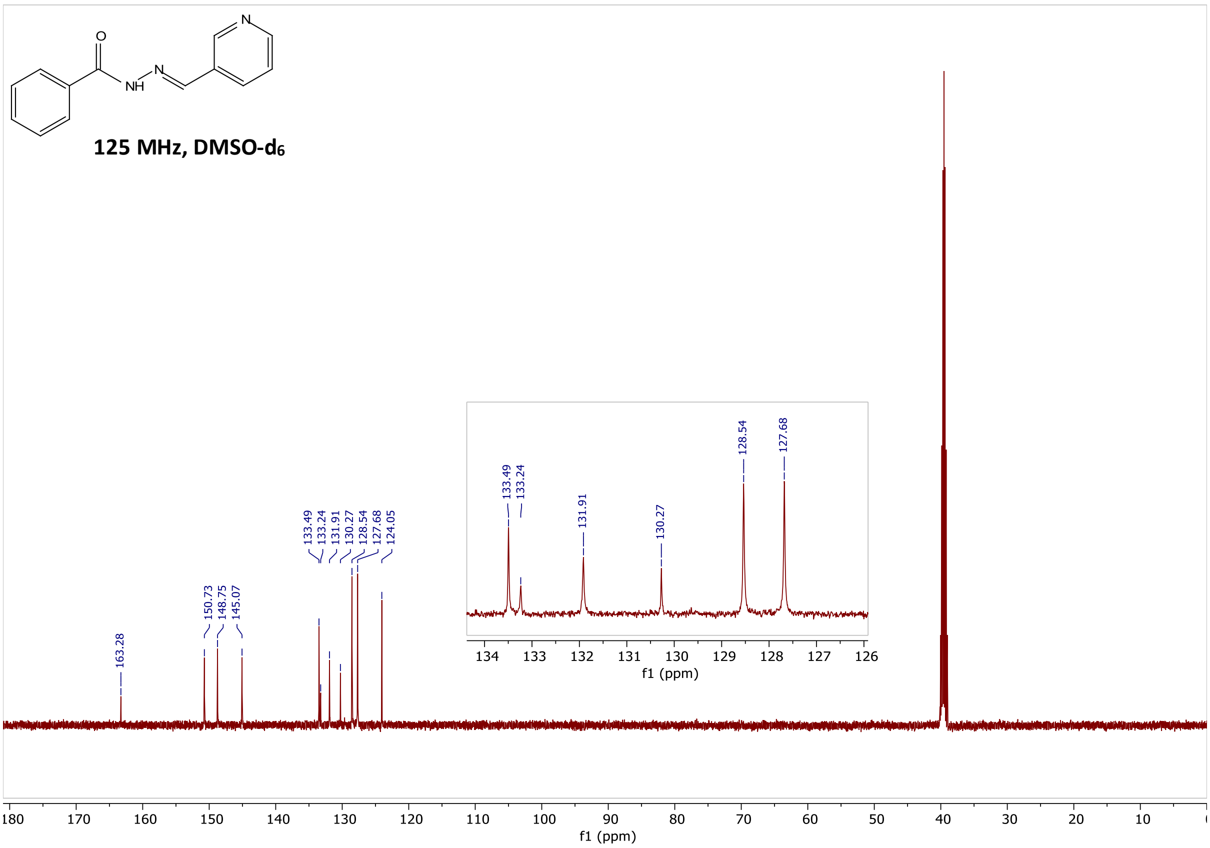

***N'*-(4-Methoxybenzylidene)-4-(trifluoromethyl)benzohydrazide, 1e, 500 MHz, DMSO-*d*$_6$:**

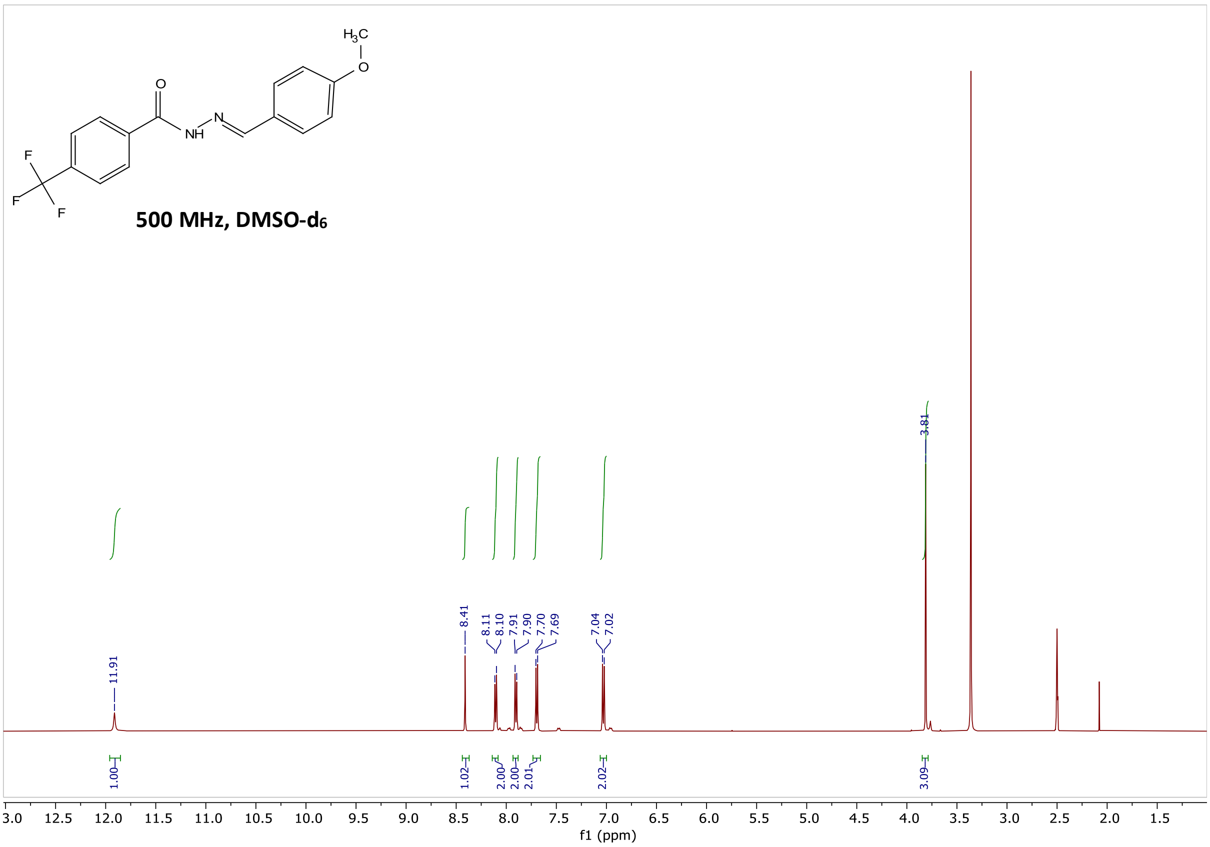

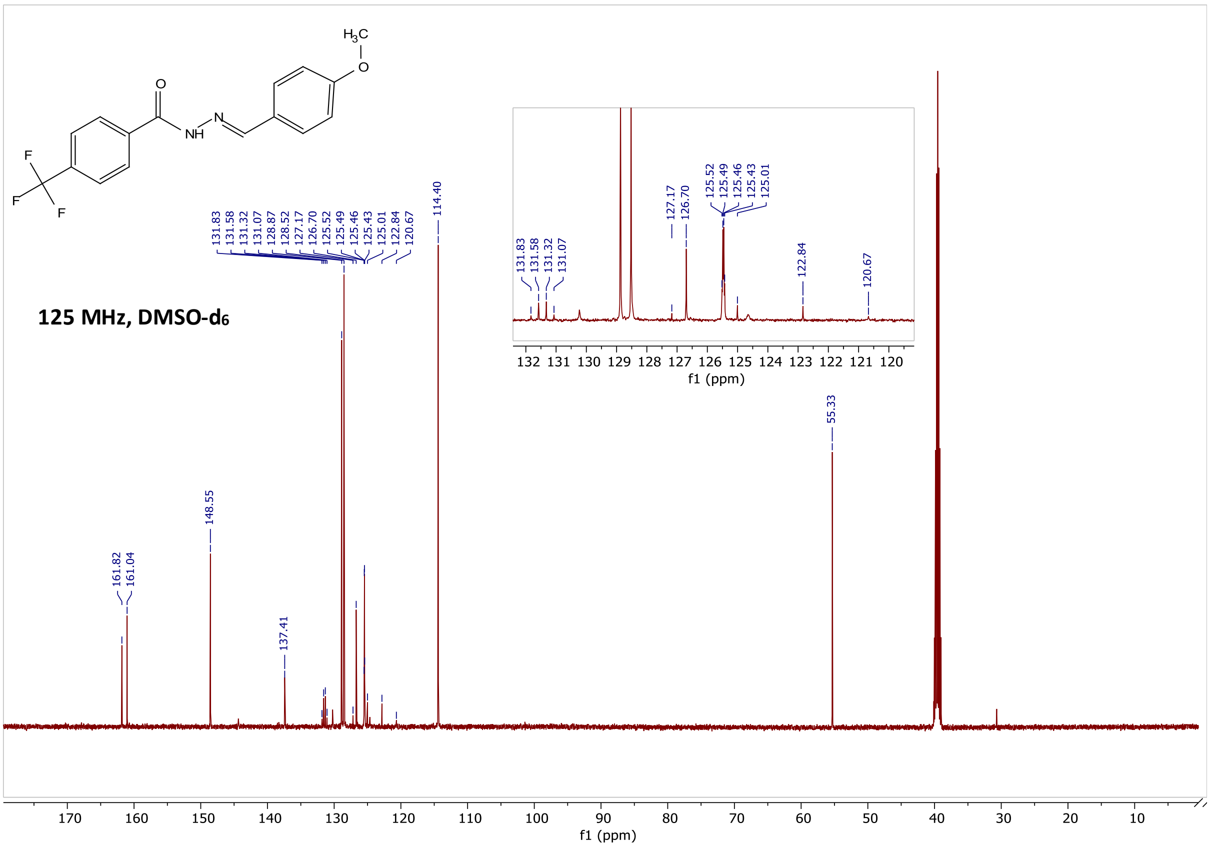

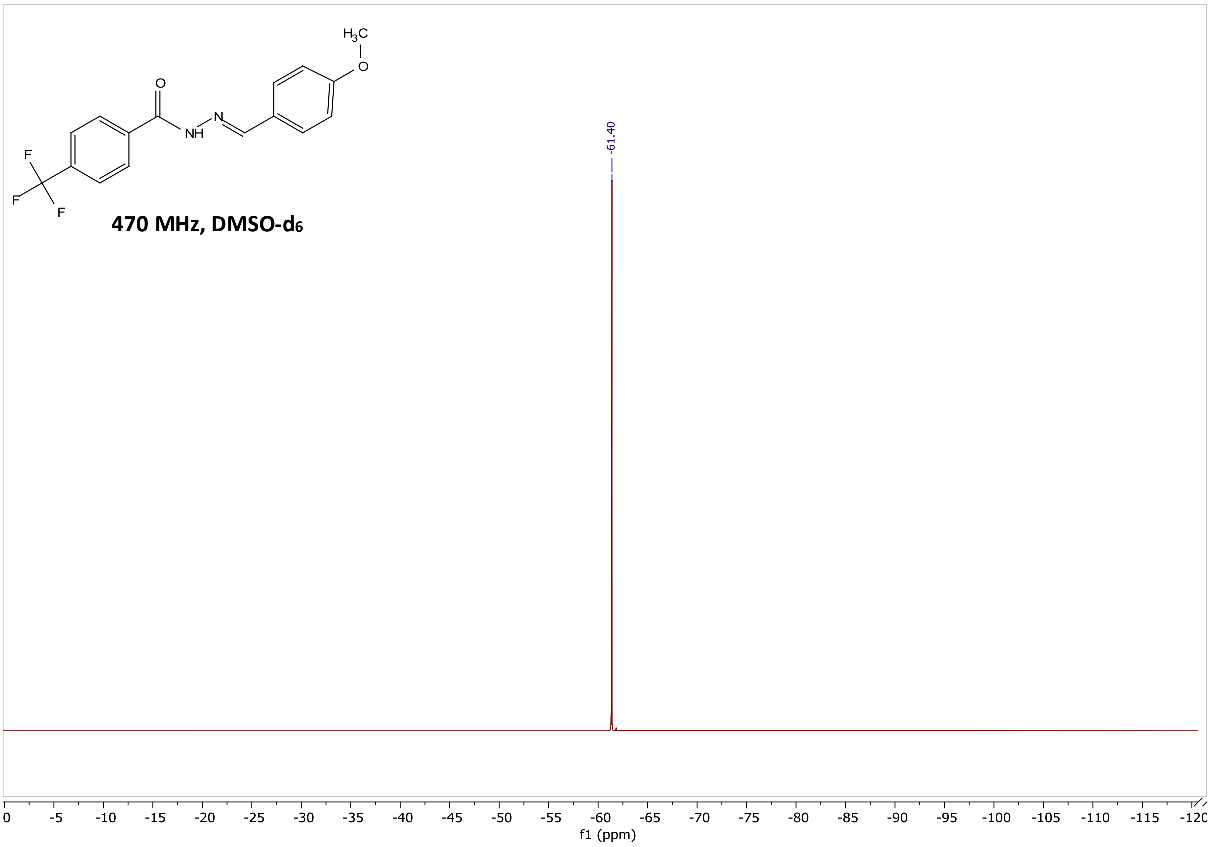

N'-(4-Methoxybenzylidene)benzohydrazide, 1f, 400 MHz, DMSO-d_6:

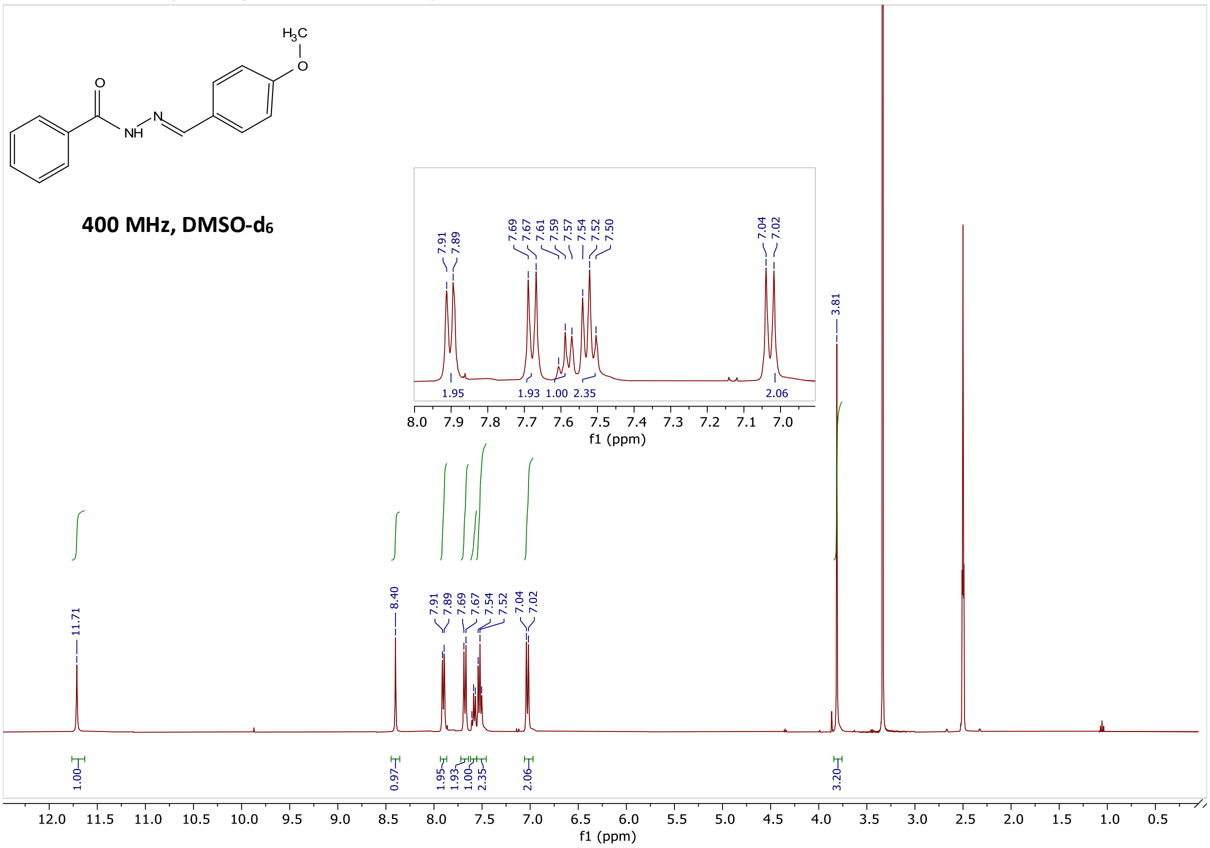

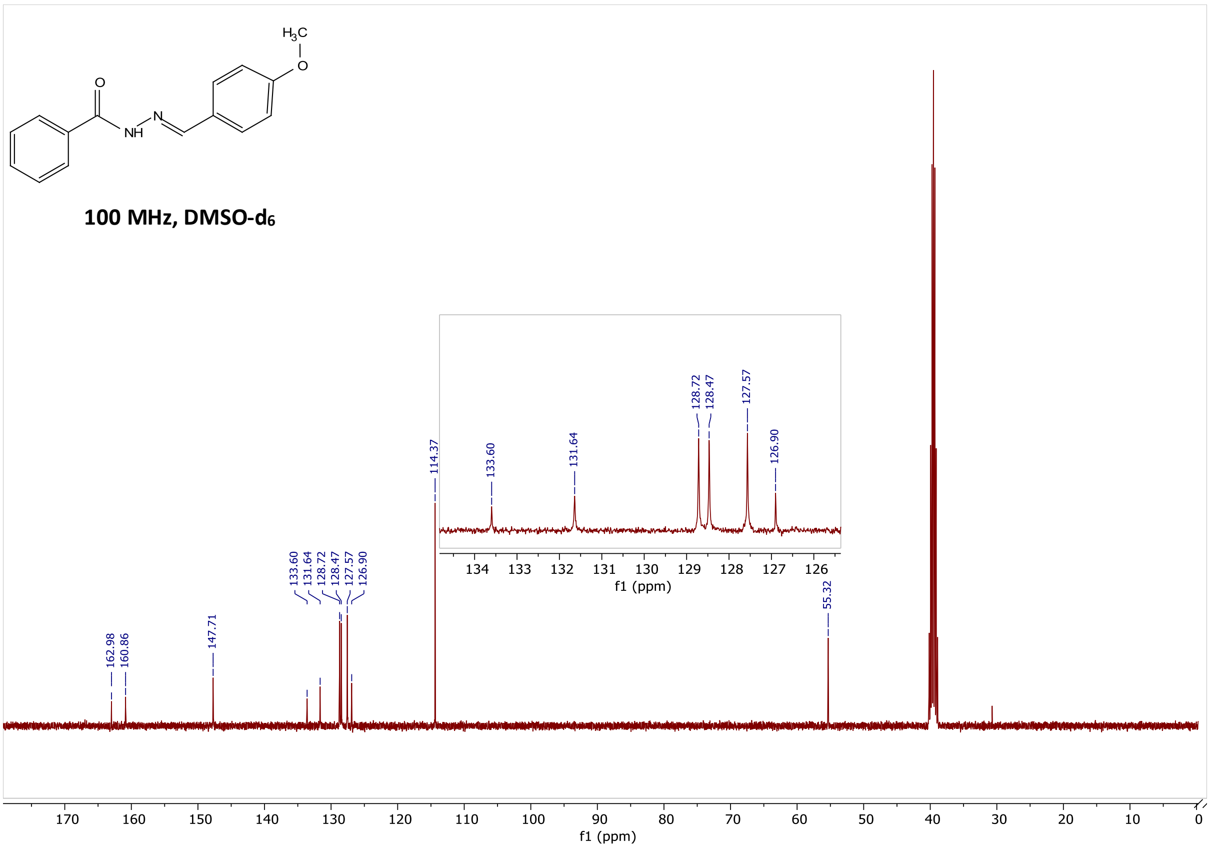

N'-(2-Nitrobenzylidene)-4-(trifluoromethyl)benzohydrazide, 1g, 400 MHz, DMSO-d_6:

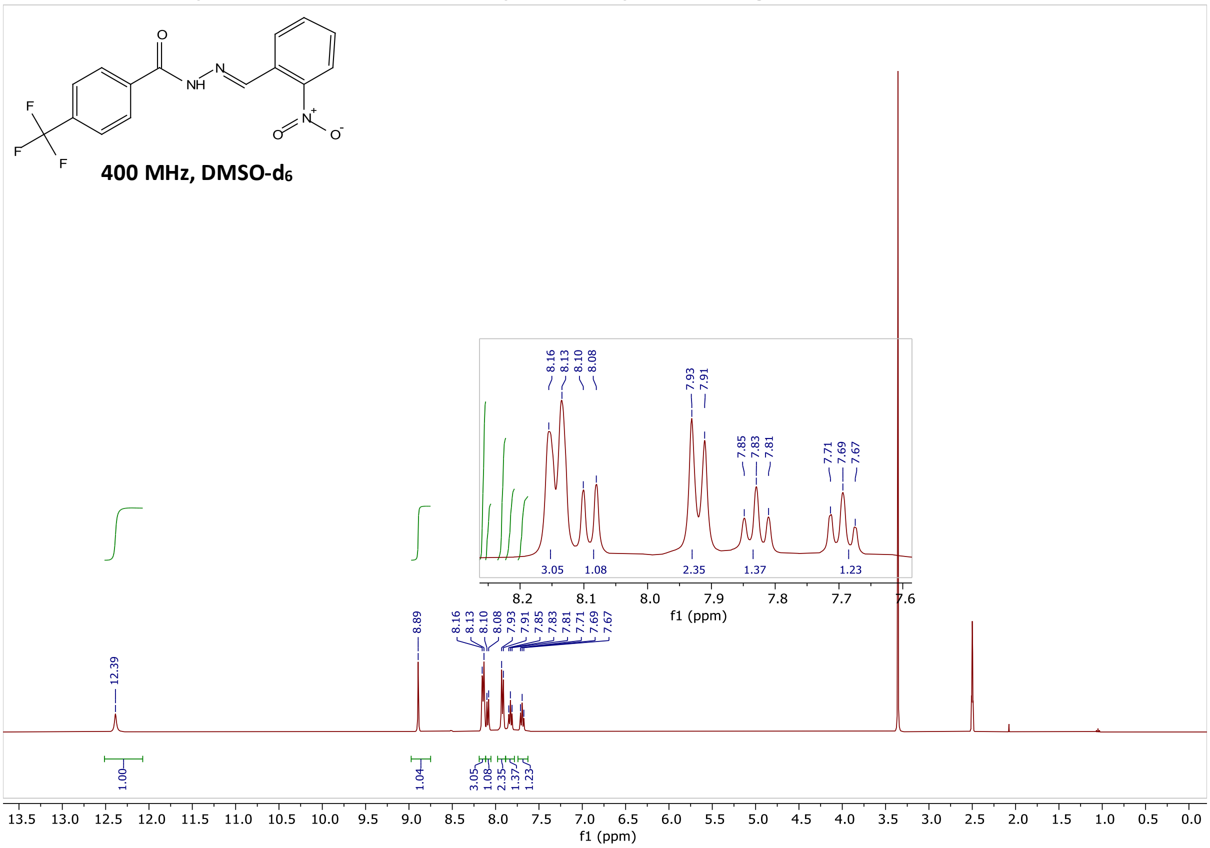

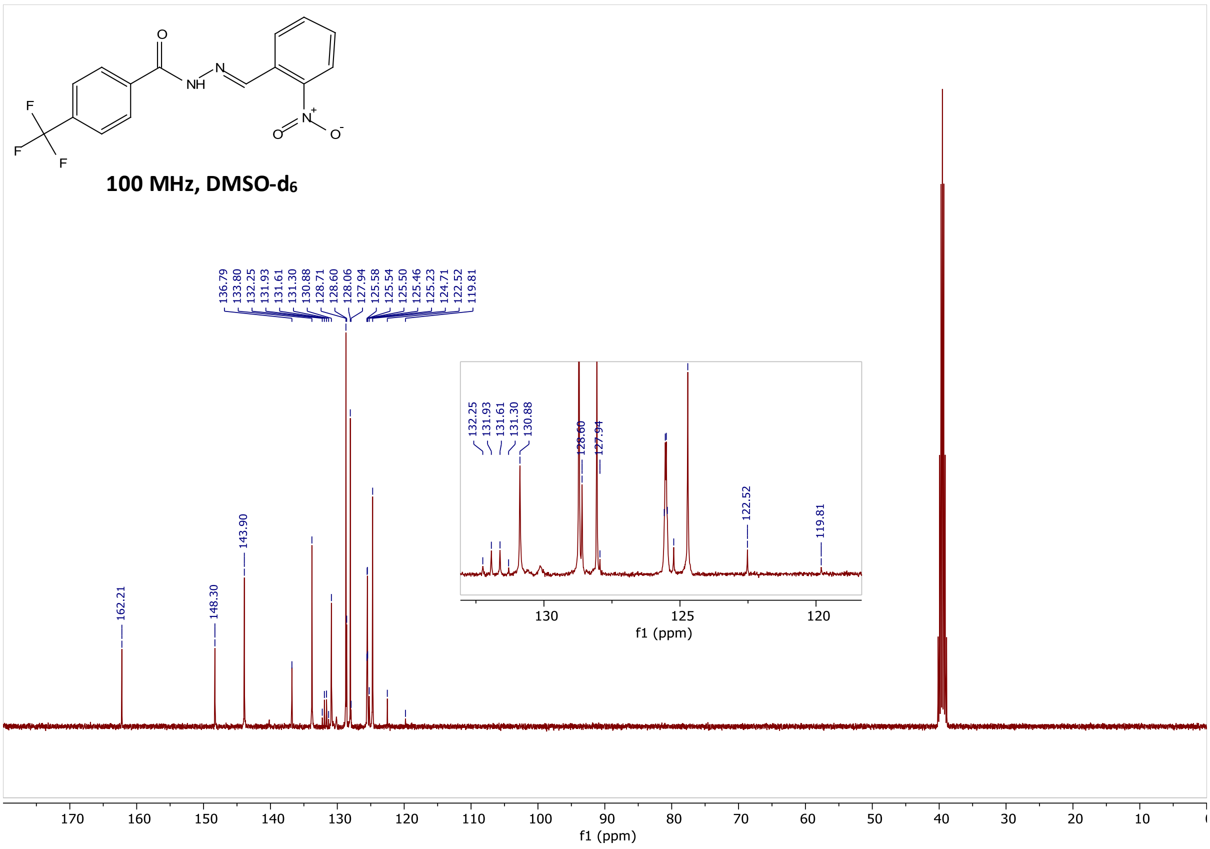
100 MHz, DMSO-d6
136.79
133.80
132.25
131.93
131.61
131.30
130.88
128.71
128.60
128.06
127.94
125.58
125.54
125.50
125.46
125.23
124.71
122.52
119.81
162.21
148.30
143.90
f1 (ppm)

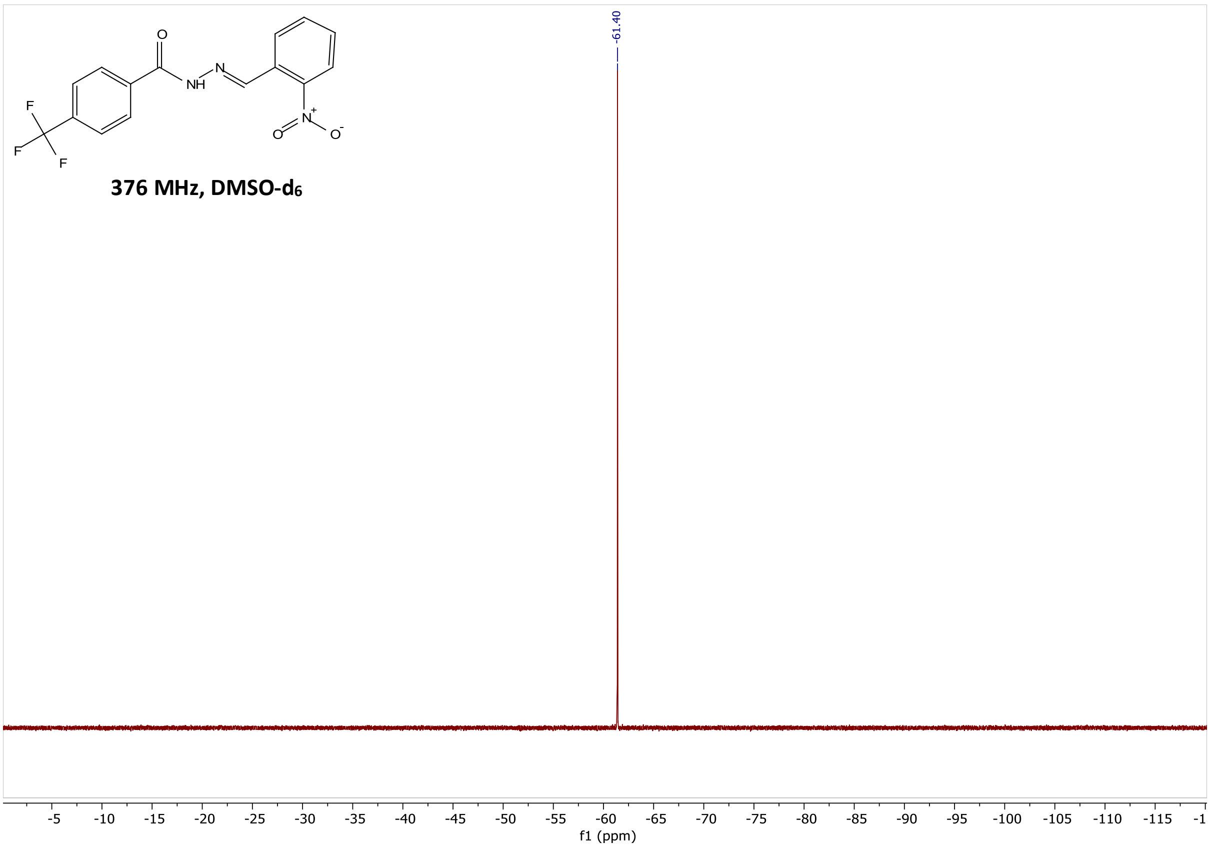
376 MHz, DMSO-d6
-61.40
f1 (ppm)

***N'*-(2-Nitrobenzylidene)benzohydrazide, 1h, 400 MHz, DMSO-*d*$_6$:**

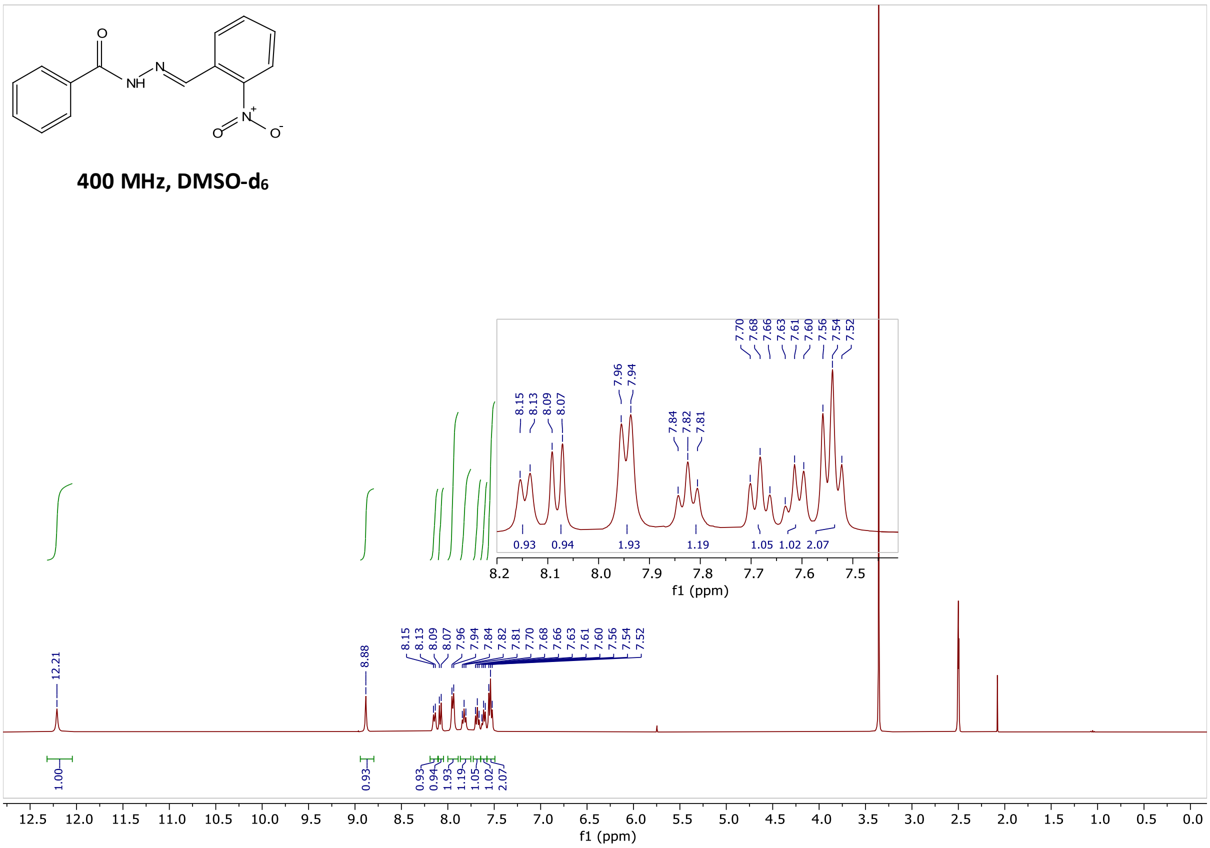

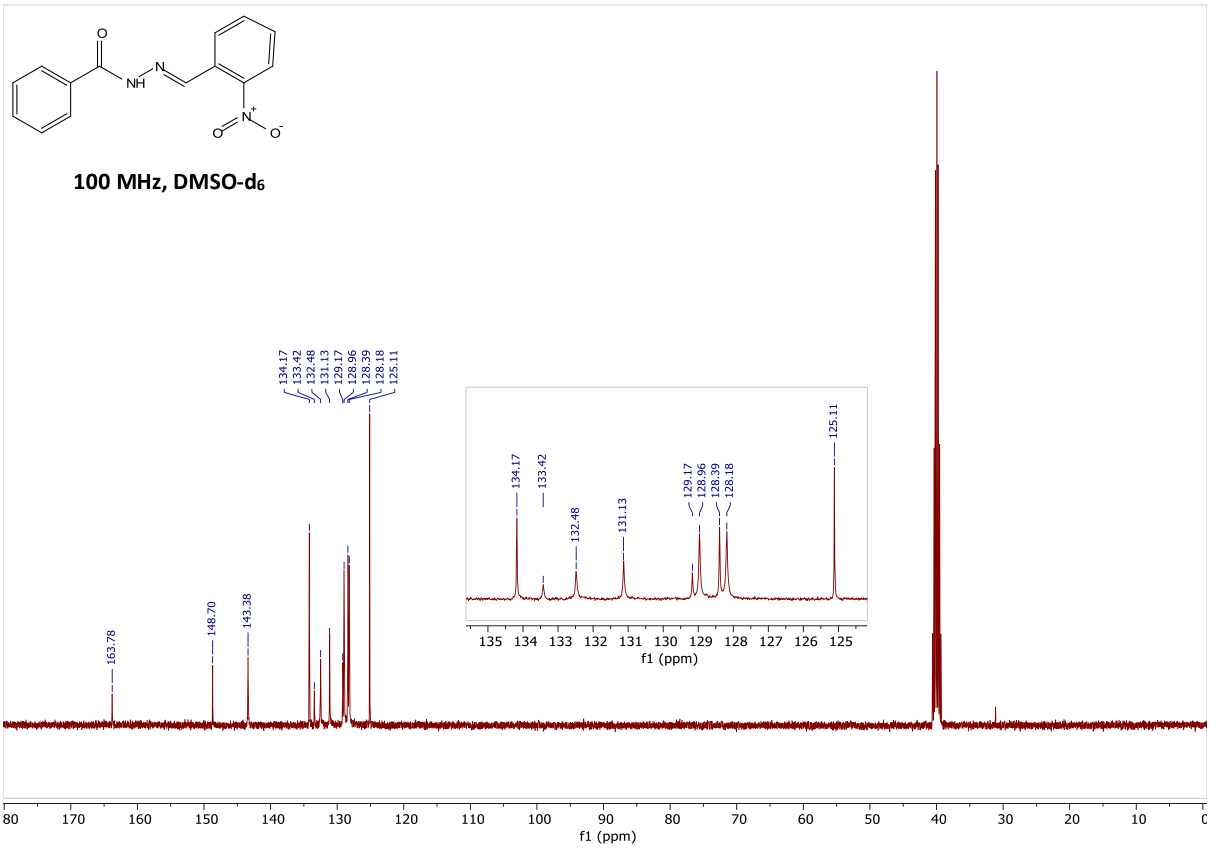

***N'*-((1*E*,2*E*)-3-Phenylallylidene)-4-(trifluoromethyl)benzohydrazide, 1i, 500 MHz, DMSO-*d*$_6$:**

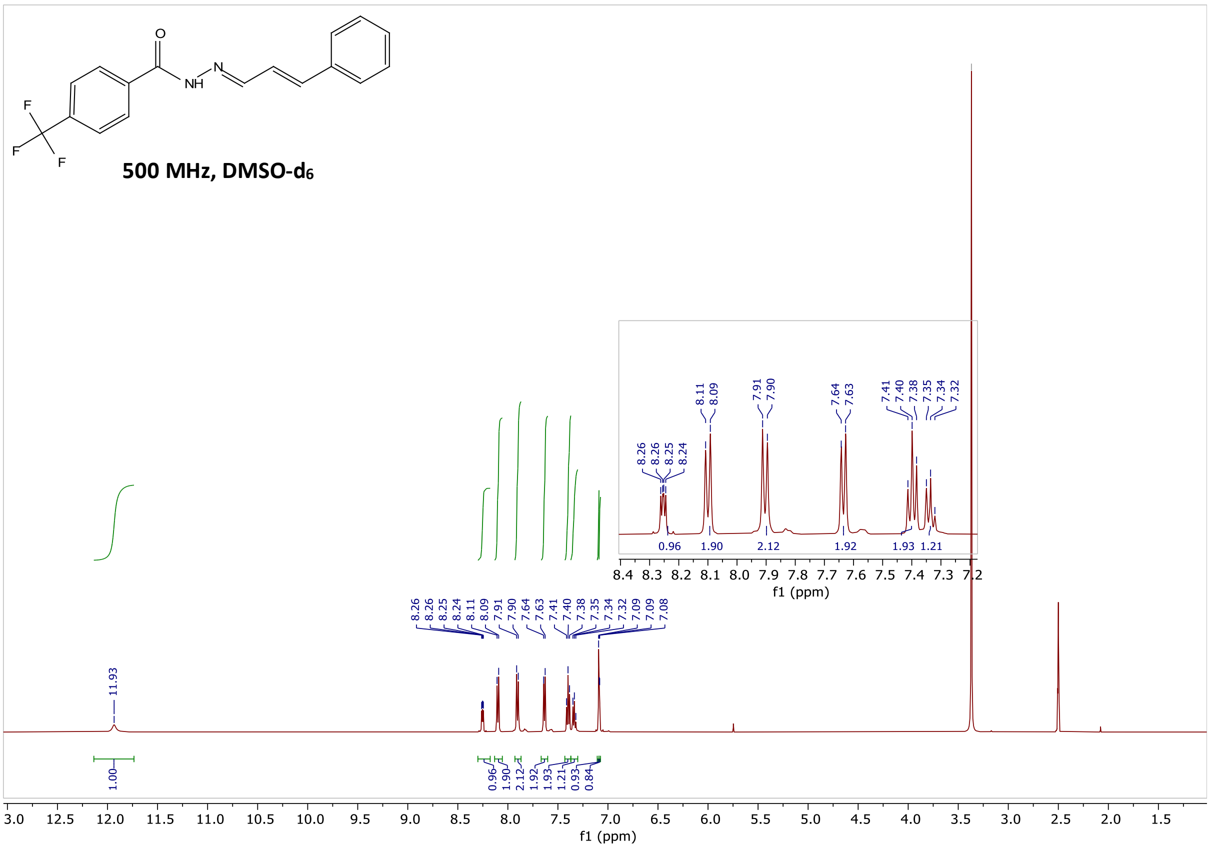

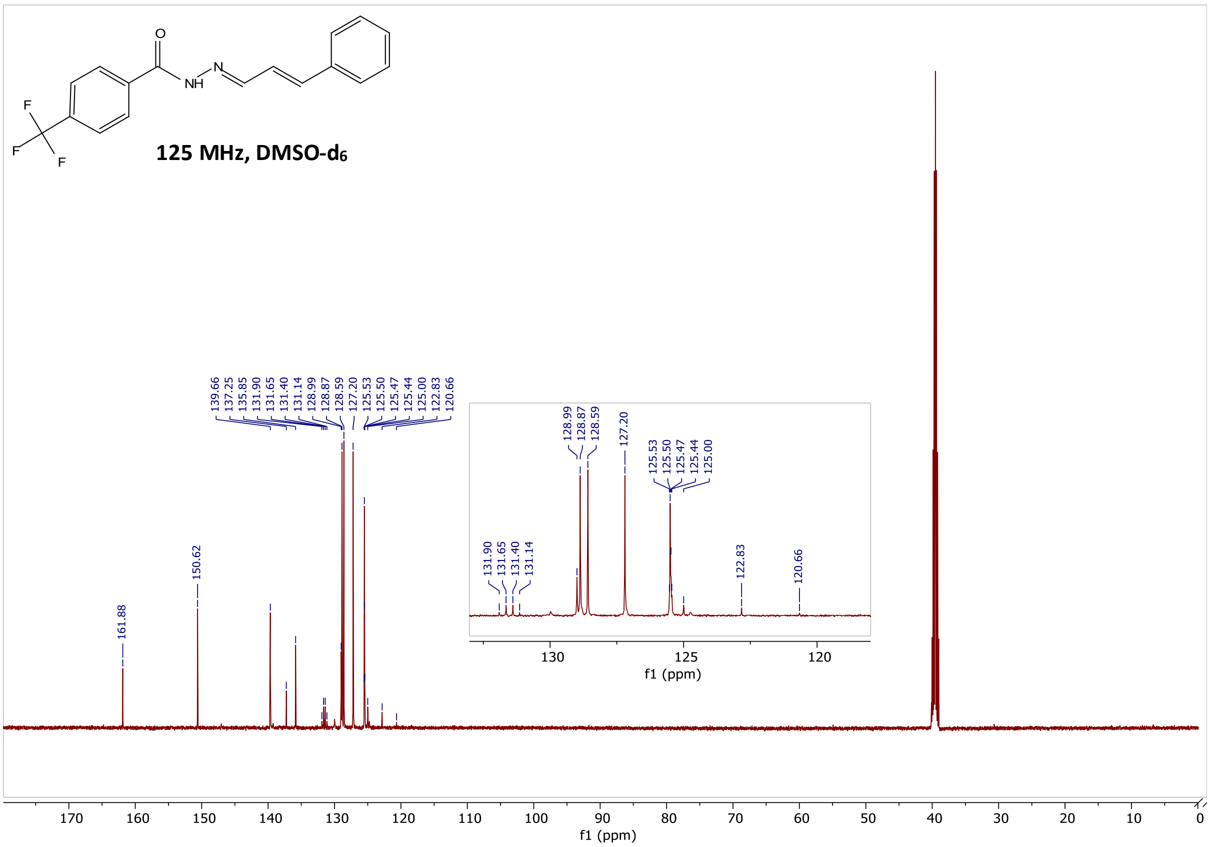

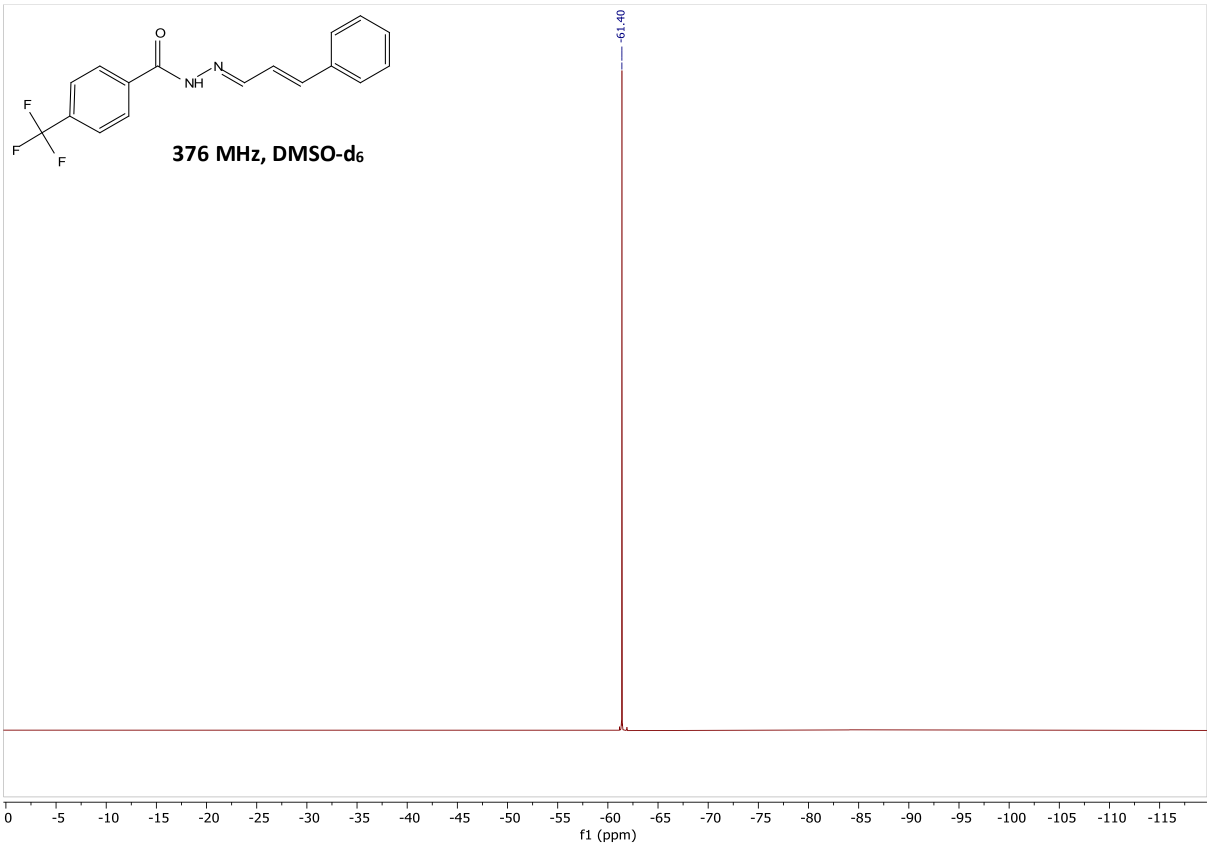

N'-((1*E*,2*E*)-3-Phenylallylidene)benzohydrazide, 1j, 500 MHz, DMSO-d_6:

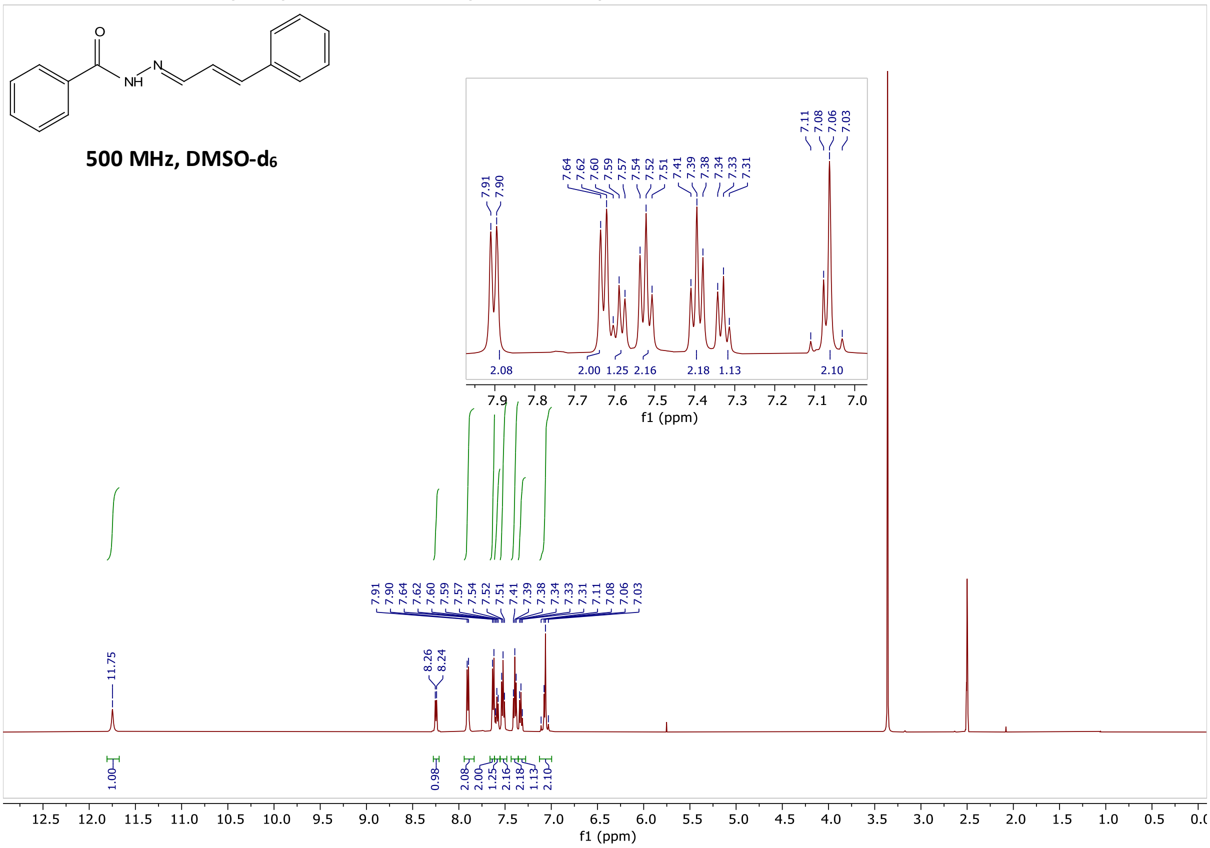

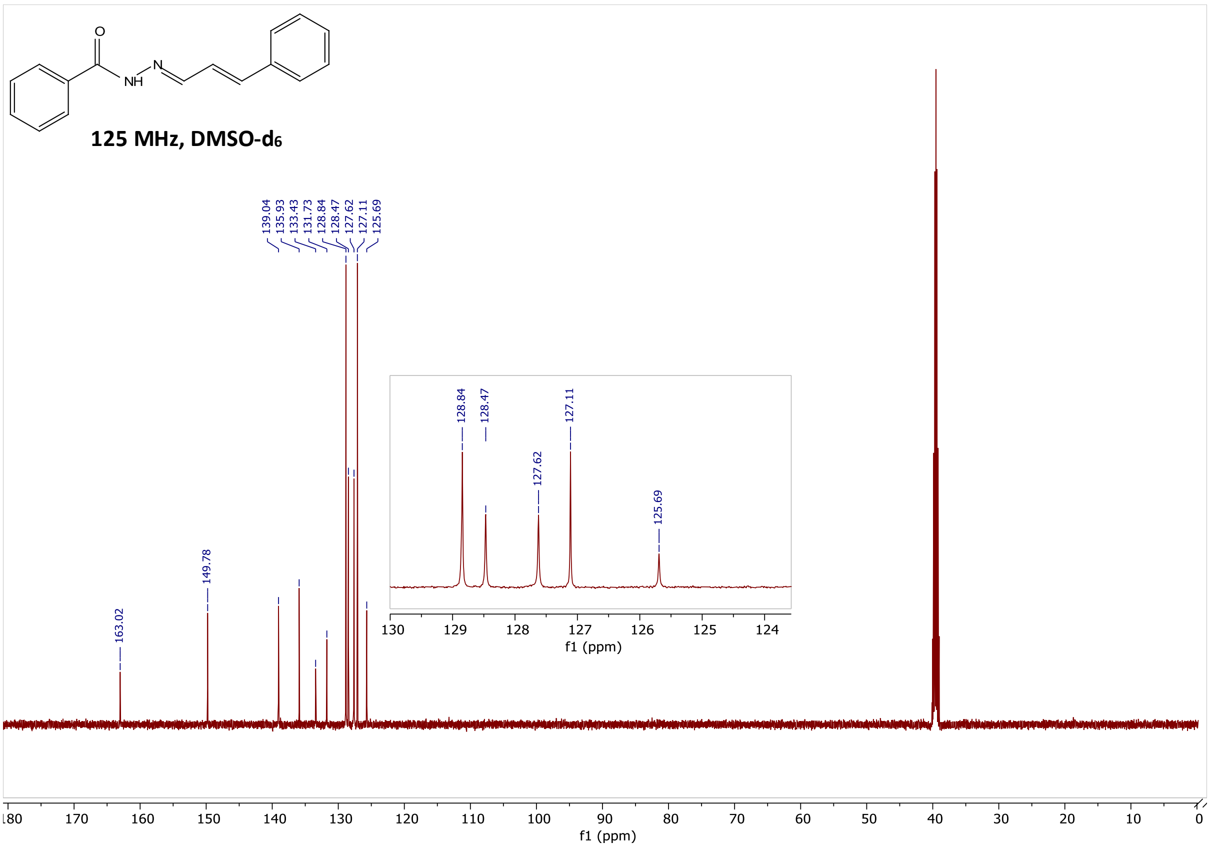

5-(4-Bromophenyl)-*N'*-(thiophen-2-ylmethylene)isoxazole-3-carbohydrazide, 1m, 500 MHz, DMSO-*d*$_6$:

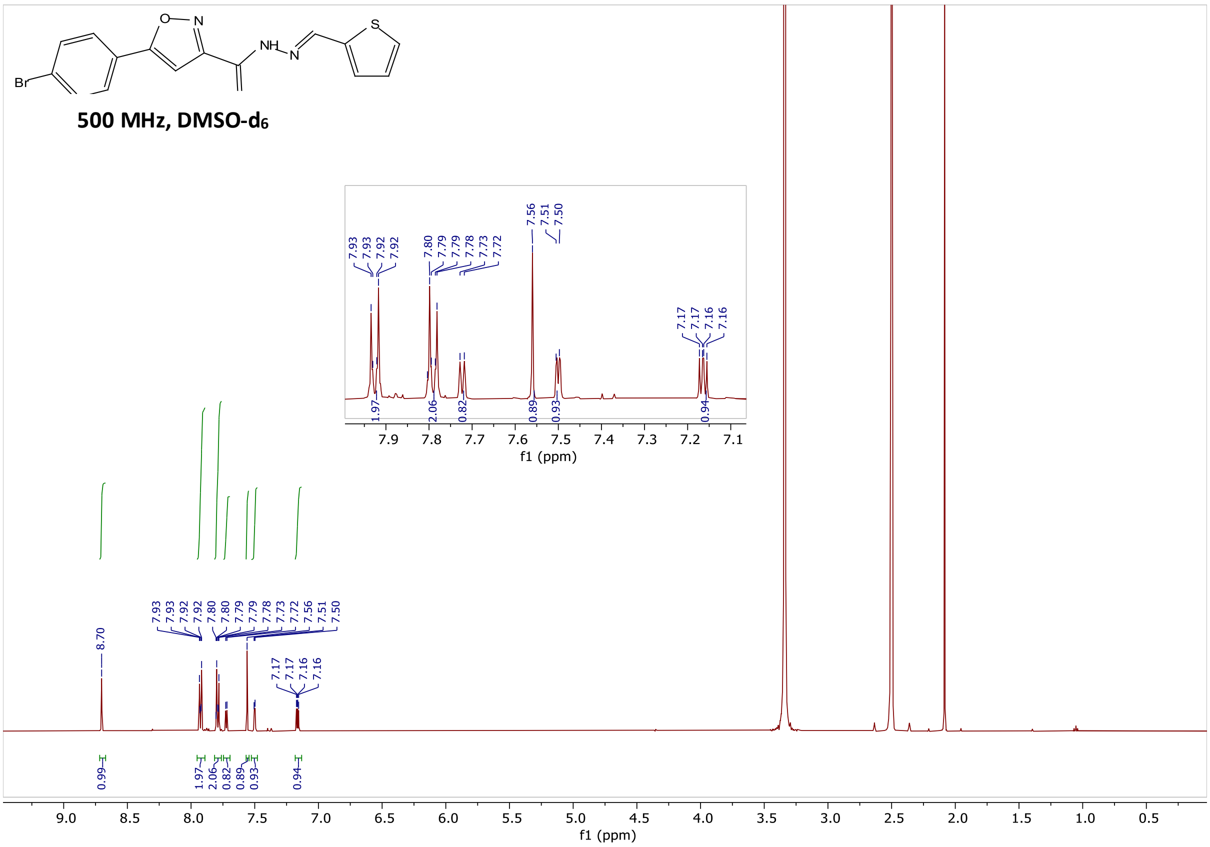

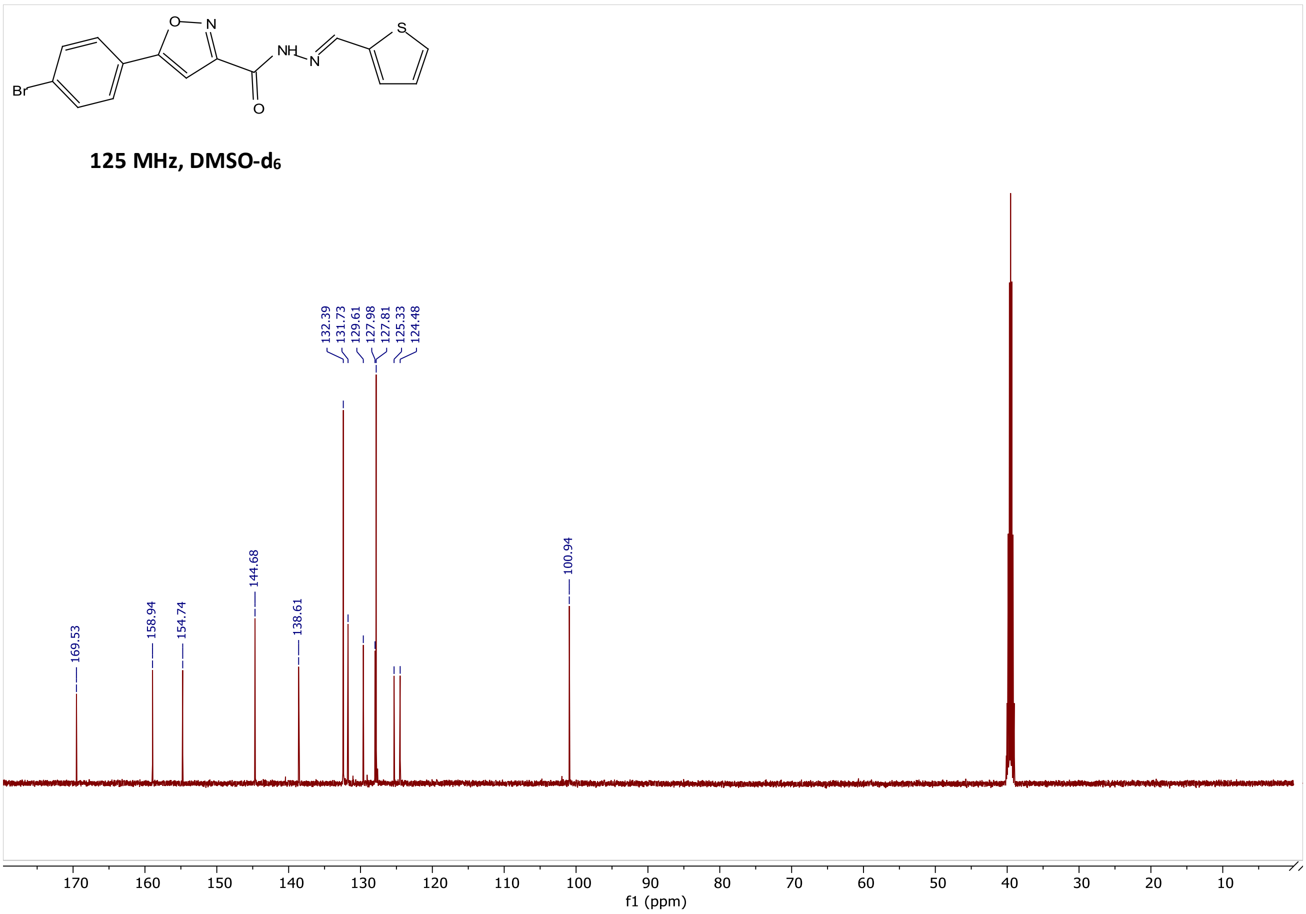

2-(Thiophen-2-yl)-5-(4-(trifluoromethyl)phenyl)-1,3,4-oxadiazole, 2a, 400 MHz, $CDCl_3$:

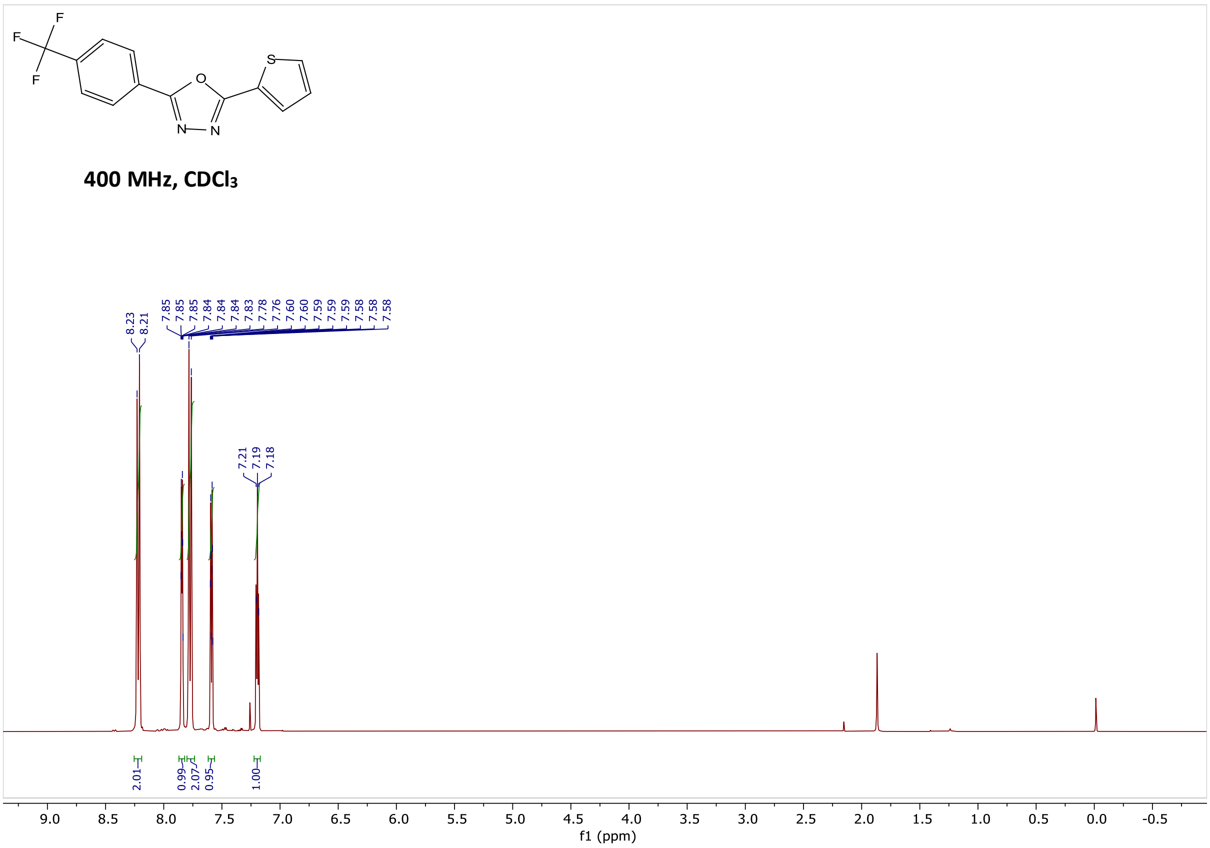

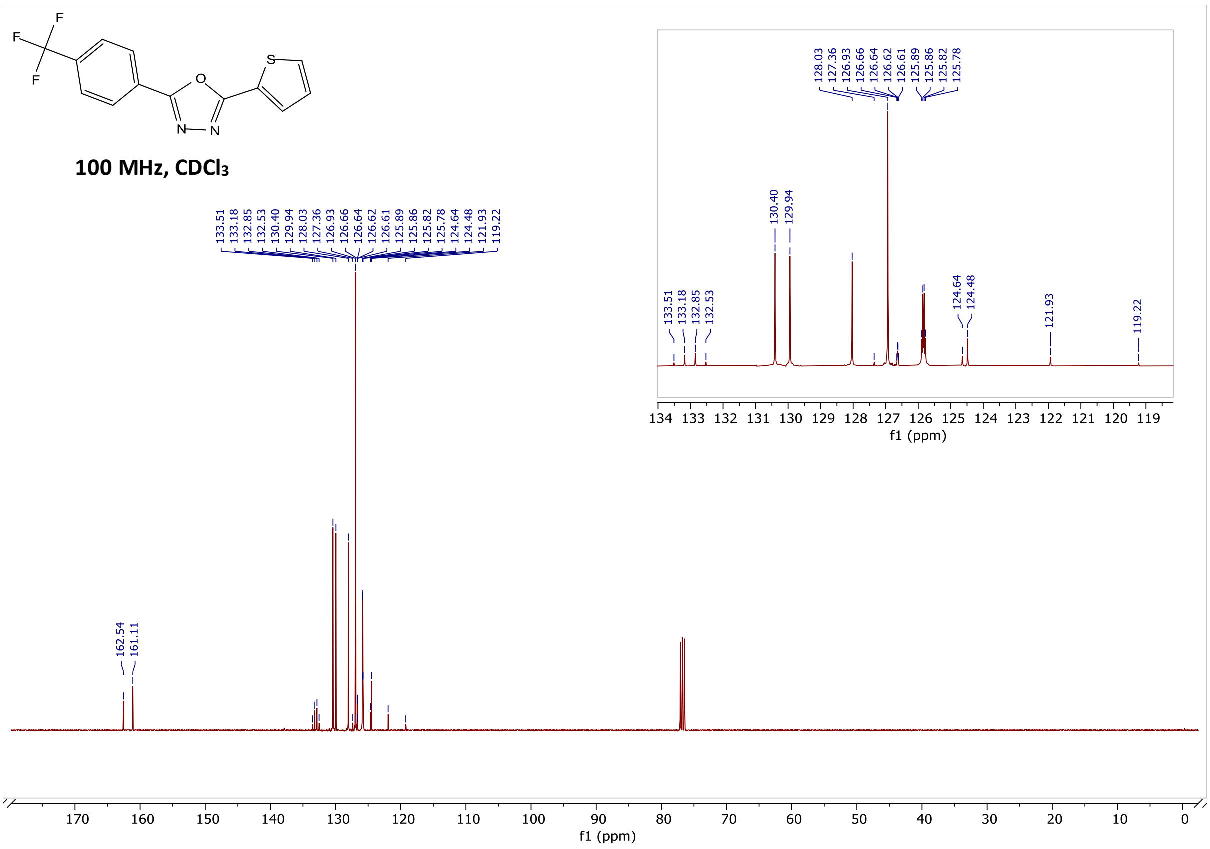
100 MHz, CDCl3
162.54
161.11
133.51
133.18
132.85
132.53
130.40
129.94
128.03
127.36
126.93
126.66
126.64
126.62
126.61
125.89
125.86
125.82
125.78
124.64
124.48
121.93
119.22
f1 (ppm)

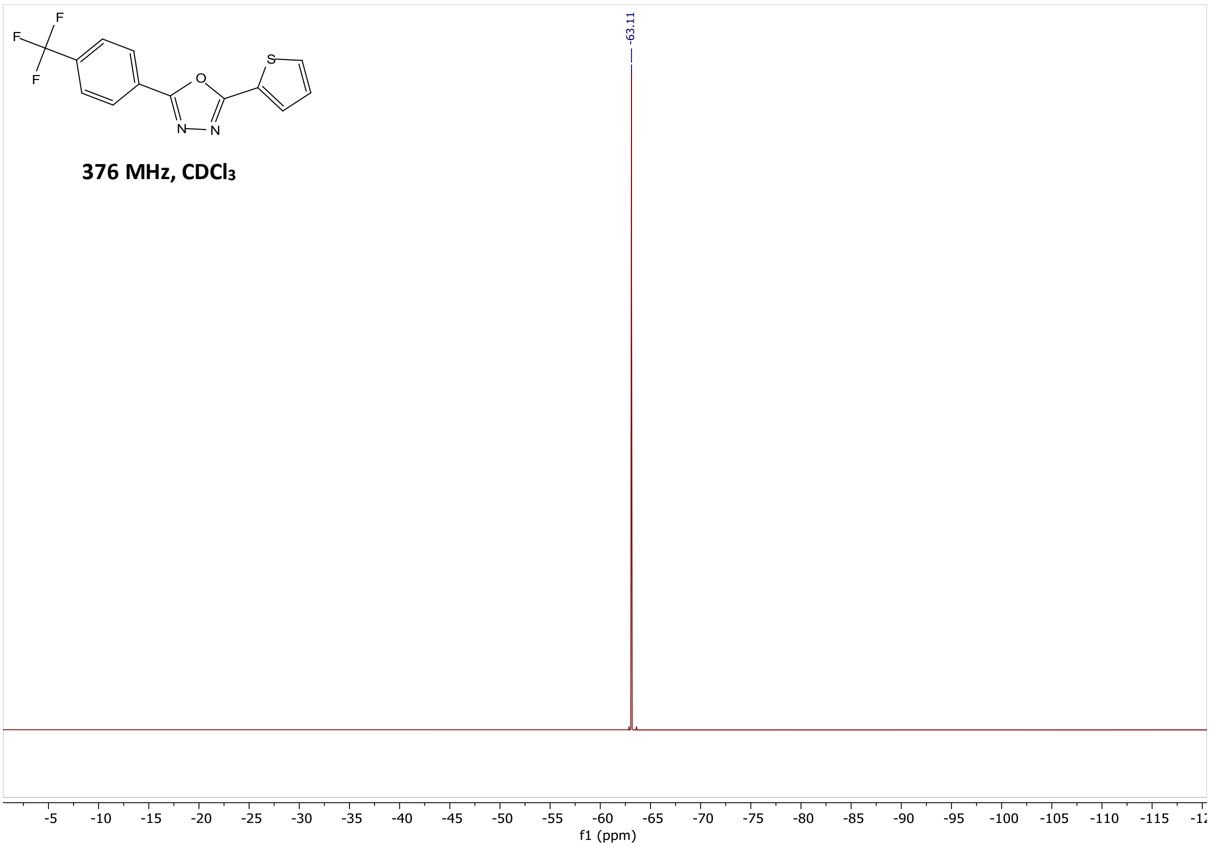
376 MHz, CDCl3
-63.11
f1 (ppm)

Phenyl-5-(thiophen-2-yl)-1,3,4-oxadiazole, 2b, 400 MHz, CDCl$_3$:

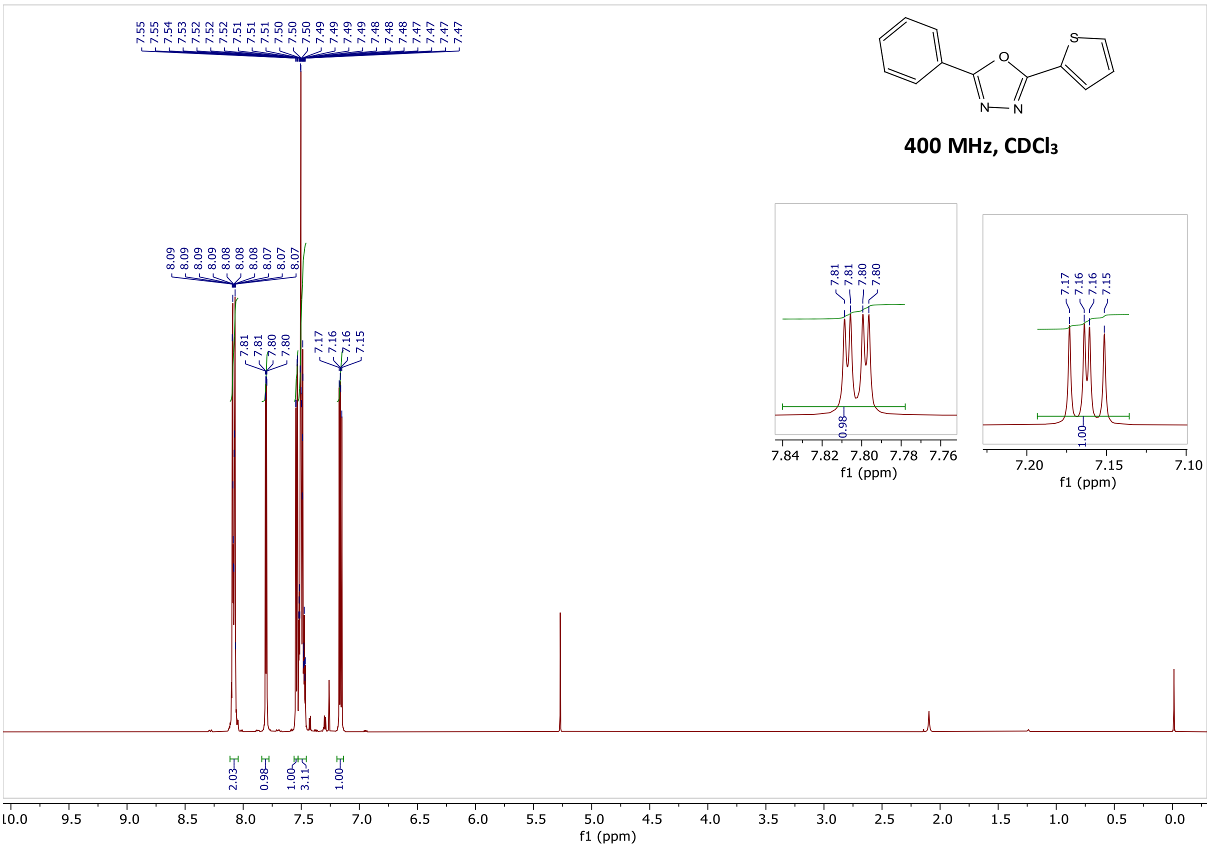

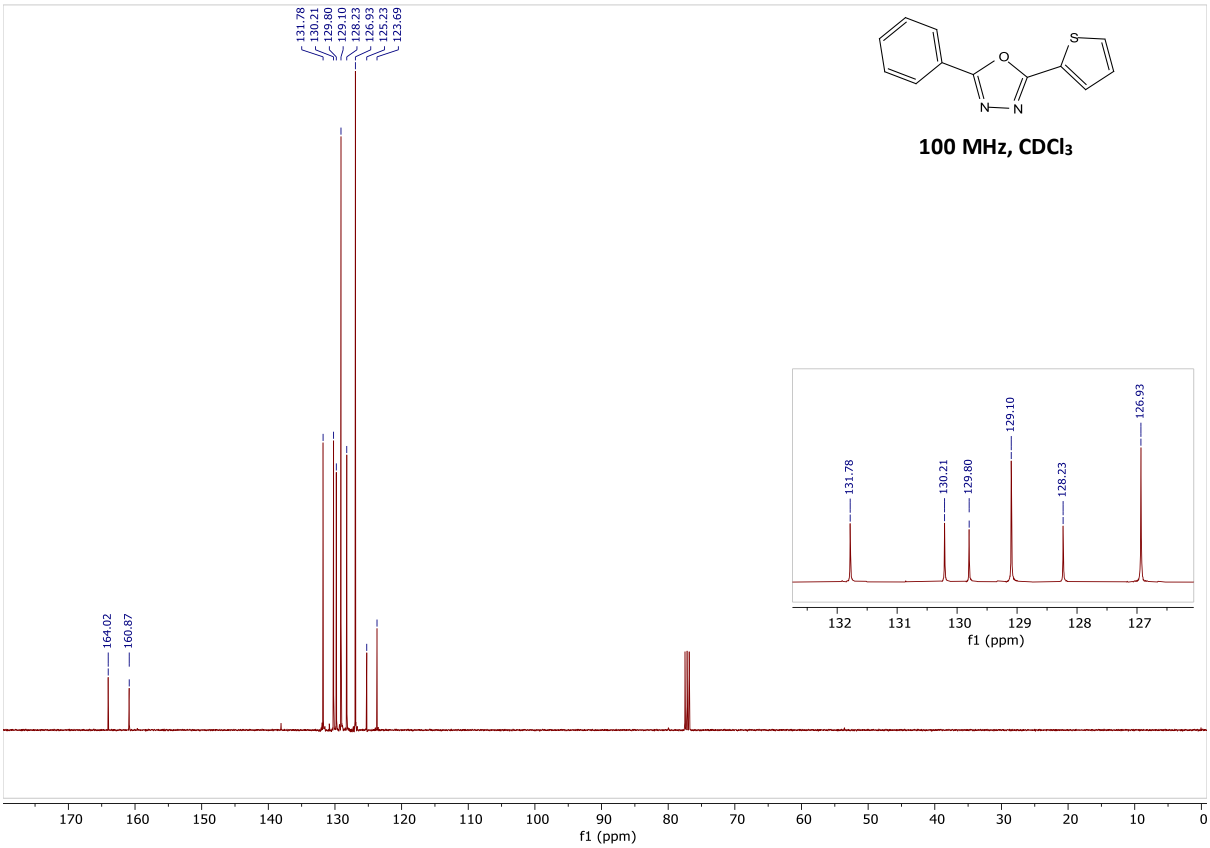

2-(Pyridin-3-yl)-5-(4-(trifluoromethyl)phenyl)-1,3,4-oxadiazole, 2c, 500 MHz, CDCl$_3$:

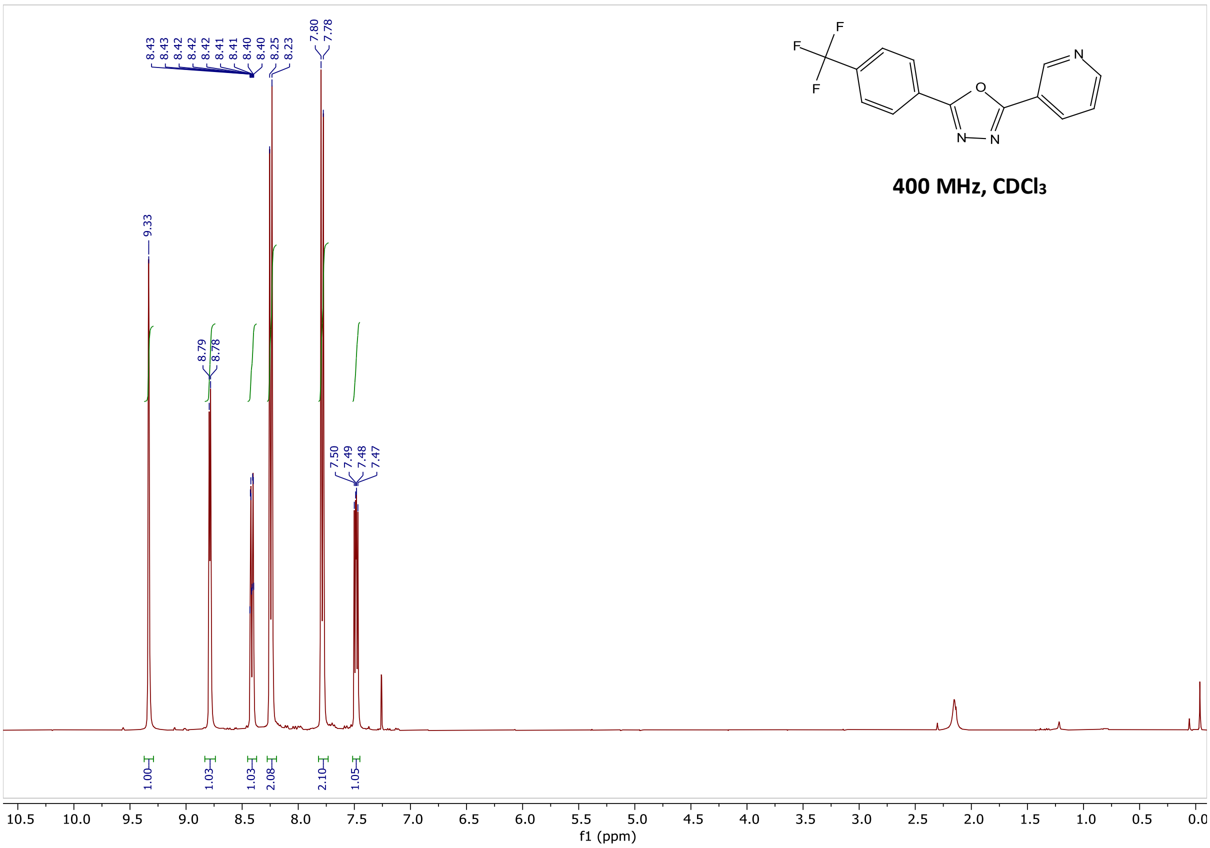

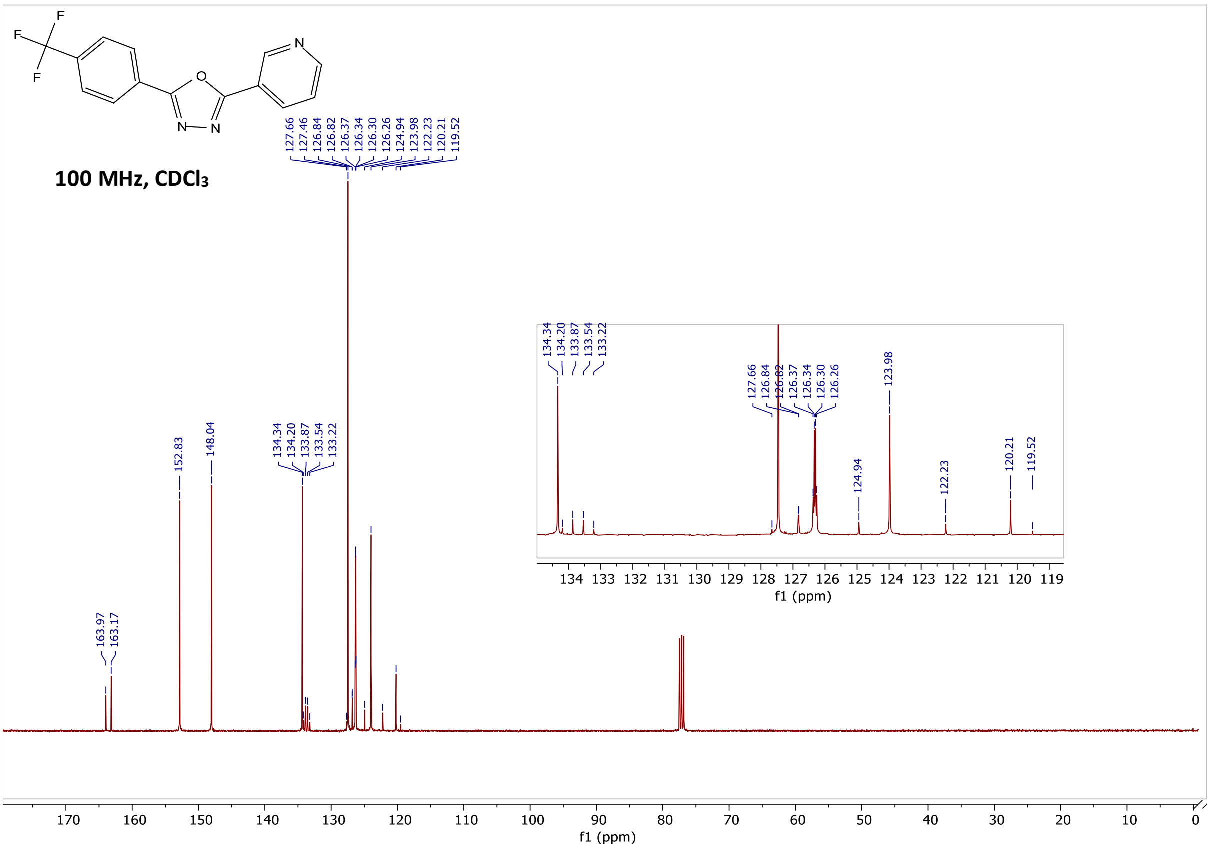

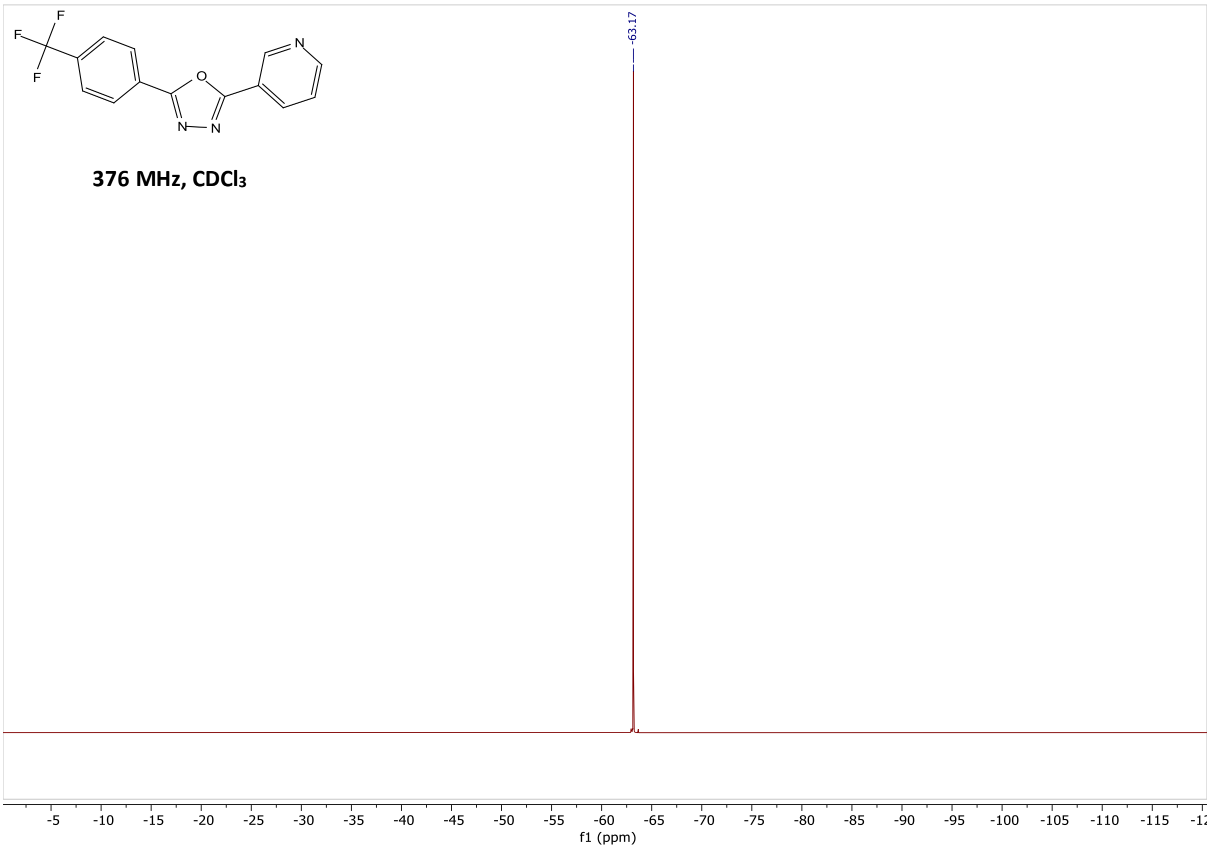

2-Phenyl-5-(pyridine-3-yl)-1,3,4-oxadiazole, 2d, 500 MHz, $CDCl_3$:

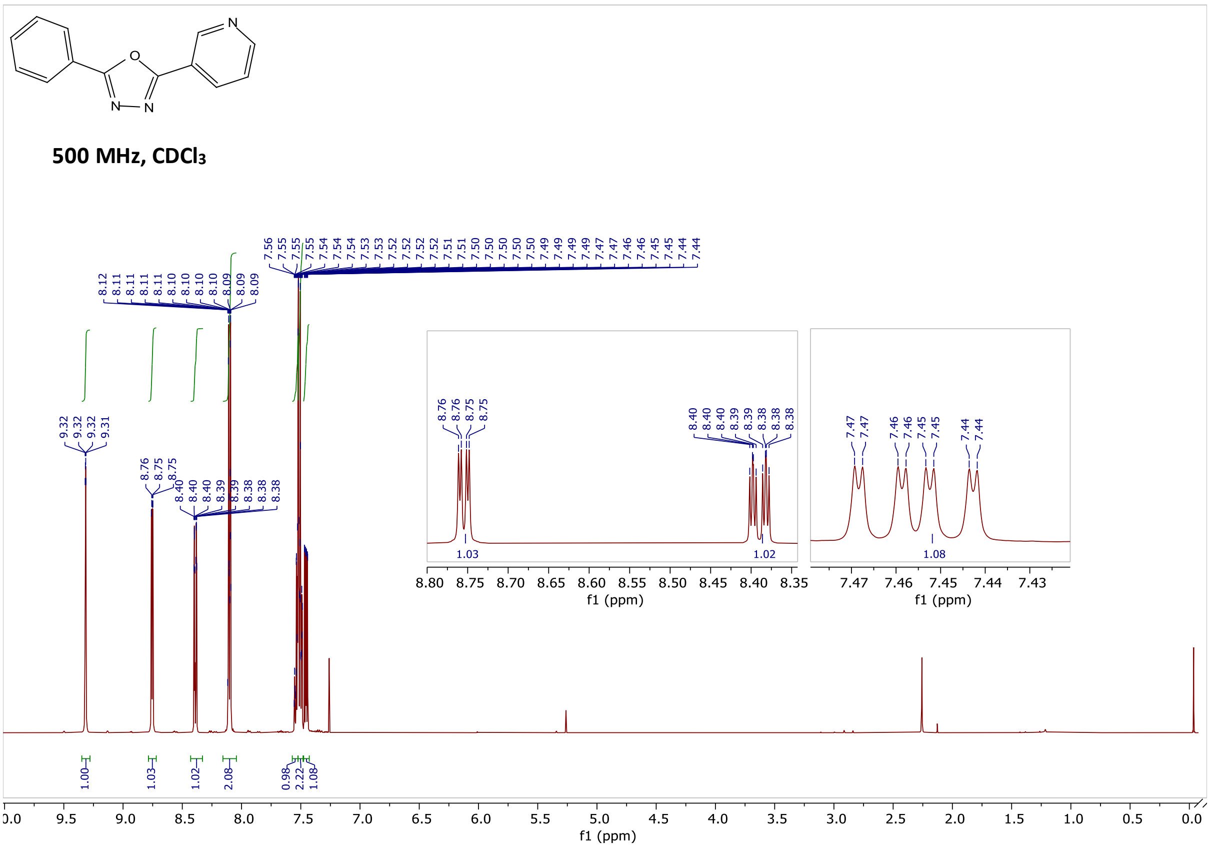

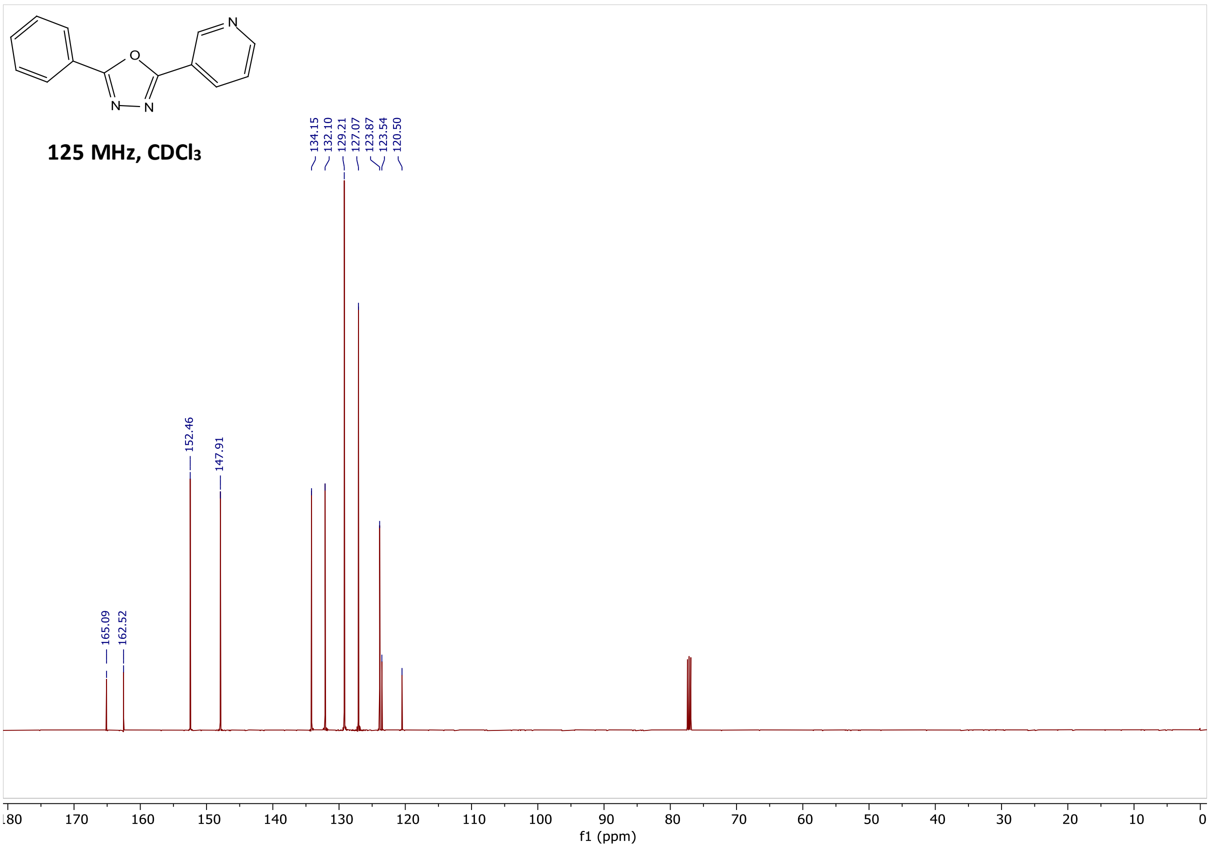

2-(4-Methoxyphenyl)-5-(4-(trifluoromethyl)phenyl)-1,3,4-oxadiazole, 2e, 500 MHz, $CDCl_3$:

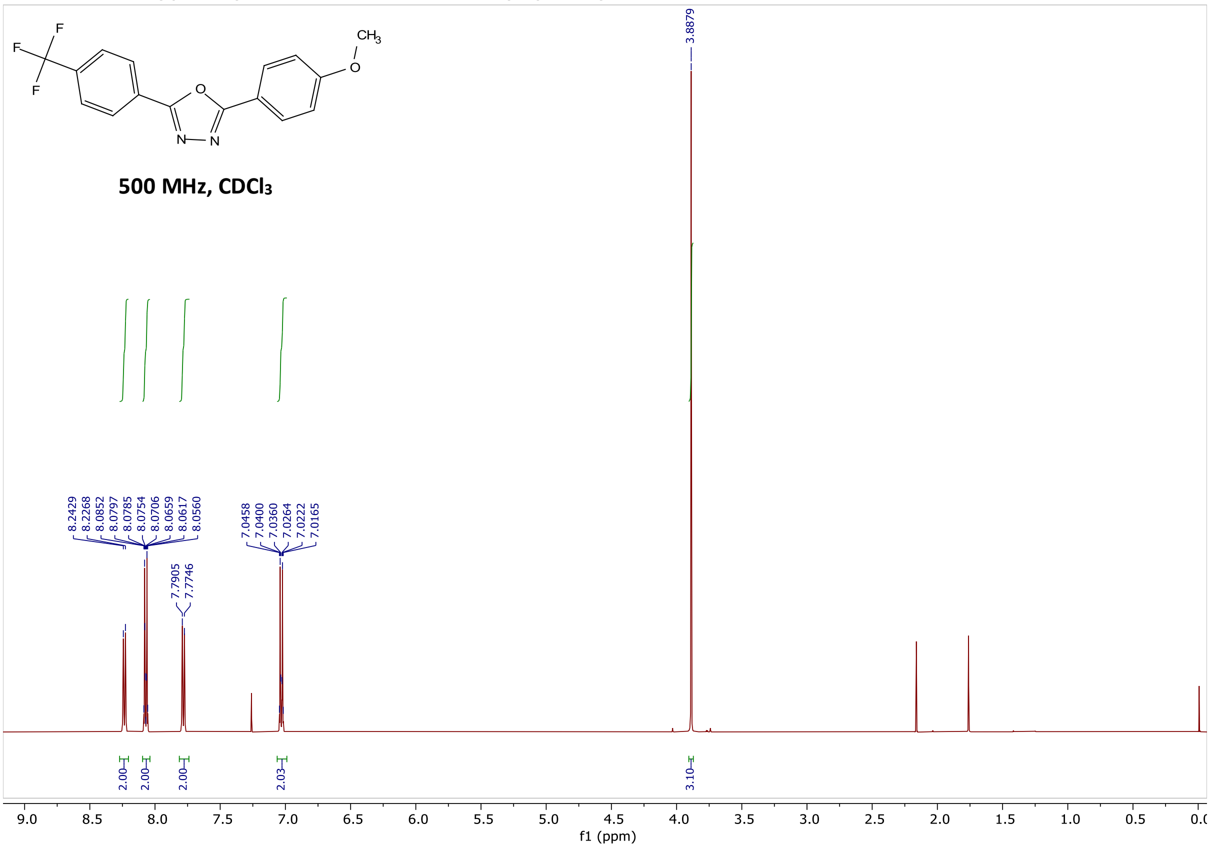

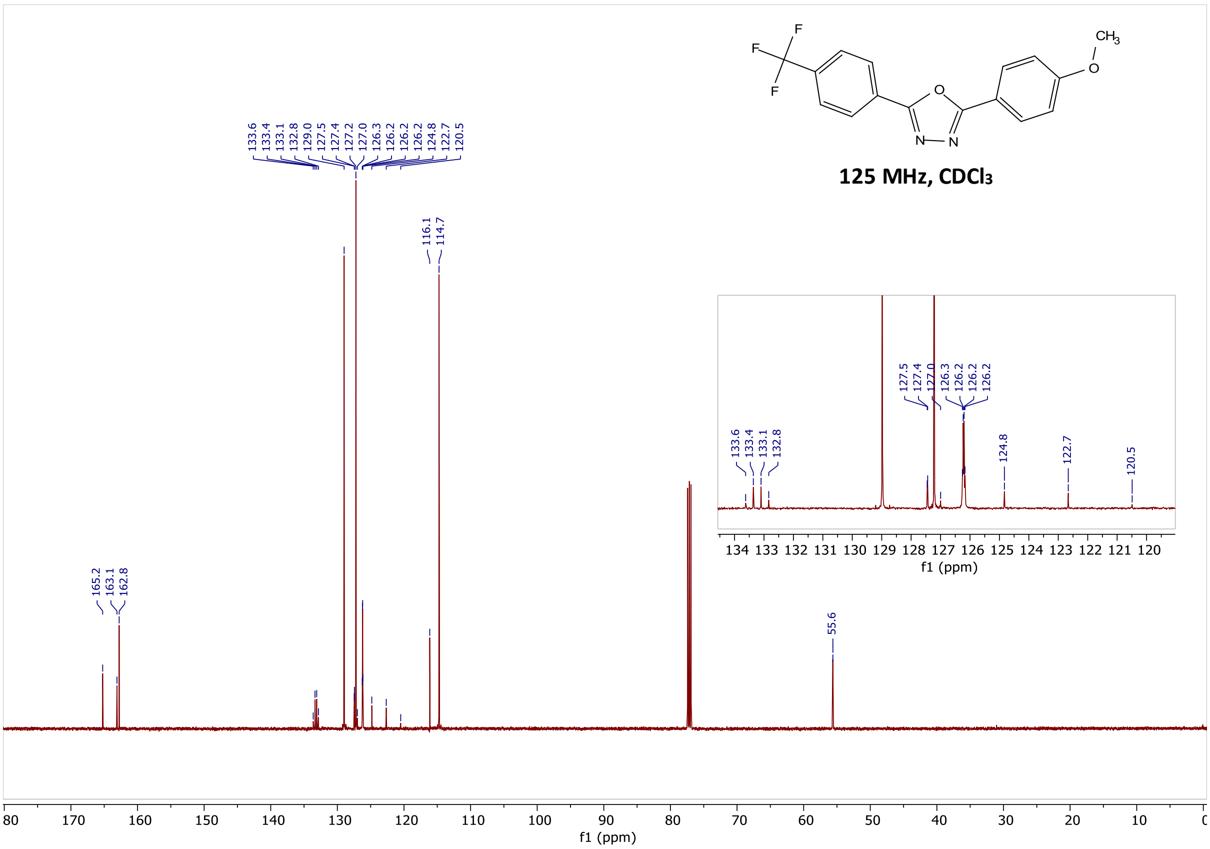
125 MHz, CDCl3
165.2
163.1
162.8
133.6
133.4
133.1
132.8
129.0
127.5
127.4
127.2
127.0
126.3
126.2
126.2
126.2
124.8
122.7
120.5
116.1
114.7
55.6
f1 (ppm)

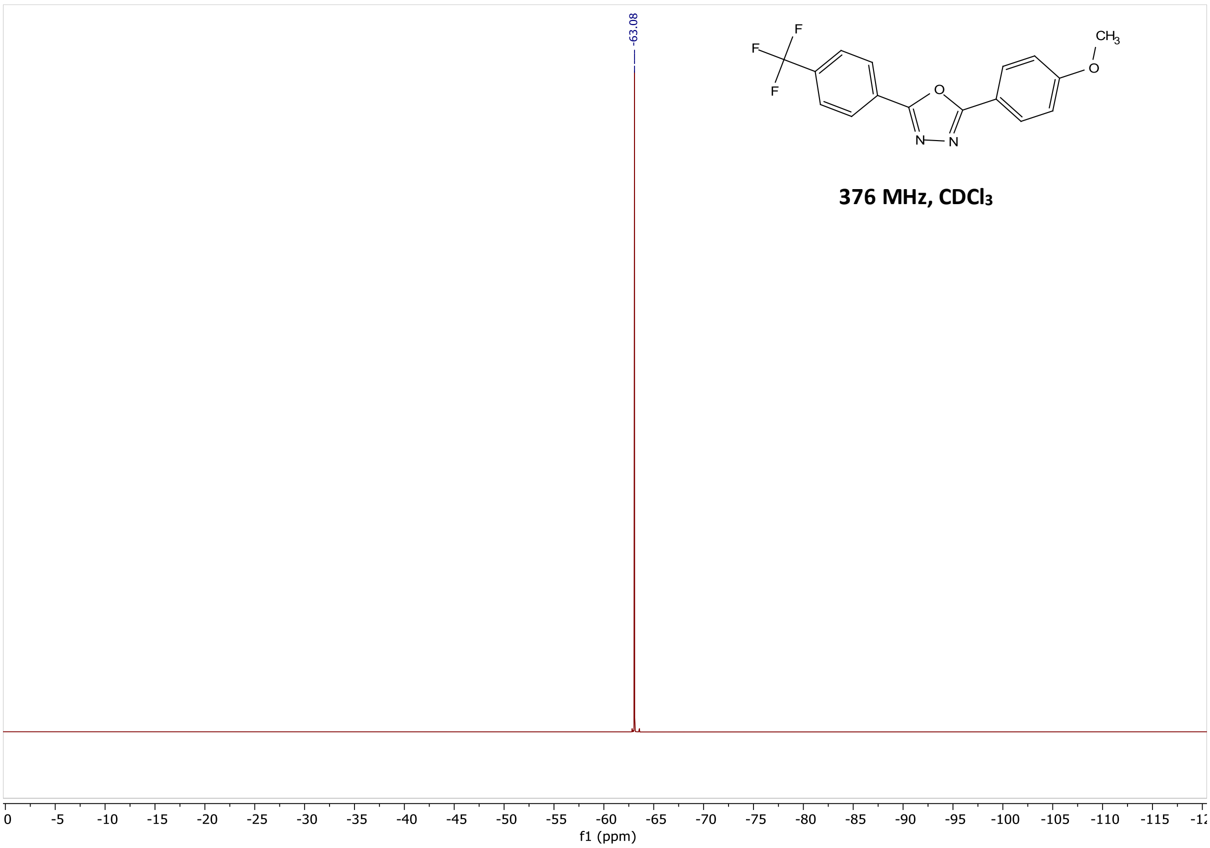
376 MHz, CDCl3
-63.08
f1 (ppm)

2-(4-Methoxyphenyl)-5-phenyl-1,3,4-oxadiazole, 2f, 500 MHz, CDCl$_3$:

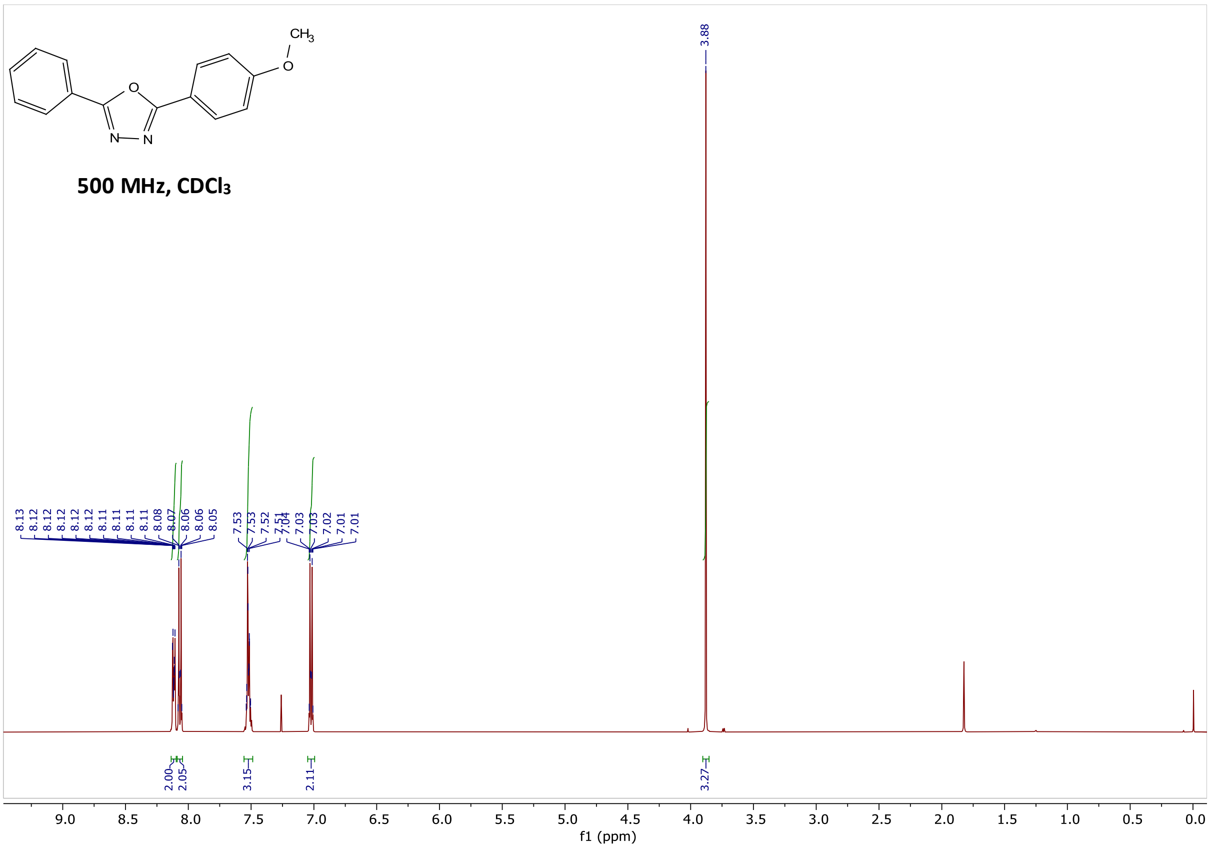

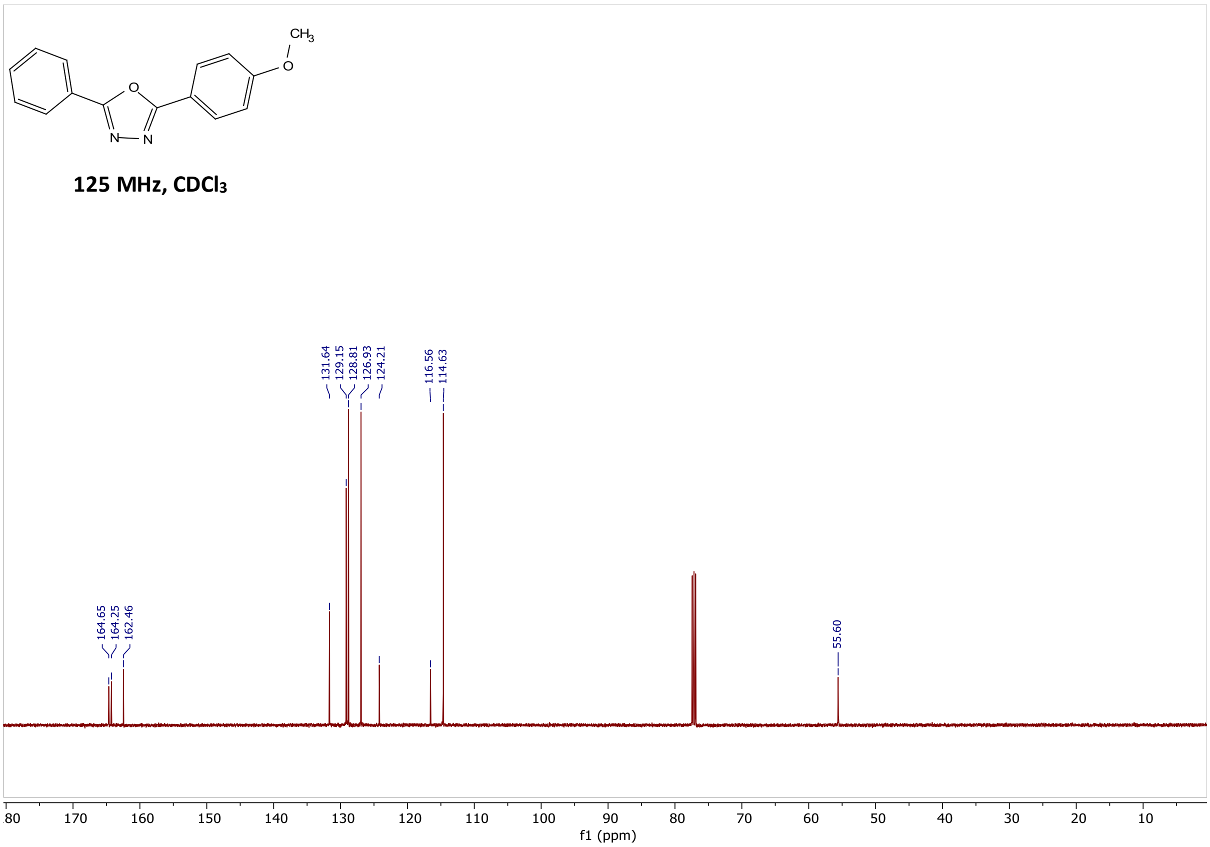

2-(2-Nitrophenyl)-5-(4-(trifluoromethyl)phenyl)-1,3,4-oxadiazole, 2g, 400 MHz, $CDCl_3$:

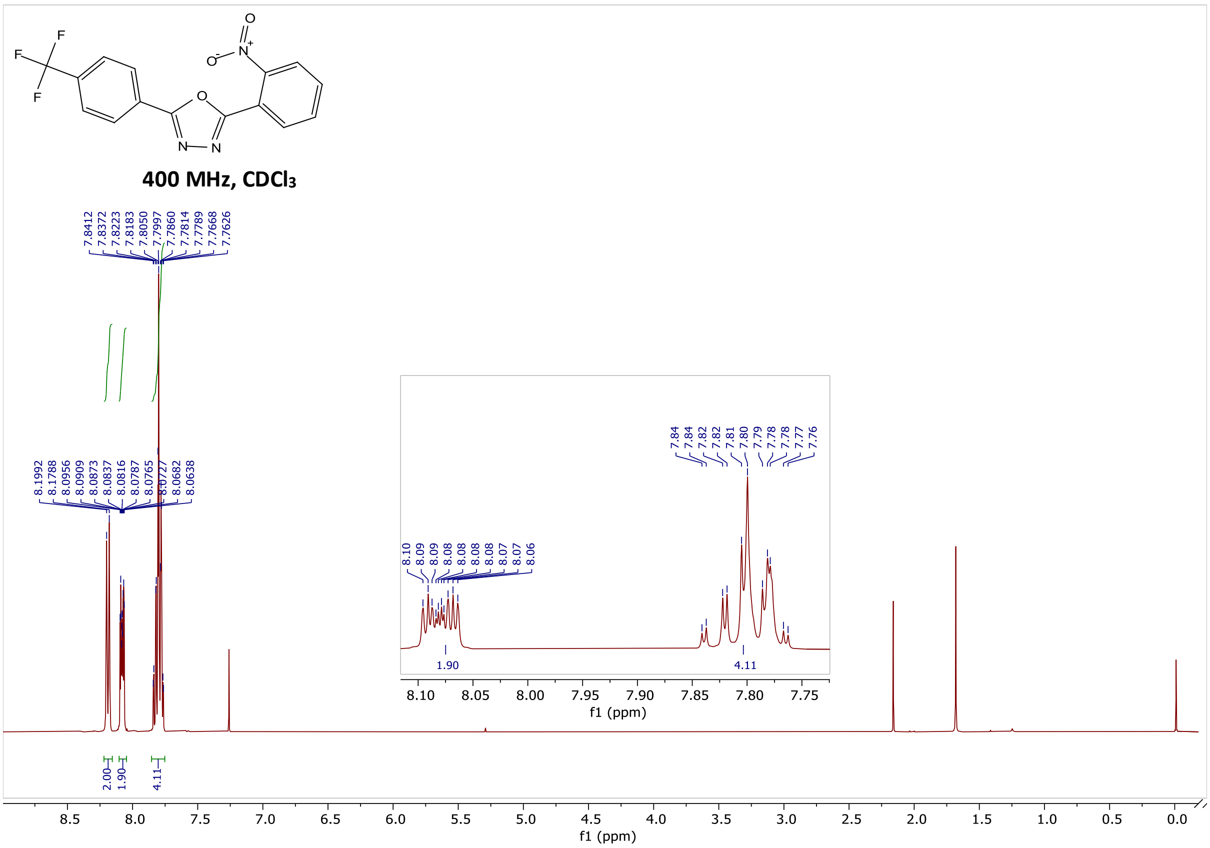

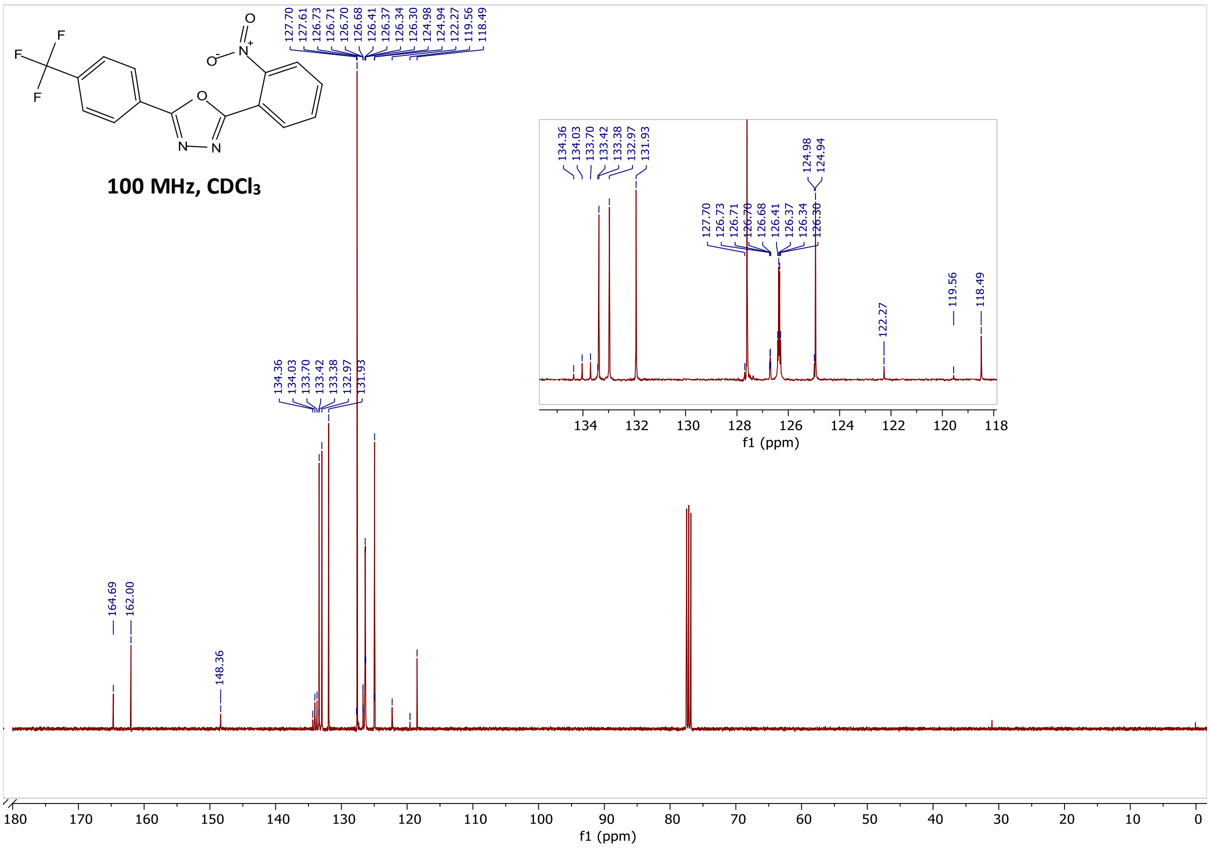

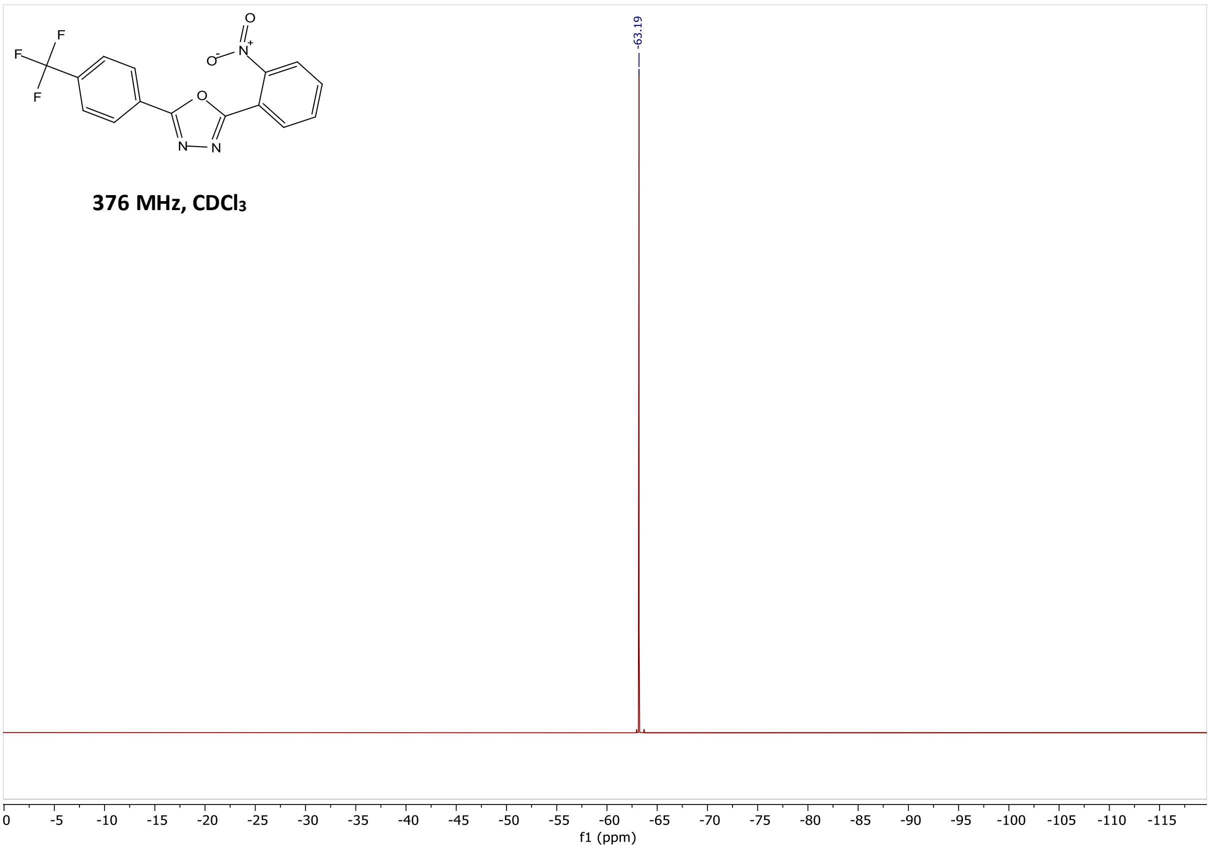

2-(2-Nitrophenyl)-5-phenyl-1,3,4-oxadiazole, 2h, 400 MHz, $CDCl_3$:

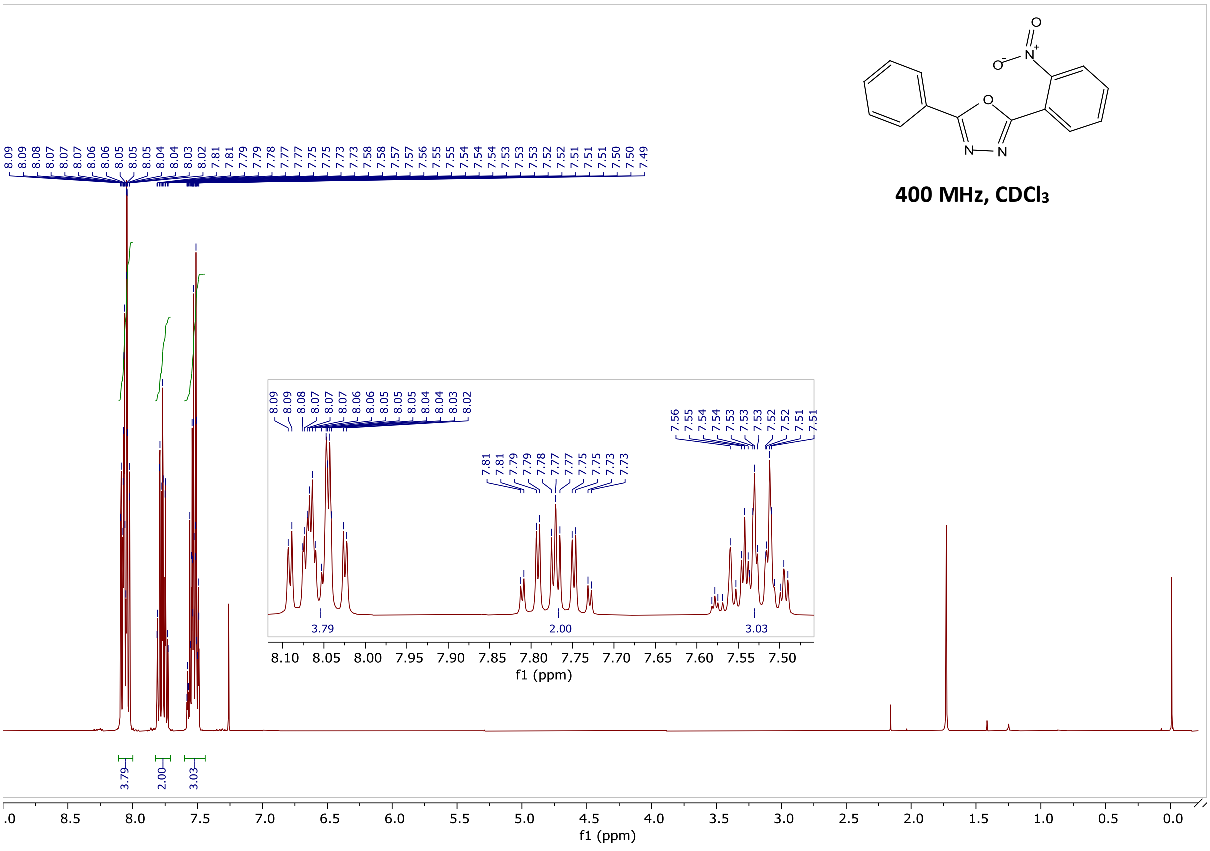

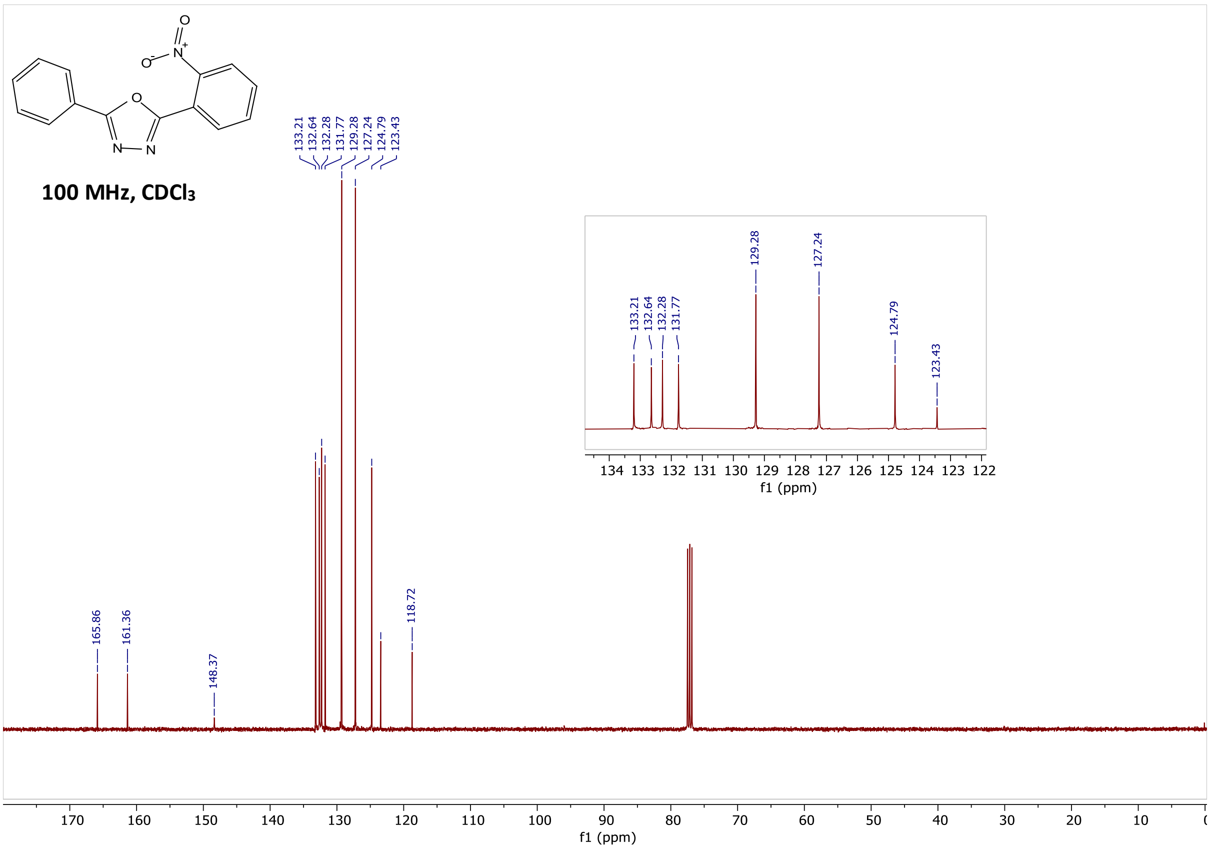

(*E*)-2-Styryl-5-(4-(trifluoromethyl)phenyl)-1,3,4-oxadiazole, 2i, 500 MHz, $CDCl_3$:

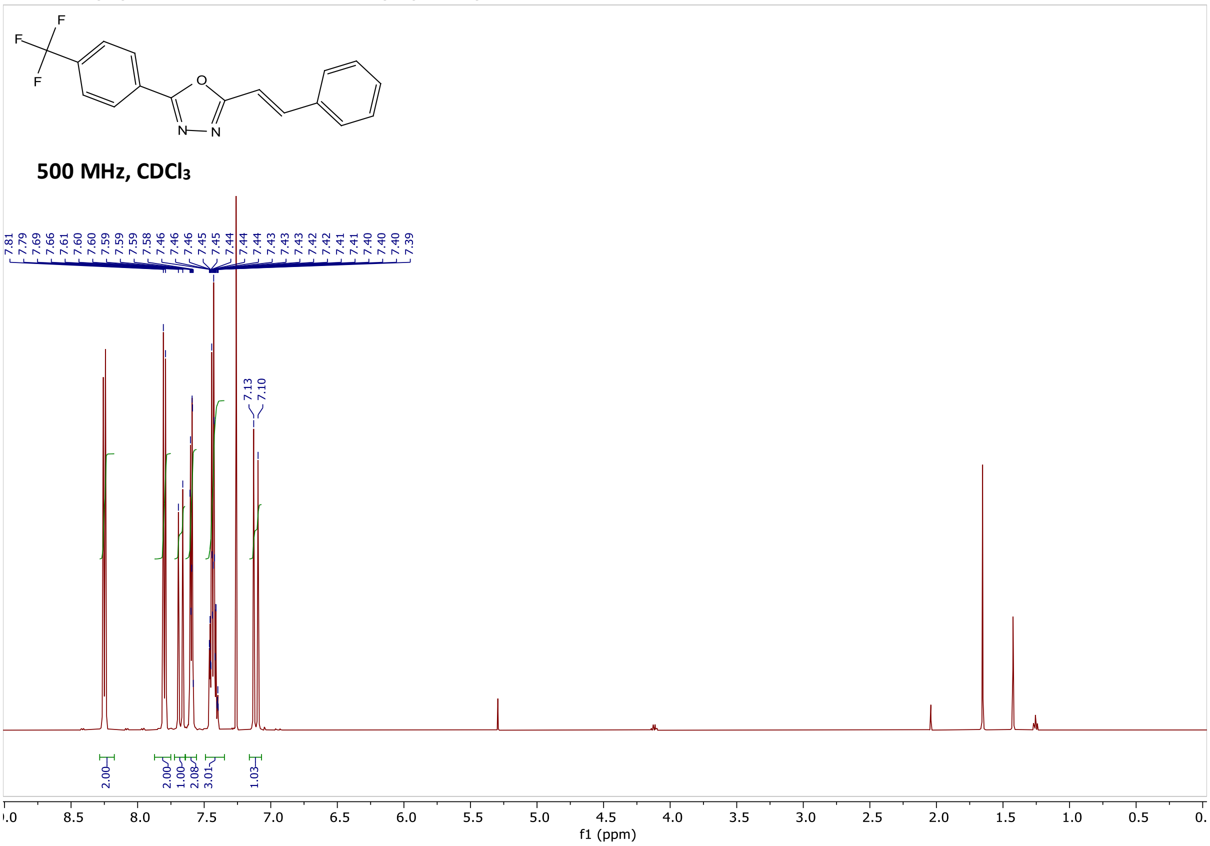

:

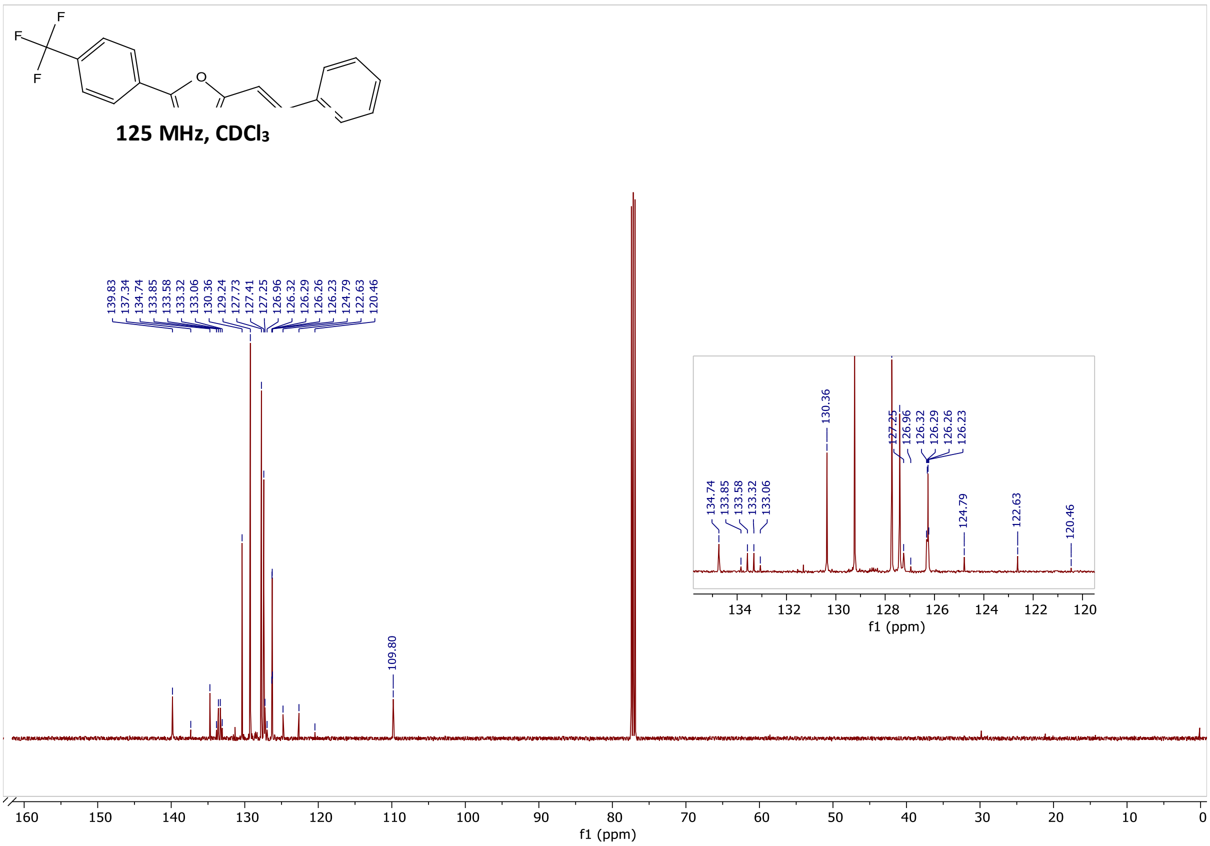
125 MHz, CDCl3
139.83
137.34
134.74
133.85
133.58
133.32
133.06
130.36
129.24
127.73
127.41
127.25
126.96
126.32
126.29
126.26
126.23
124.79
122.63
120.46
109.80
f1 (ppm)

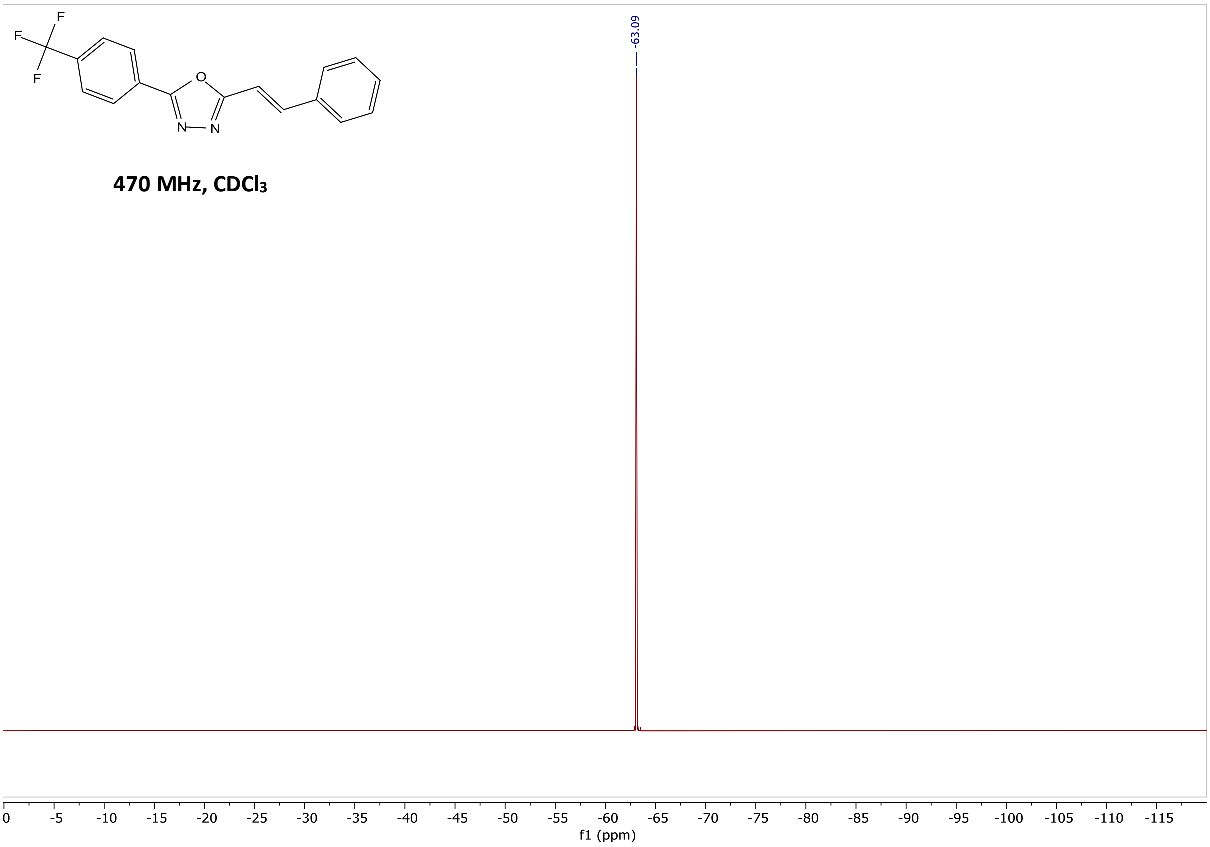
470 MHz, CDCl3
-63.09
f1 (ppm)

(*E*)-2-Phenyl-5-styryl-1,3,4-oxadiazole, 2j, 400 MHz, $CDCl_3$:

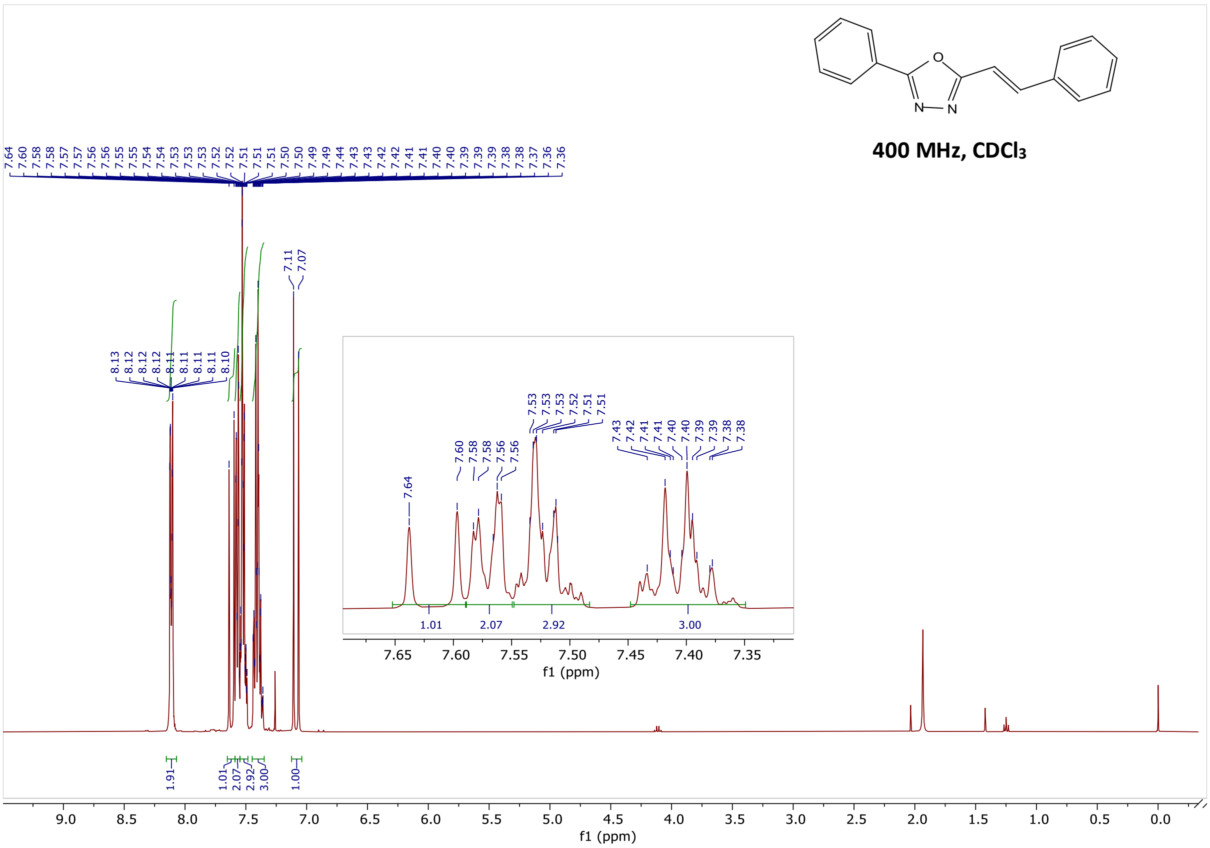

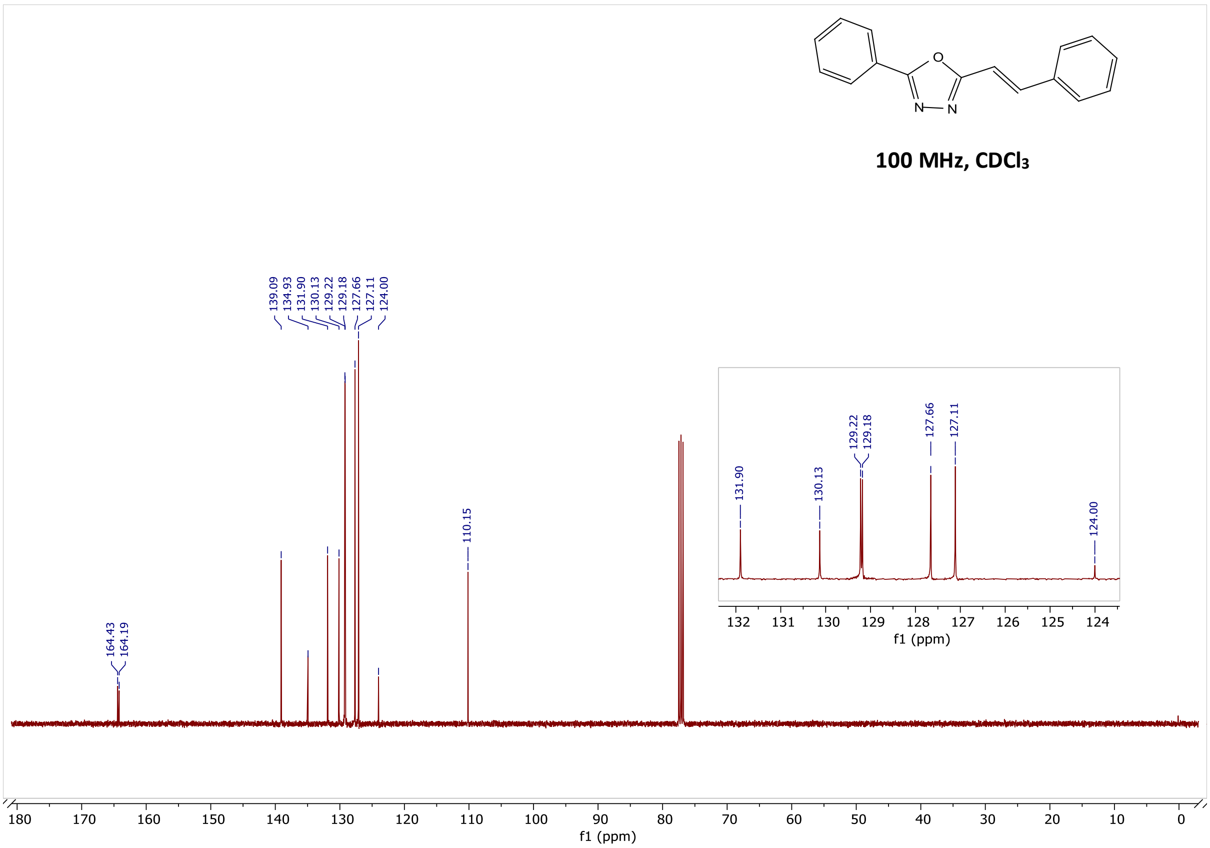

Isopropyl 3-(5-(thiophen-2-yl)-1,3,4-ozadiazol-2-yl)bicyclor[1.1.1]pentane-1-carboxylate, 2k, 500 MHz, $CDCl_3$:

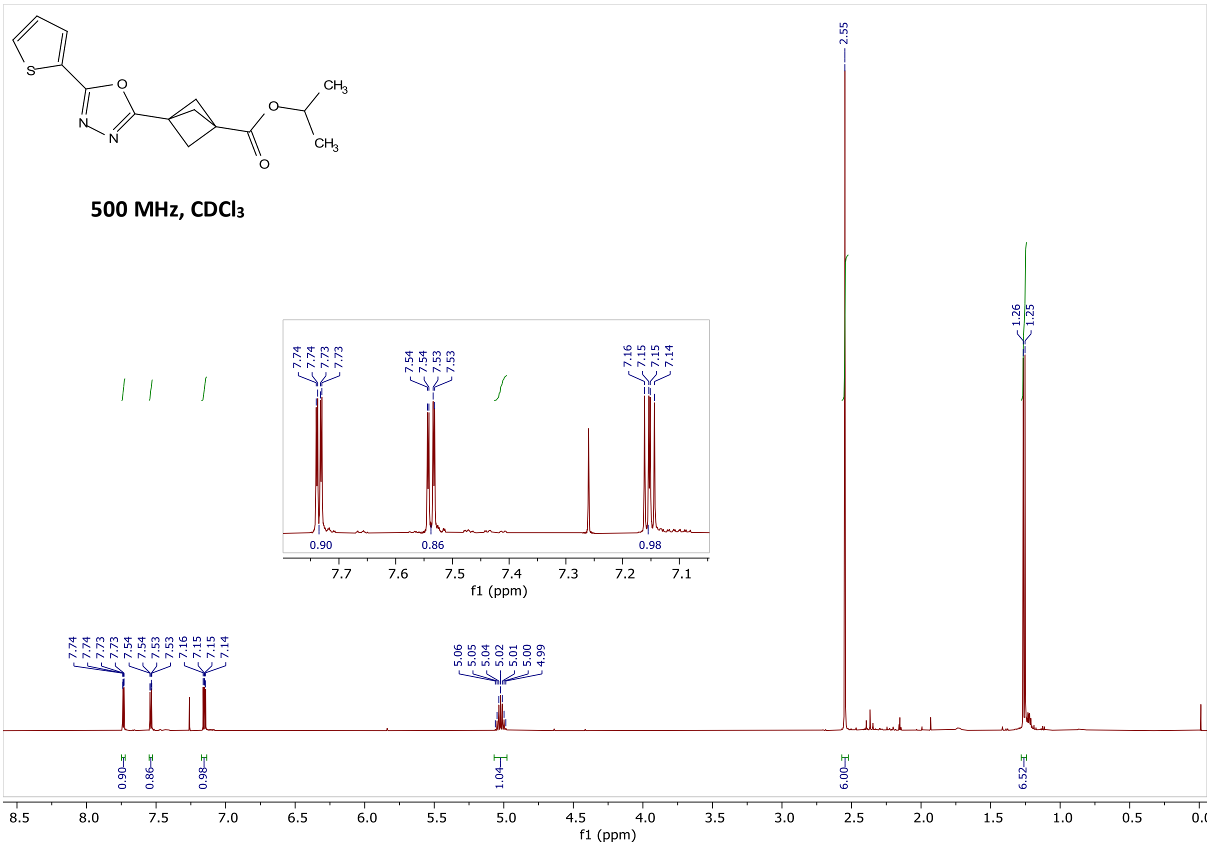

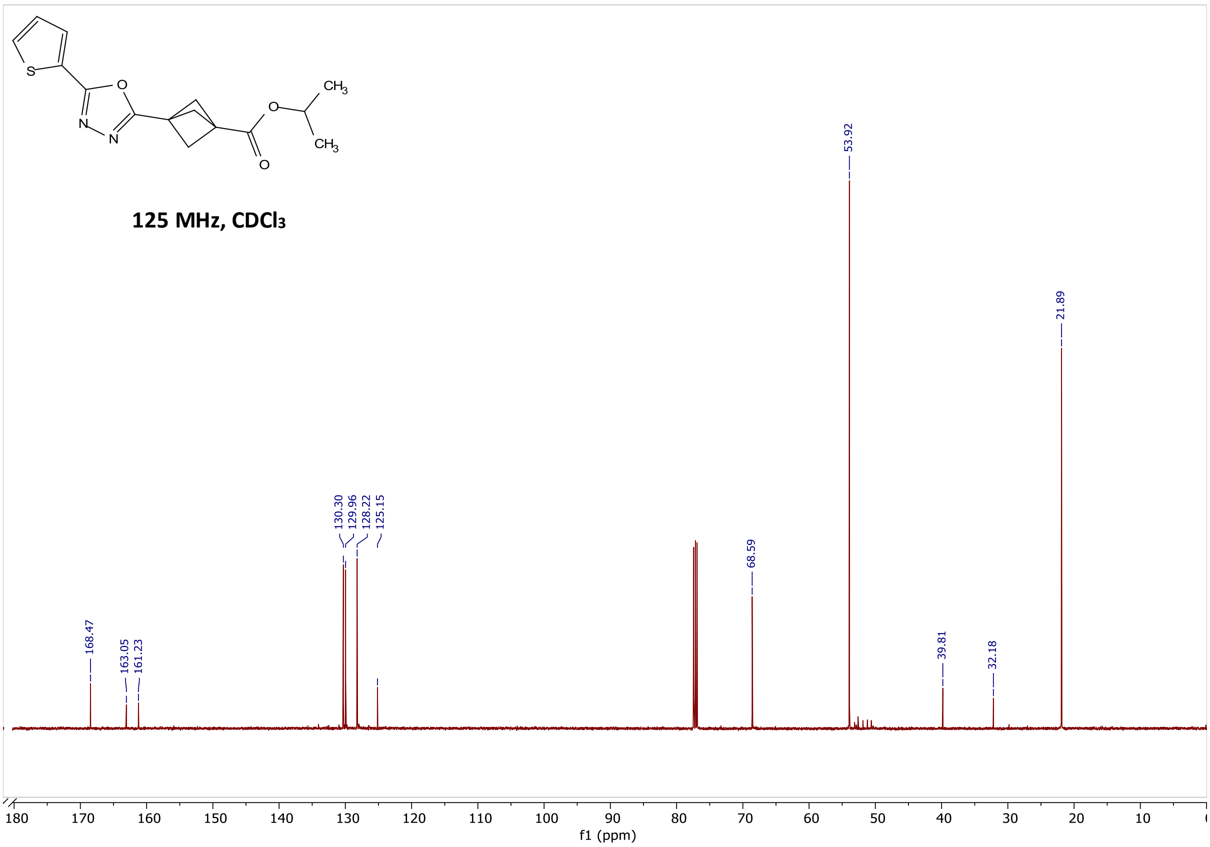

Isopropyl 3-(5-(4-bromophenyl)-1,3,4-oxadiazol-2-yl)bicyclo[1.1.1]pentane-1-carboxylate, 2l, 400 MHz, $CDCl_3$:

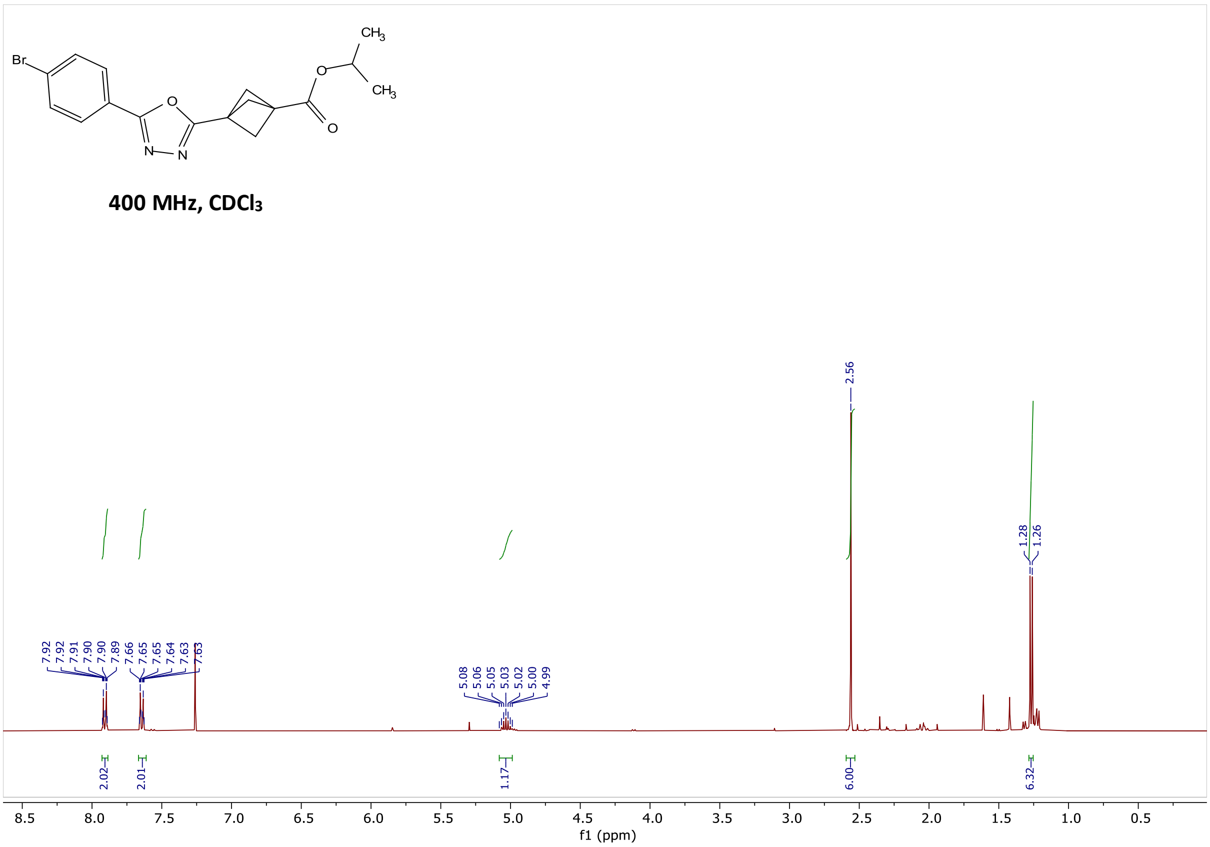

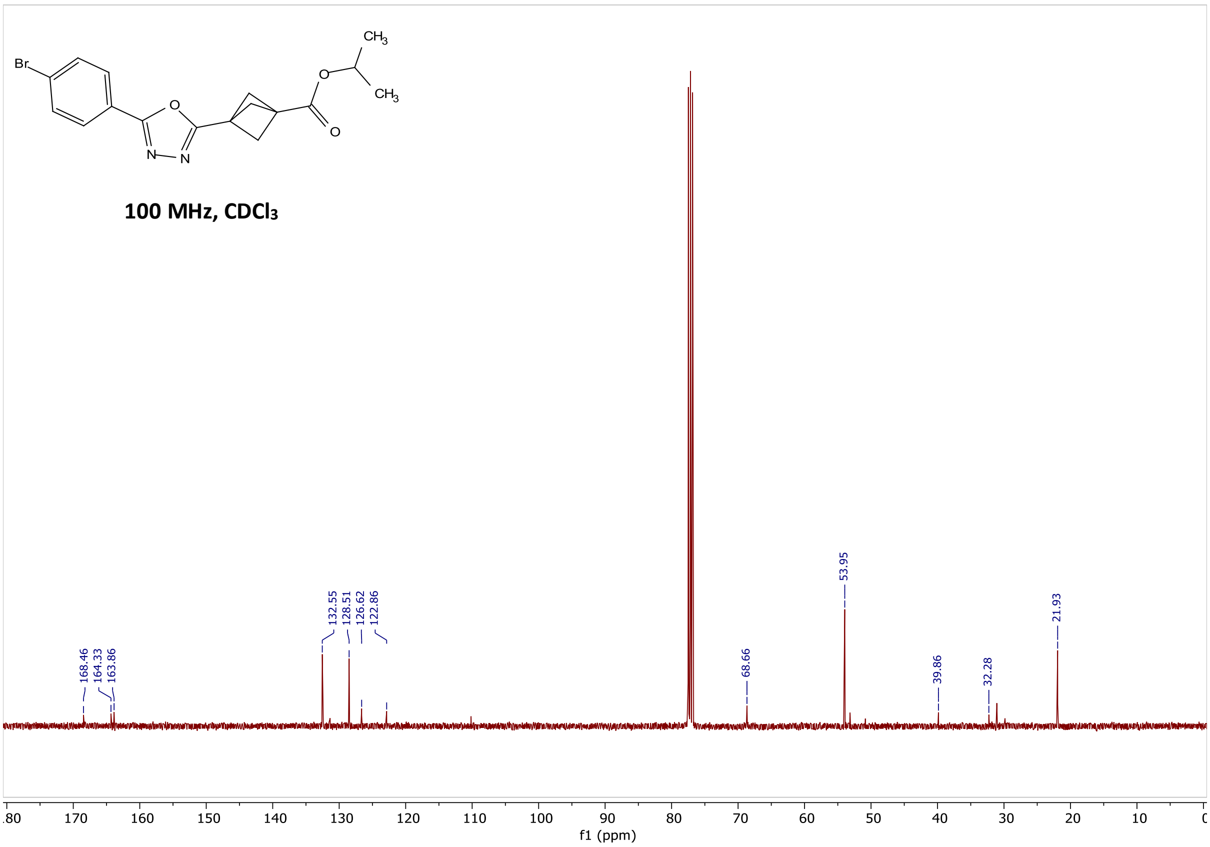

2-(5-(4-Bromophenyl)isoxazol-3-yl)-5-(thiophen-2-yl)-1,3,4-oxadiazole, 2m, 400 MHz, $CDCl_3$:

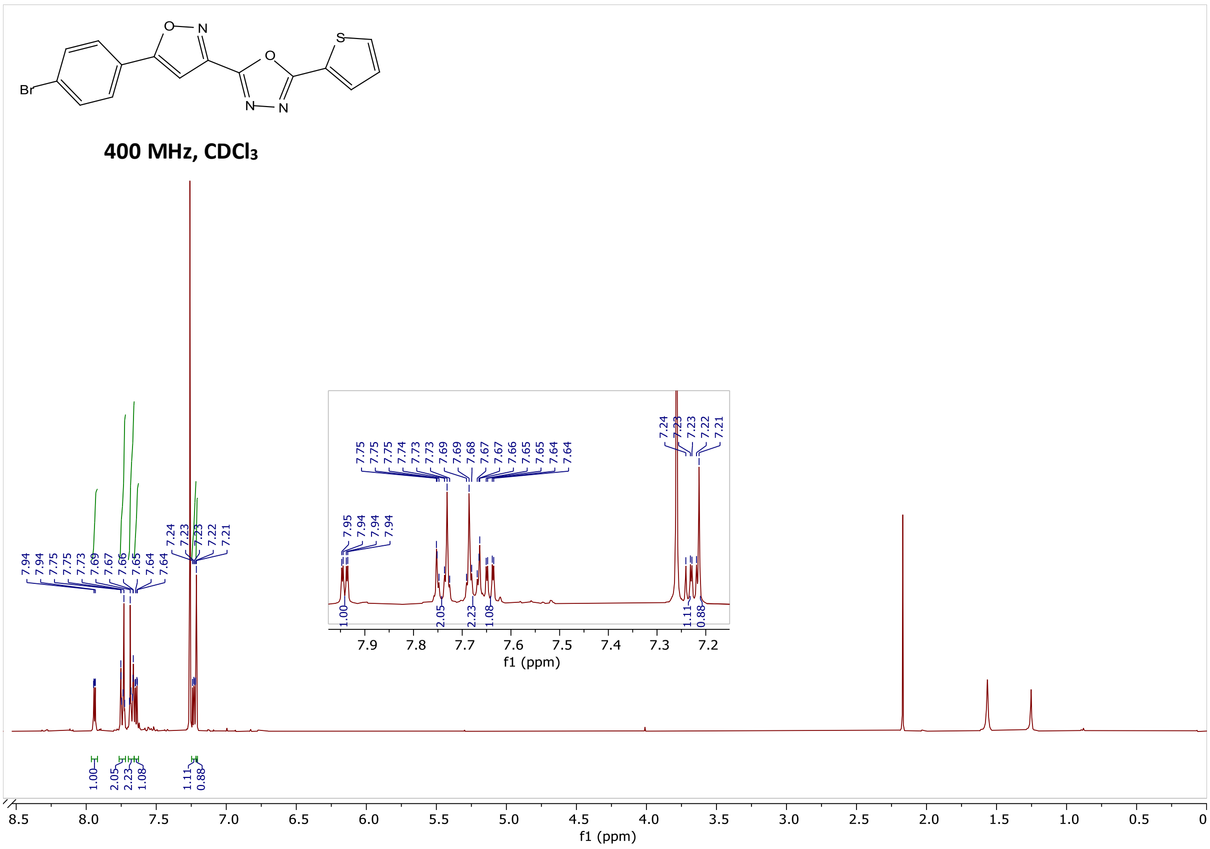

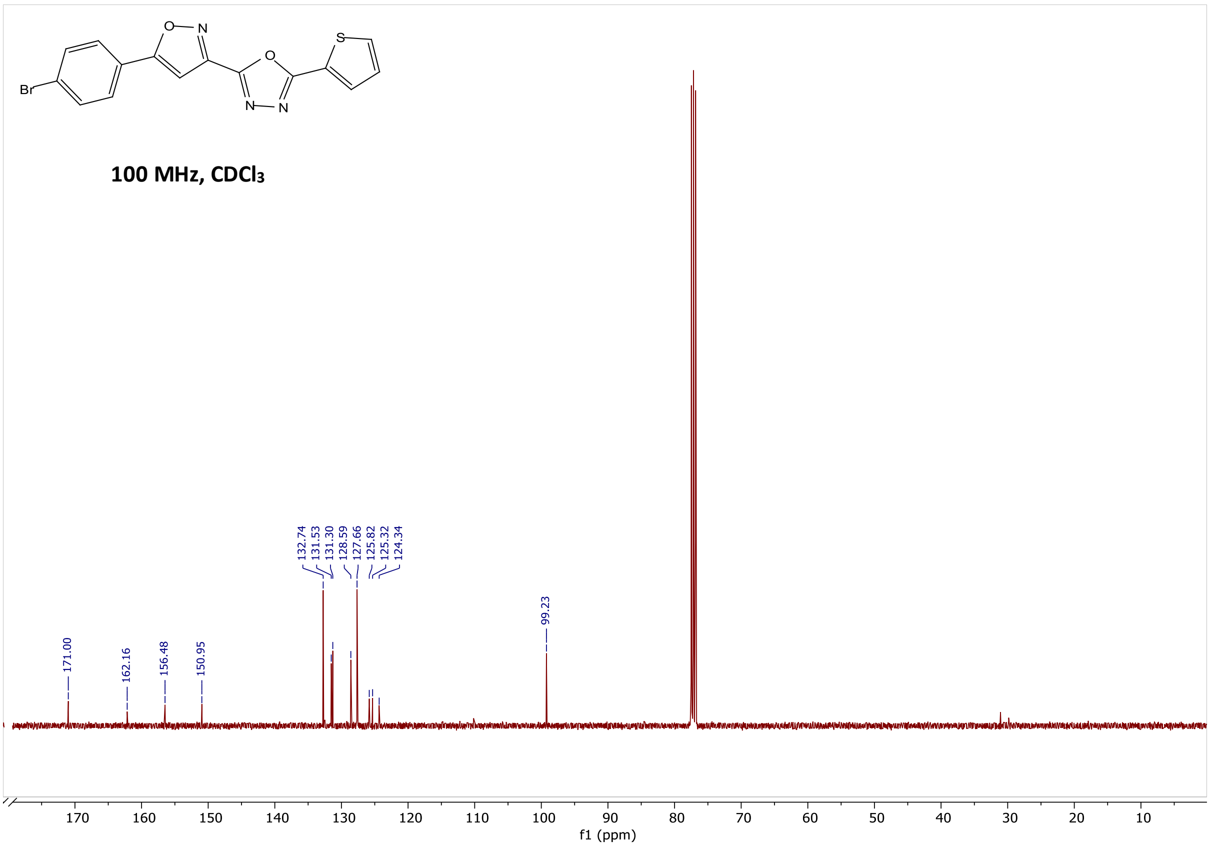

5-(4-Bromophenyl)isoxazole-3-carbohydrazide, 6, 400 MHz, DMSO-d_6

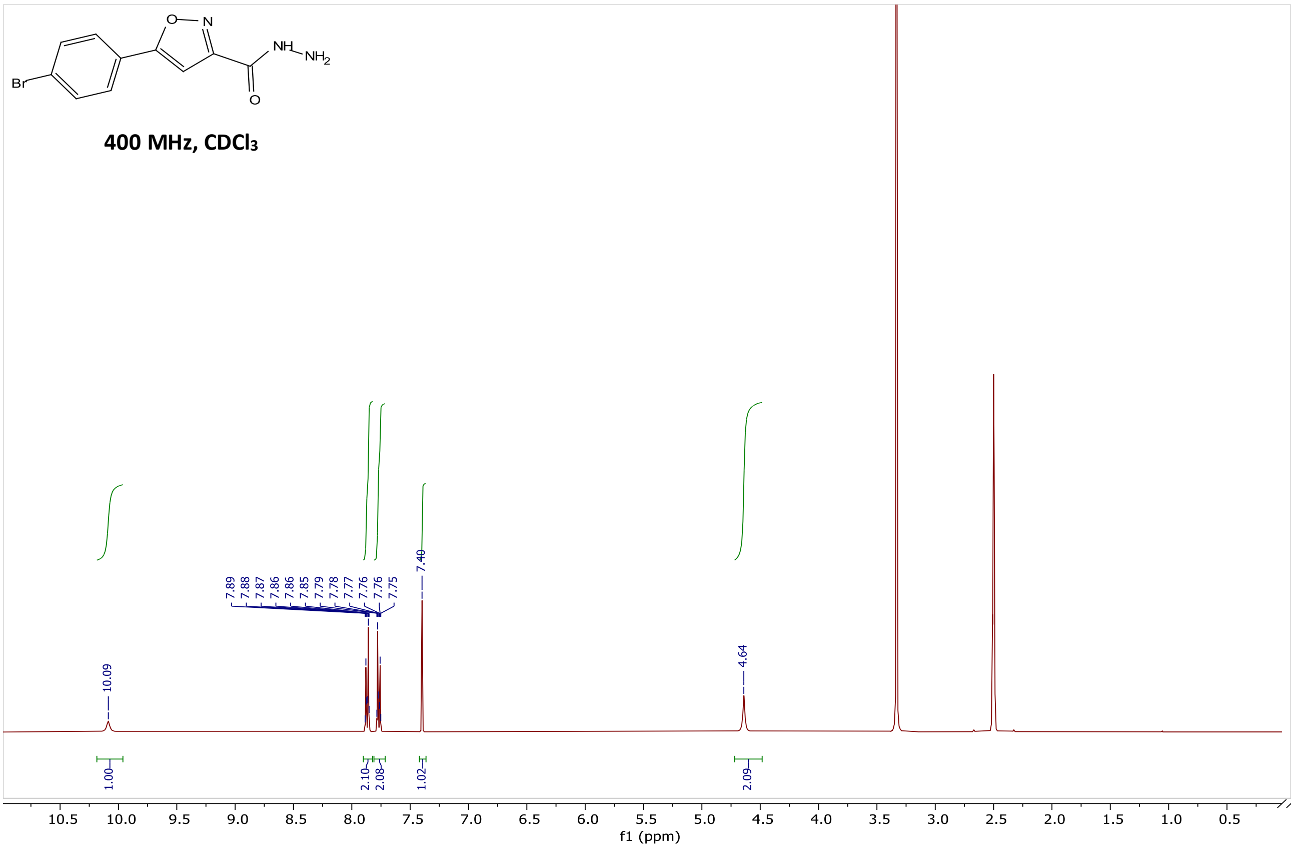

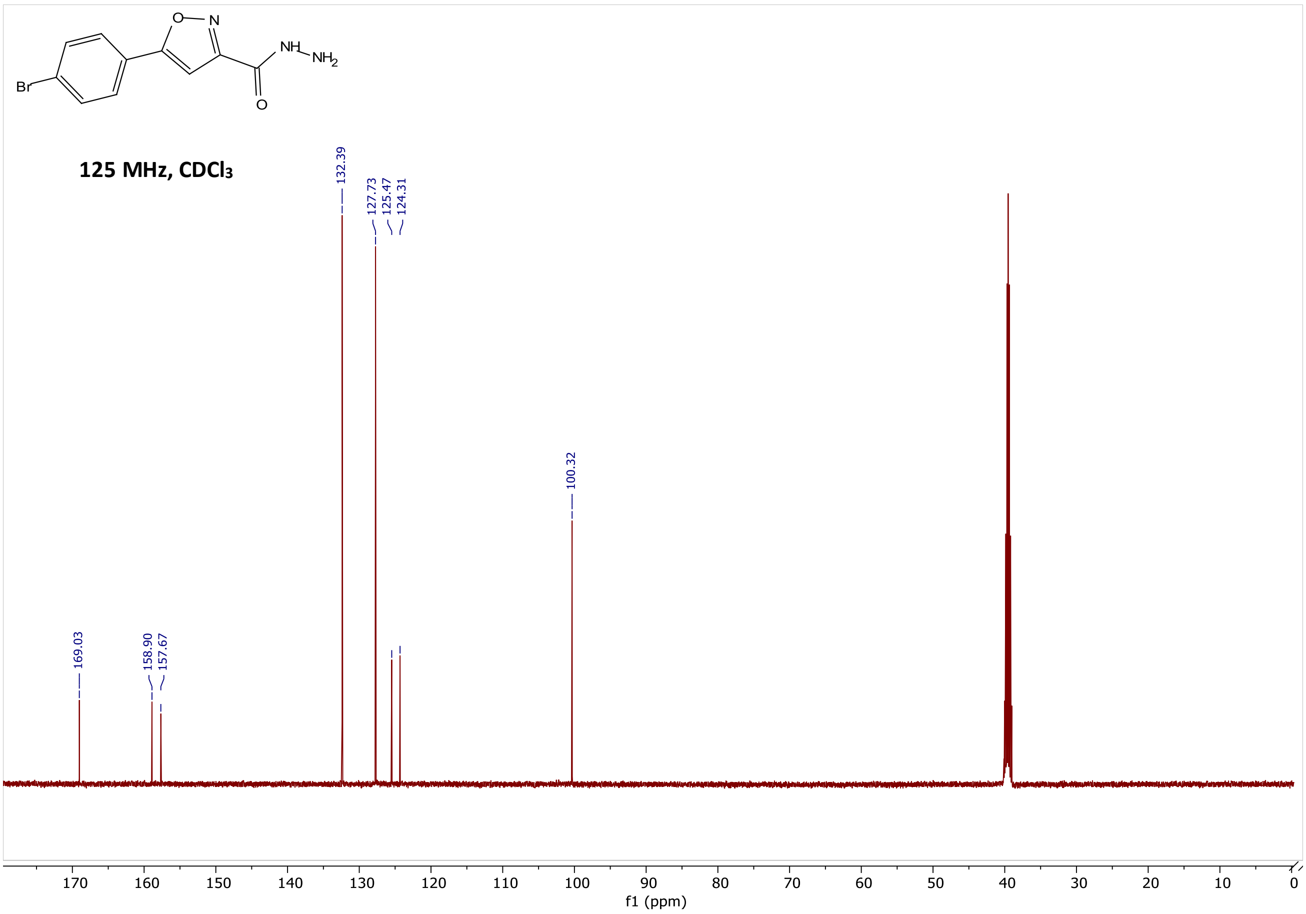

6. References:

1. van Dijken, D. J.; Kovaříček, P.; Ihrig, S. P.; Hecht, S., *J. Am. Chem. Soc.* **2015,***137*, 14982-14991. doi.org/10.1021/jacs.5b09519
2. Andrade, M. M.; Barros, M. T., Fast Synthesis of N-Acylhydrazones Employing a Microwave Assisted Neat Protocol. *J. Comb. Chem.* **2010,***12*, 245-247. doi.org/10.1021/cc9001444
3. Krátký, M.; Bősze, S.; Baranyai, Z.; Stolaříková, J.; Vinšová, J., *Bioorg. Med. Chem. Lett.* **2017,***27*, 5185-5189. doi.org/10.1016/j.bmcl.2017.10.050.
4. Delgado-Maldonado, T.; Nogueda-Torres, B.; Espinoza-Hicks, J. C.; Vázquez-Jiménez, L. K.; Paz-González, A. D.; Juárez-Saldívar, A.; Rivera, G., *Mol.Divers.***2020**. doi.org/10.1007/s11030-020-10156-5
5. Krátký, M.; Svrčková, K.; Vu, Q. A.; Štěpánková, Š.; Vinšová, J., *Molecules* **2021,***26,* 898. doi.org/10.3390/molecules26040989
6. Lee, T.; Landis, C. A.; Dhar, B. M.; Jung, B. J.; Sun, J.; Sarjeant, A.; Lee, H.-J.; Katz, H. E., *J. Am. Chem. Soc.***2009,***131*, 1692-1705. doi.org/10.1021/ja807219x
7. Gao, P.; Wang, J.; Bai, Z.; Cheng, H.; Xiao, J.; Lai, M.; Yang, D.; Fan, M., *Tetrahedron Lett.***2016,***57*, 4616-4619. doi.org/10.1016/j.tetlet.2016.09.007
8. Salva Reddy, N.; Raghavendar Reddy, P.; Das, B., *Synth.***2015,***47*, 2831-2838.doi.org/10.1055/s-0034-1380923
9. Holownia, A.; Tien, C.-H.; Diaz, D. B.; Larson, R. T.; Yudin, A. K.,*Angew. Chem. Int. Ed.***2019,***58*, 15148-15153. doi.org/10.1002/anie.201907486
10. Green, L.; Livingstone, K.; Bertrand, S.; Peace, S.; Jamieson, C., *Chem. Eur. J.***2020,***26*, 14866-14870. doi.org/10.1002/chem.202002896
11. Yu, W.; Huang, G.; Zhang, Y.; Liu, H.; Dong, L.; Yu, X.; Li, Y.; Chang, J., *J. Org. Chem.***2013,***78*, 10337-10343. doi.org/10.1021/jo401751h
12. Gnanasekaran, K. K.; Nammalwar, B.; Murie, M.; Bunce, R. A.,*Tetrahedron Lett.* **2014,***55*, 6776-6778. doi.org/10.1016/j.tetlet.2014.10.028
